# Synthesis, Biological Evaluation, and Computational Study of Pyridine- and Indazole-Based Inhibitors of the Inducible Nitric Oxide Synthase as Promising Antipsoriatic Agents

**DOI:** 10.1021/acsptsci.5c00683

**Published:** 2026-04-22

**Authors:** Pasquale Amoia, Marialucia Gallorini, Claudia Scarponi, Francisco Franco-Montalban, Patrizia Bonfanti, Anita Emilia Colombo, Valentina Di Francesco, Stefania Madonna, Alessandra Ammazzalorso, Barbara De Filippis, Letizia Giampietro, Amelia Cataldi, Rosa Amoroso, Cristina Albanesi, Cristina Maccallini

**Affiliations:** † Department of Pharmacy, 9301 University “G.d’Annunzio” of Chieti-Pescara, Via dei Vestini 31, 66100 Chieti, Italy; ‡ Laboratory of Experimental Immunology, 9363 Istituto Dermopatico dell’Immacolata, IDI-IRCCS, Via Monti di Creta 104, 00167 Rome, Italy; § Department of Medicinal and Organic Chemistry, Faculty of Pharmacy, 16741 University of Granada, Campus Cartuja s/n, 18071 Granada, Spain; ∥ POLARIS Research Center, Department of Earth and Environmental Sciences, University of Milano-Bicocca, Piazza della Scienza 1, 20126 Milan, Italy

**Keywords:** inducible nitric oxide synthase, inflammation, keratinocytes, macrophages, psoriasis, synthesis, zebrafish embryos

## Abstract

The dysregulation of inducible nitric oxide synthase (iNOS) is linked to various diseases, including psoriasis, where it contributes to imbalanced nitro-oxidative stress. iNOS is primarily produced in the skin’s epidermal layer and can be activated by cytokines elevated in psoriatic lesions, such as TNF-α and IL-17. Macrophages also play a role in psoriasis by producing cytokines and iNOS, and patients show increased levels of these immune cells in lesions. Given the association of iNOS with psoriasis severity, it is seen as a potential therapeutic target. However, no specific iNOS inhibitor has been reported as a treatment for psoriasis. The study describes the synthesis of new compounds based on prior iNOS inhibitors, their potency and selectivity of action, and the evaluation of the most interesting compounds in different *in vitro* and *ex vivo* cell models of psoriasis. Moreover, a computational analysis was performed that sheds light on the binding mode of the most promising molecule into both the iNOS and the constitutive endothelial NOS (eNOS). Compound **10** demonstrated significant effectiveness with respect to known iNOS inhibitors, reducing nitric oxide release, cytokine-induced inflammation, and cell necrosis, also shifting macrophages from a pro-inflammatory to a resolving phenotype. Its reasonable metabolic stability, along with the absence of significant *in vivo* toxicity, supports its further evaluation as a promising candidate for antipsoriatic drug development.

## Graphical abstract

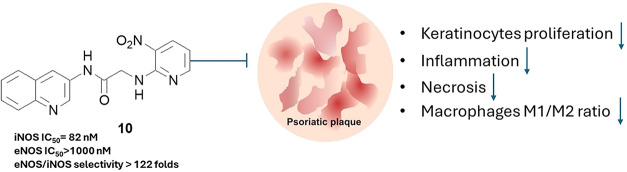

Psoriasis is a systemic inflammatory skin condition affecting approximately 2% of the world’s population.^1^ This disease is characterized by red or discolored, scaly, and itchy patches on the skin, producing high discomfort in patients and impairing their quality life. Moreover, the severe psoriasis forms are connected to systemic inflammation, and to the development of cardiovascular diseases, psoriatic arthritis, and other comorbidities.^2^ Psoriasis is characterized by abnormal keratinocyte differentiation and hyperproliferation, as well as production of inflammatory mediators by immune cell infiltration, such as macrophages, dendritic cells, and T lymphocytes. Therefore, the pathogenesis of psoriasis involves both the dysregulation of immunological cell function as well as the keratinocyte proliferation/differentiation.^3^ Advances in the understanding of the psoriasis pathophysiology have led to the development of multiple therapeutic options, which include topical agents (vitamin D analogs and corticosteroids), phototherapy, systemic drugs (methotrexate, ciclosporin, dimethyl fumarate, and apremilast), and biologics (TNF-α, IL-17, and IL-23 inhibitors).^4–6^ Nevertheless, because psoriasis is a heterogeneous disease, there is the need to update the treatment arsenal with new targeted therapies.

The inducible nitric oxide synthase (iNOS) is an important enzyme belonging to a family of oxidoreductases, i.e., the nitric oxide synthases (NOS), and it is widely expressed by different immune cells. The NOS family encompasses also two constitutive isoforms, i.e., the neuronal NOS (nNOS) and the endothelial NOS (eNOS), and it is responsible for the conversion of l-arginine into l-citrulline and nitric oxide (NO).^7^ The last is an important biological messenger playing multiple roles, in both cell physiology and pathology, and it has a pivotal role in the innate immune response. The dysregulation of iNOS is associated with the development of different diseases, and it is considered to play a role in the pathogenesis of psoriasis, as well as the imbalanced nitro-oxidative stress.^8–10^ iNOS is generated primarily in the epidermal layer of skin and can be induced by TNF-α, interleukin (IL)-1β, IL-2, IL-6, and many other cytokines, which are increased in psoriatic skin lesions. In particular, TNF-α activates the nuclear factor (NF)-kB signal pathway, affecting cell survival and proliferation of lymphocytes and keratinocytes.^11^ The last are stimulated also to produce IL-8, which enhances neutrophil recruitment in psoriasis, with the consequent formation of microabscesses.^12^ Moreover, it has been indicated that macrophages participate to the pathogenesis of psoriasis producing IL-23, IL12, TNF-α, and iNOS.^13,14^ Indeed, the peripheral blood of patients have high levels of monocytes, and an increased number of macrophages is found in psoriatic lesions.^15,16^ In particular, the ratio of M1/M2 macrophages polarization is higher in the skin plaque with respect to controls.^17^

Given the involvement of iNOS in the pathogenesis of psoriasis, as well as the correlation between the serum NO levels and the disease severity,^18^ it could be inferred that this enzyme represents an effective target to menage psoriasis. However, to the best of our knowledge, no iNOS inhibitor has yet been reported as an antipsoriatic agent, probably also due to the unfavorable safety profile often shown by the known iNOS inhibitors, especially on the cardiovascular system.^19^

Nitrogen-containing heterocycles such as 2-aminopyridine, indazole, and quinoline are well-established pharmacophoric units in different NOS inhibitors (Figure 1A).^20–26^ These heterocycles effectively mimic the binding mode of the natural substrate l-arginine by providing appropriately positioned hydrogen-bond donors and acceptors, while their aromatic and heteroaromatic frameworks enable π–π interactions with the heme prosthetic group and nearby hydrophobic residues. The presence of ring nitrogens allows modulation of basicity and p*K*_a_, facilitating favorable electrostatic interactions with conserved acidic residues and, in some cases, weak coordination to the heme iron, thereby disrupting oxygen activation required for nitric oxide formation.^9^ Importantly, these scaffolds permit fine structural tuning to exploit subtle differences among NOS isoforms, contributing to improved potency and isoform selectivity, particularly over eNOS. In fact, while iNOS-derived NO is associated with pathological inflammation and oxidative stress, eNOS plays a crucial physiological role in maintaining vascular homeostasis, including the regulation of vasodilation, blood pressure, and endothelial function. Therefore, unintended inhibition of eNOS may lead to adverse cardiovascular effects such as impaired vasodilation and hypertension. For this reason, selective inhibition of iNOS over eNOS is generally preferred to achieve anti-inflammatory effects while minimizing potential vascular side effects.^7,9^ Building on this knowledge, and since both lipophilic and ionizable groups are recognized as key structural requirements for iNOS inhibition,^27–29^ in the present study, we designed new molecules by hybridizing selected nitrogen-containing heterocyclics with structural fragments from known inhibitors, with the dual aim of achieving high potency of action and improved lipophilicity (Figure 1B,C). The promising inhibitory activity observed for several of these newly designed hybrids encouraged us to explore their potential therapeutic value in psoriasis management, which we describe in detail below.

**Figure 1:**
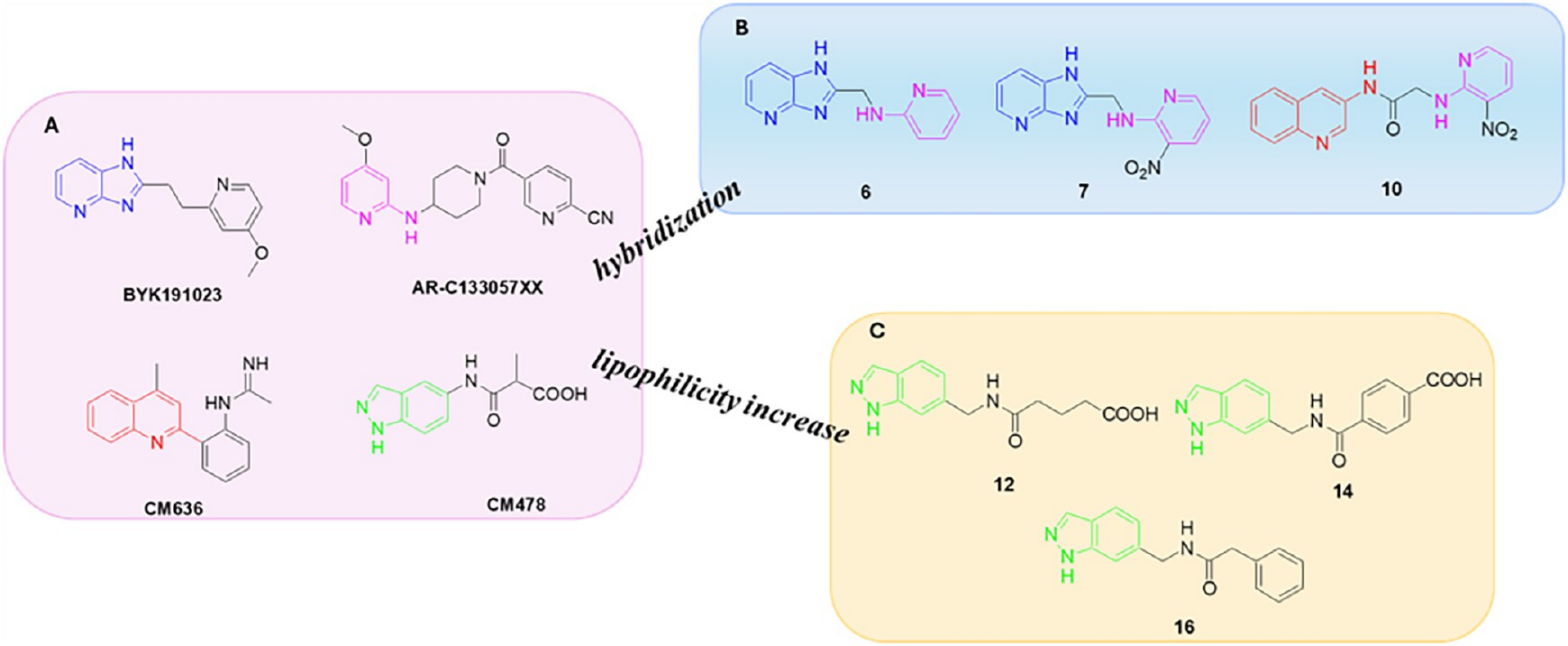
Chemical structure of the iNOS inhibitors. (A) Chemical structures of known iNOS inhibitors bearing different heterocyclic moieties. (B and C) Chemical structures of target compounds **6**,**7**,**10** and **12**,**14**,**16**.

## Chemistry

To obtain imidazopyridines **6** and **7**, the synthetic route reported in Scheme 1 was followed. Briefly, 2,3 diamino-pyridine was coupled with Z-glycine, and then intermediate **3** was cyclized to form imidazopyridine **4** by means of glacial acetic acid treatment. The benzyloxycarbonyl group was removed by catalytic hydrogenation, and the obtained amine **5** was condensed with 2-chloropyridine and 2-chloro-3-nitropyridine, obtaining the desired compounds **6** and **7**, respectively.

**Scheme 1:**
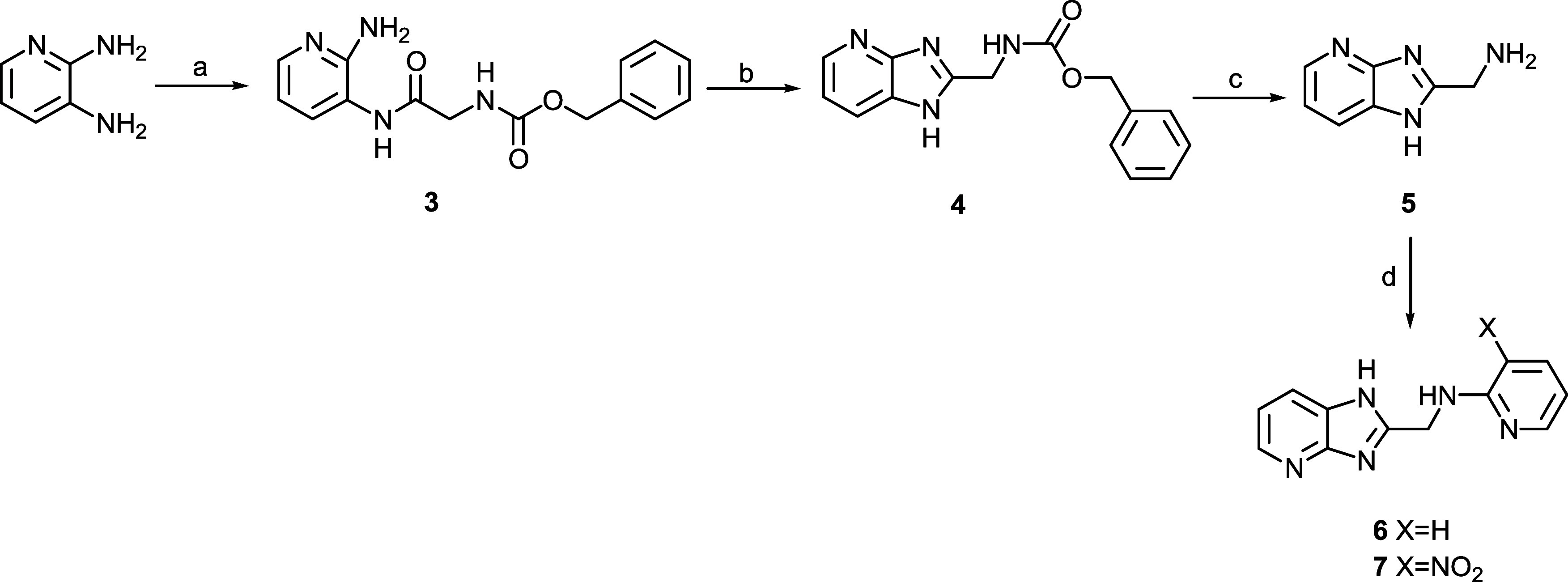
Synthesis of the Target Compounds **6** and **7** ^a^

a Reagents and conditions: a: EDC HCl, OHBt, DMAP, Z-Gly, DMF dry, N_2_, from 0 °C to r.t., 20 h; b: CH_3_COOH, b.t., 20 h; c: Pd/C, H_2_ (1 bar), CH_3_OH, r.t. N_2_, 3.5 h; d: 2-chloropyridine or 2-chloro-3-nitropyridine, TEA, DMF, 90° C, 4 h.

To prepare the quinoline derivative **10**, 2-chloro-3-nitropyridine was coupled with glycine methyl ester. The obtained **8** was hydrolyzed to carboxylic acid intermediate **9**, which was finally condensed with 3-aminoquinoline (Scheme 2).

**Scheme 2:**
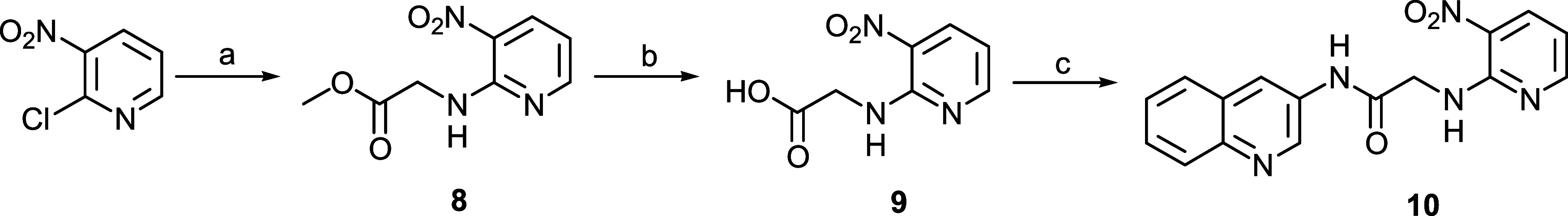
Synthesis of the Target Compound **10** ^a^

a Reagents and conditions: a: GlyOMe, 2-chloro-3-nitropyridine, TEA, DMF, 90° C, 3 h; b: NaOH 5% (w/v), CH_3_OH, r.t., 3 h; c: i-BuOCOCl, NMM, 3-aminoquinoline, N_2_, from −15 to 0 °C for 20 h, then r.t. for 4 h.

Finally, the synthesis of indazoles **12**, **14**, and **16** was performed according to Scheme 3. The 6-cyanoindazole was reduced to intermediate amine **11** which was coupled with phenylacetic acid to give **12**, or with both monoprotected succinic acid and terephthalic acid, affording intermediates **13** and **15**, respectively. These two compounds were finally deprotected to give compounds **14** and **16**.

**Scheme 3:**
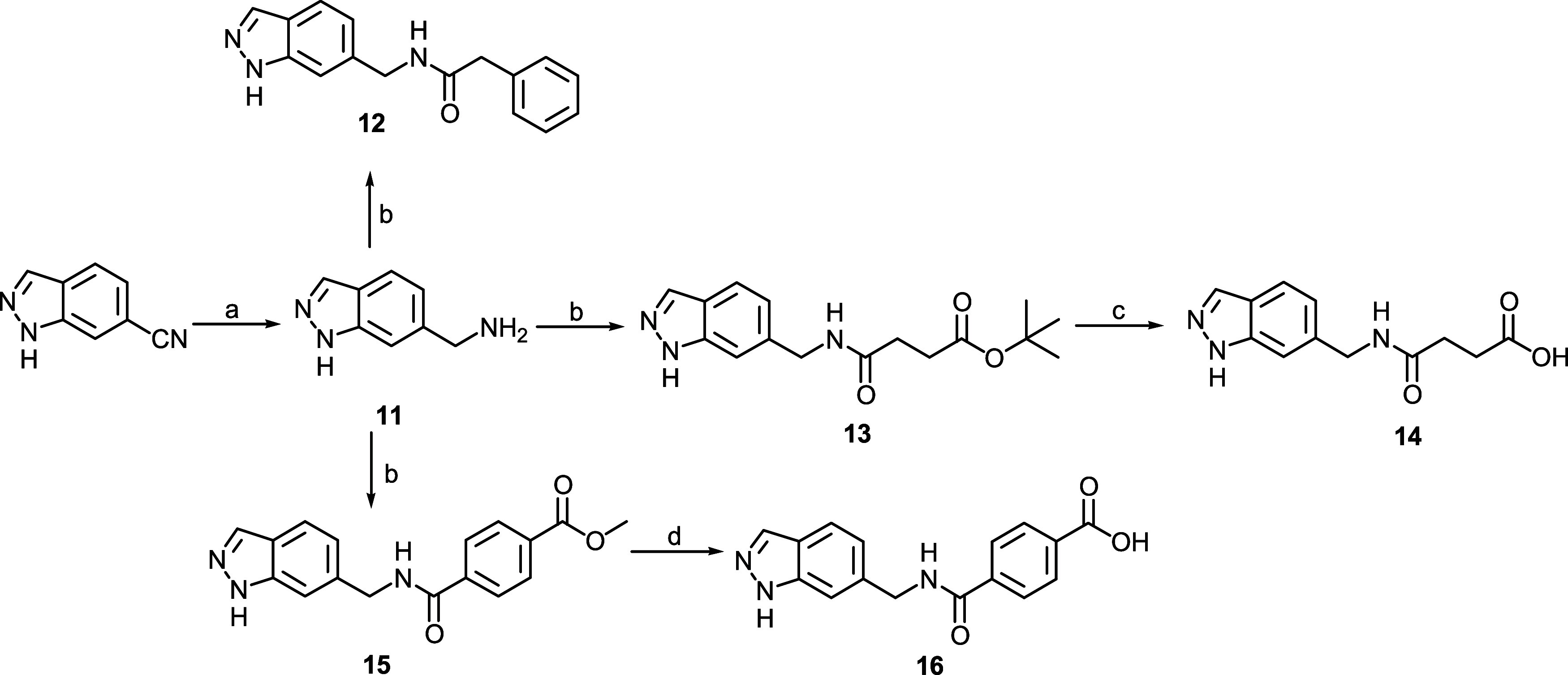
Synthesis of the Target Compounds **12**, **14**, and **16^a^**

a Reagents and Conditions: a: LiAlH_4_ 2 M, THF dry, N_2_, from 0 °C to r.t., 2h; b: EDC HCl, DMAP, phenylacetic acid or *t*-Bu­(O)­succinic acid or monomethyl terephthalate, DMF dry, N_2_, from 0 °C to r.t., 24 h; c: CF_3_COOH, CH_2_Cl_2_, from 0 °C to r.t., 12 h; d: NaOH 5% (w/v), THF, r.t., 1 h.

## Results and Discussion

### Nitric Oxide Synthase Activity

All of the synthesized compounds were evaluated as iNOS inhibitors, by using the l-citrulline assay with fluorimetric detection.^30^ In order to screen the compounds’ activity and to determine their isoform selectivity, they were evaluated at 1 μM against the human iNOS (hiNOS), and at 10 μM against the bovine eNOS (beNOS). The choice of hiNOS and beNOS isoforms is supported by the fact that isoforms from different species share a high degree of sequence and structural homology, especially within their catalytic domains, highlighting the strong evolutionary conservation of their enzymatic function.^31^ Selectivity profiling of the new compounds was focused on iNOS and eNOS, while nNOS was not investigated, based on its biological relevance in psoriasis development.^32^ In fact, nNOS is predominantly expressed in neuronal tissues and is mainly involved in neurotransmission rather than in the inflammatory mechanisms underlying psoriasis. Moreover, the in silico prediction of physicochemical and pharmacokinetic properties of target compounds (Table S2) consistently predicted no blood–brain barrier permeation for the most promising compounds, indicating a low likelihood of central nervous system exposure and, consequently, limited interaction with nNOS *in vivo*. Therefore, given the therapeutic focus on iNOS-driven skin inflammation and the need to assess selectivity versus constitutive eNOS to avoid vascular side effects, the study prioritized iNOS and eNOS as the most relevant NOS isoforms for antipsoriatic drug development. The results obtained were expressed as enzyme percent inhibition and are reported in Table 1.

**Table 1:** hiNOS and beNOS Inhibition by the Target Compounds^a^,^b^,^c^

	**inhibition (%)** ^d^	
**compound**	**hiNOS** ^d^	**beNOS** ^d^
**6**	87 ± 4	n.a.
**7**	95 ± 2	n.a.
**10**	79 ± 3	n.a.
**12**	51 ± 2	13 ± 0.4
**14**	83 ± 4	75 ± 3
**16**	45 ± 3	n.a.

a Results are expressed as enzyme percent inhibition.

b Values given are mean ± SD of three experiments.

c Evaluated in the presence of 1 μM concentration of each compound.

d Evaluated in the presence of 10 μM concentration of each compound. n.a.= not active (%inhibition <1).

All of the new molecules were able to inhibit iNOS, with enzyme percent inhibition ranging from 45% (**16**) to 95% (**7**). However, the indazole derivatives **12** and **14** inhibited also the eNOS; therefore, they were not considered for further evaluations. In contrast, compounds **6**, **7**, **10**, and **16** were selective over the constitutive isoform in the assayed conditions, and they were further evaluated to assess their IC_50_ values which are reported in Table 2, along with the relevant pIC_50_. While indazole-based compound **16** showed weak activity against human iNOS (IC_50_ = 1.38 μM), imidazopyridines **6** and **7** and quinoline derivative **10** were confirmed to be potent and selective hiNOS inhibitors. In particular, compound **7**, bearing a nitro-aminopyridine group, demonstrated higher potency (IC_50_ = 14 nM) compared to **6** (IC_50_ = 22 nM), which contains an aminopyridine. Both molecules were significantly more active than the reference compounds (BYK191023 IC_50_ = 82 nM, 1400W IC_50_ = 81 nM), pointing to the beneficial effect of the 2-aminopyridine moiety in the new inhibitors compared to the 4-methoxypyridine group in BYK191023. Although the quinoline–nitropyridine derivative **10** (IC_50_ = 82 nM) was less potent than compounds **6** and **7**, its activity was comparable to that of the reference. Therefore, **7** and **10**, each bearing nitropyridine groups, were selected to explore whether their chemical features might influence downstream biological responses.

**Table 2:** Inhibition of hiNOS and beNOS by Selected Target Compounds: IC_50_, pIC_50_, and Selectivity Evaluations

	**IC**_**50**_ **(nM)** ^a^			
**compound**	**hiNOS**	**beNOS**	**iNOS pIC**_**50**_	beNOS/hiNOS selectivity
**6**	22 ± 0.5	>10,000	7.66	>454
**7**	14 ± 0.8	>10,000	7.85	>714
**10**	82 ± 0.5	>10,000	7.09	>116
**16**	1380 ± 12	>10,000	<6.00	>10
**BYK191023**	82 ± 0.6	>10,000	7.09	>122
**1400W**	81 ± 0.2	>10,000	7.09	>123

a Values given are mean ± SD of experiments performed in triplicate at five different concentrations.

### Cell Metabolic Activity of HaCaT Cells under Basal and Inflamed Conditions in the Presence of Compounds

To demonstrate whether compounds **7** and **10** might be effective in psoriatic cells, their biological behavior was first investigated in an *in vitro* model of keratinocytes (HaCaT cells) stimulated with a combination of cytokines involved in the pathogenesis and maintenance of psoriasis.^33^

Under basal conditions, compounds **7** and **10** display good biocompatibility in all of the experimental conditions, both after 24 and 48 h (Figure 2A,B). As shown, cell metabolic activity is not affected by the exposure to compounds. As expected, when HaCaT cells are stimulated by the combination of cytokines, their metabolic activity dramatically decreases to almost 50% after 24 h and up to 40% after 48 h. While **7** weakly restores cell metabolic activity at 50 and 100 μM after 24 h and is found to be not efficient after 48 h (Figure 2A), compound **10**, at the same concentrations, slightly but significantly counteracts the effects of cytokines at both 24 and 48 h (Figure 2B). This effect could be related to the capability of compound **10** to decrease the NO in stimulated HaCaT cells under pro-inflammatory conditions after 24 h as reported in Figure S1.

**Figure 2:**
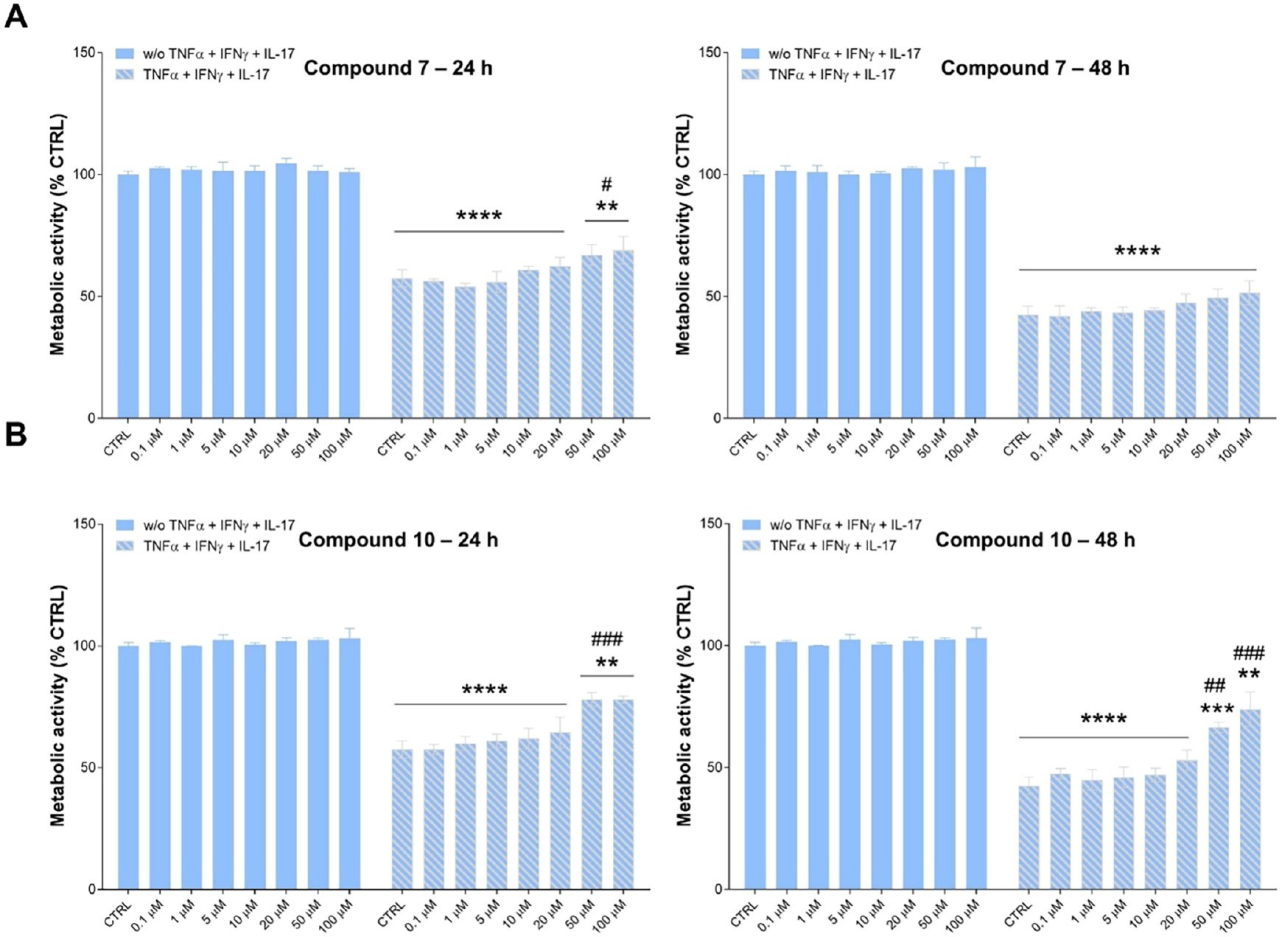
Effect of compounds **7** (A) and **10** (B) on the HaCaT cells’ metabolism under basal and pro-inflammatory conditions. Cell metabolic activity was measured through the MTT assay. The bar graphs represent percentages of cell metabolic activity after 24 and 48 h in the presence of compounds **7** and **10**. The untreated control (=CTRL) is set as 100%. **p < 0.01, ***p < 0.001, and ****p < 0.0001 between treatments and the untreated control; ## *p* < 0.01 and ### *p* < 0.001 between treatments and CTRL (cells stimulated with TNF-α, IFN-γ and IL-17).

### Evaluation of the Effects of iNOS Modulators on Keratinocyte Viability, Growth, and Apoptosis

In the next series of experiments, we evaluated whether the iNOS modulators **7** and **10** could influence the viability, growth, and apoptotic processes of human keratinocytes. All of the experiments were conducted using primary cultures of human keratinocytes isolated from skin biopsies of healthy donors.^34^ We first assessed the potential cytotoxic effects of iNOS chemical modulators and tested different concentrations of these compounds in keratinocyte cultures. Thus, primary cultures were treated with 1, 10, and 50 μM of compounds **7** and **10**, or with 1400W, a compound widely used in different *in vitro* pathology models as a pharmacological tool to selectively inhibit iNOS, including studies on inflamed keratinocytes.^35^ Cell viability, cell-cycle progression, and apoptotic rate were evaluated at 24, 48, and 72 h of treatment (Figure 3). The cytotoxicity of the compounds was evaluated by measuring LDH release in the culture supernatants, while cell-cycle progression and apoptotic rate were assessed by measuring the percentage of cells in G0-G1/S/G1-M phases and annexin V/propidium iodide staining positivity of keratinocytes, respectively. As shown in Figure 3A, both **7** and **10** compounds, at 1- and 10-μM doses, did not induce cytotoxicity in keratinocytes as they did not alter LDH production and release in the culture supernatants. In contrast, keratinocytes treated for 48 and 72 h with 50 μM **7** and **10** showed enhanced necrosis, as they showed higher LDH levels compared to cultures treated with the vehicle alone. Consistently, **7** and **10** administered at 50 μM concentration, induced a cell-cycle arrest in G0/G1 phase, and, in parallel, significantly decreased the percentage of keratinocytes in S and G2/M phases (Figure 3B). Viable keratinocytes were also reduced by 50 μM **7** or **10** treatments, as assessed by quantifying necrotic, early apoptotic and late apoptotic cells by flow cytometry analysis of treated cultures (Figure 3C). Importantly, **7** or **10** administered at **10** μM dose did not affect the cell-cycle progression nor the viability of cultured keratinocytes (Figure 3C). 1400W iNOS inhibitor did not influence viability, growth, or apoptosis rate of keratinocyte cultures at any of the tested doses.

**Figure 3:**
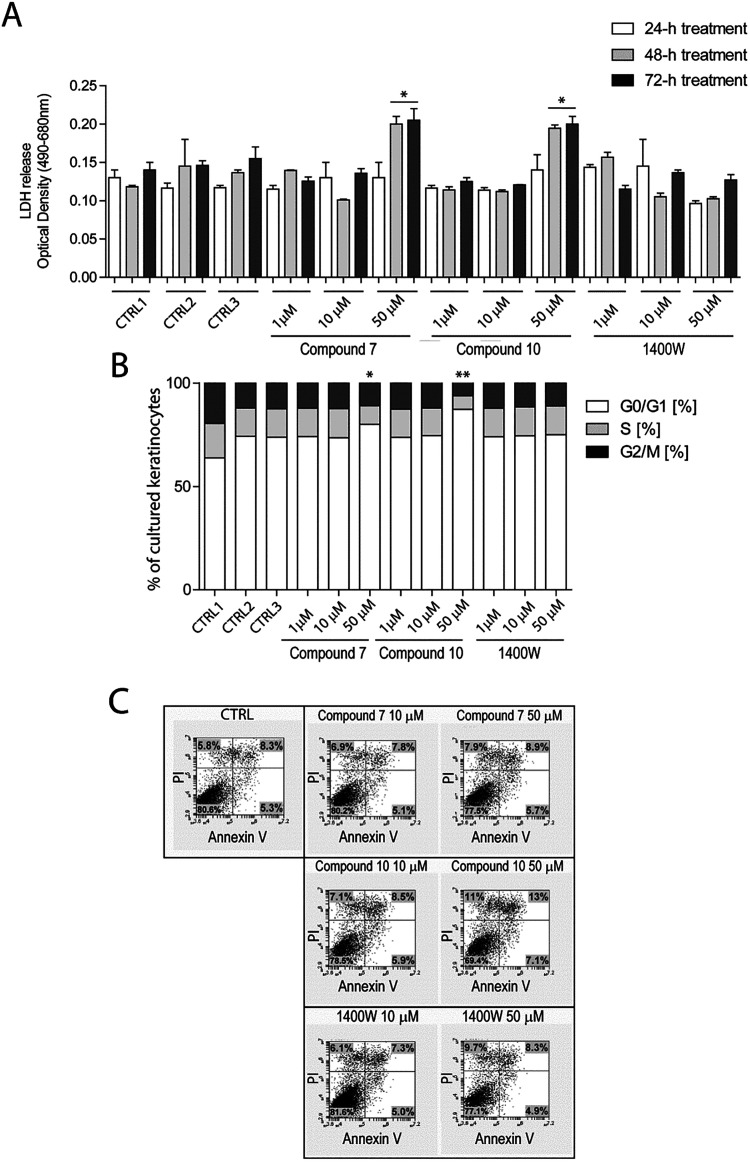
Evaluation of the effects of iNOS modulators on keratinocyte viability, growth, and apoptosis. (A) LDH release (measured as optical absorbance at 490 and 680 nm) in the supernatants of keratinocyte cultures undergoing treatments with 1–50 μM range doses of **7**, **10**, and 1400W iNOS inhibitors for 24, 48, and 72 h. CTRL1, CTRL2, and CTRL3 represent culture conditions with KBM, KGM, or KBM + 0.1% DMSO without iNOS inhibitors. Data are expressed as mean ± SD of three experiments performed on undiluted keratinocyte supernatants. *p < 0.05, compared to untreated keratinocyte cultures (CTRL1, CTRL2, and CTRL3) at 48 and 72 h. (B) Cell-cycle analysis of cultured keratinocytes treated or not (CTRL1, CTRL2, and CTRL3) with **7**, **10**, and 1400W compounds at 1, 10, and 50 μM for 24 h. Bars include three portions, each corresponding to the percentage of keratinocytes in G0-G1 (white), S (gray), and G2/M (black) phases. *p < 0.05 com*p*ared to untreated keratinocyte cultures (CTRL1, CTRL2, and CTRL3). (C) Apoptosis of cultured keratinocytes left untreated (CTRL) or treated with **7**, **10**, and 1400W at 10 and 50 μM doses for 24 h was examined by measuring annexin/PI (propidium iodide) fluorescence through flow cytometry analysis. In each dot plot, the percentages of PI^+^ (upper left), annexin V^+^ (lower right), PI/annexin V^+^ (upper right), or negative (lower left) cells are indicated. One out of three representative experiments are shown.

### Inhibition of iNOS Activity by **7** and **10** Compounds Downregulates Inflammatory Gene Expression in Experimental In Vitro and Ex Vivo Models of Skin Inflammation

Since iNOS is not constitutively expressed in human keratinocytes in homeostatic skin but is upregulated in diseased conditions, we next evaluated iNOS mRNA expression in keratinocyte cultures after activation with the cytokines IFN-γ, TNF-α, and IL-17A, which are abundantly produced in many skin dermatoses, including psoriasis,^36^ and are responsible for iNOS upregulation in resident skin cells and dendritic cells in these conditions.^37^ As expected, iNOS mRNA was not expressed by resting keratinocytes (Figure 4A). Following IFN-γ/TNF-α activation and even more potently after the addition of IL-17A, keratinocytes strongly expressed iNOS mRNA in a time-dependent manner, with a peak of mRNA expression at 18 h of stimulation (Figure 4A).

**Figure 4:**
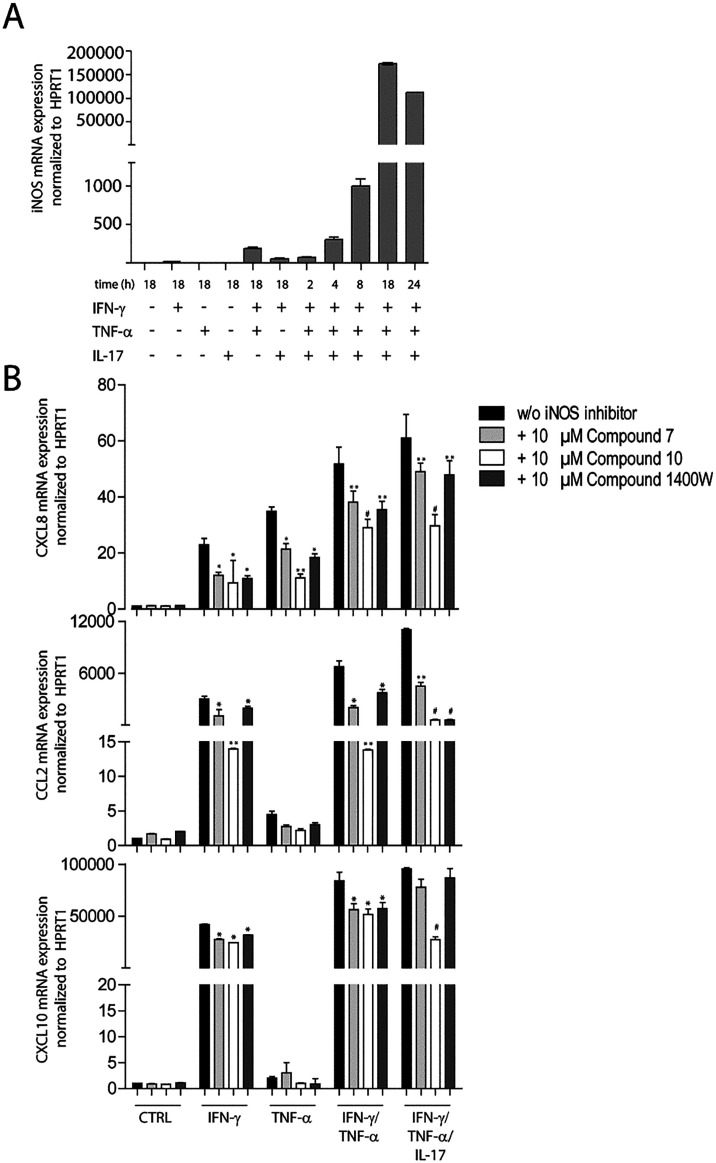
Induction of iNOS by IFN-γ, TNF-α, and IL-17 and modulation of inflammatory gene expression by iNOS chemical inhibitors in cultured keratinocytes. (A) iNOS expression was evaluated by real-time PCR analysis performed on RNA from keratinocyte cultures treated with rh IFN-γ, TNF-α, and IL-17A, administered alone or in combination for the indicated time periods. Data are expressed as the mean of three independent experiments ± SD. (B) The IFN-γ-, TNF-α-, IFN-γ/TNF-α-, and IFN-γ/TNF-α/IL-17-induced CXCL8, CCL2, and CXCL10 expression were evaluated by real-time PCR analysis of RNA from cultured keratinocytes stimulated with the specific cytokines in the presence or absence of 10 μM **7** (gray bars), **10** (white bars), or 1400W (dark gray) iNOS inhibitors, and normalized to HPRT1 mRNA. * *p* < 0.05, ** *p* < 0.01, and # *p* < 0.001, compared to keratinocyte cultures treated with IFN-γ, TNF-α, IFN-γ/TNF-α-, and IFN-γ/TNF-α/IL-17 (black bars).

In the next series of experiments, we evaluated whether iNOS chemical modulators could influence the expression of molecules induced by pro-inflammatory cytokines in human keratinocytes. To this end, the expression of a variety of molecules involved in the induction or control of skin inflammation was studied by real-time PCR analysis performed on RNA from keratinocyte cultures treated with rh IFN-γ, TNF-α, and IL-17A cytokines, coadministered with 10 μM **7**, **10**, or 1400W iNOS inhibitors. We found that **7** and 1400W, and more efficiently **10**, substantially reduced CXCL8, CCL2, and CXCL10 mRNA induced by IFN-γ alone or together with TNF-α, and by a mix of three cytokines IFN-γ, TNF-α, and IL-17, which potently activate inflammatory responses in keratinocytes (Figure 4B). In parallel, all iNOS modulators significantly reduced the membrane ICAM-1 and MHC class I and II molecules induced by IFN-γ and TNF-α in keratinocytes, as assessed by flow cytometry analysis (Figure 5A). In this case, **10** exhibited more prominent inhibitory effects compared to **7** and 1400W compounds. Since iNOS can potentially mediate apoptosis in keratinocytes via NO production and potentiate apoptosis by IFN-γ and TNF-α, we next evaluated whether iNOS modulators can revert the IFN-γ/TNF-α-mediated apoptotic processes in keratinocytes. To this end, we treated keratinocyte cultures with **7**, **10**, or control 1400W iNOS modulators, administered together with the pro-apoptotic and pro-necrotic stimuli IFN-γ and TNF-α, and quantified cell death by measuring annexin V/PI staining by flow cytometry. As shown in Figure 5B, 1400W compound and more efficiently **7** or **10** compounds, significantly reduced necrosis (PI^+^ cells), but not early apoptosis (Annexin V^+^) and late apoptosis (Annexin V/PI^+^) induced in keratinocytes following IFN-γ and TNF-α stimulation. In addition, viable cells enhanced when **7**, **10**, and 1400W compounds were coadministered with IFN-γ and TNF-α.

**Figure 5:**
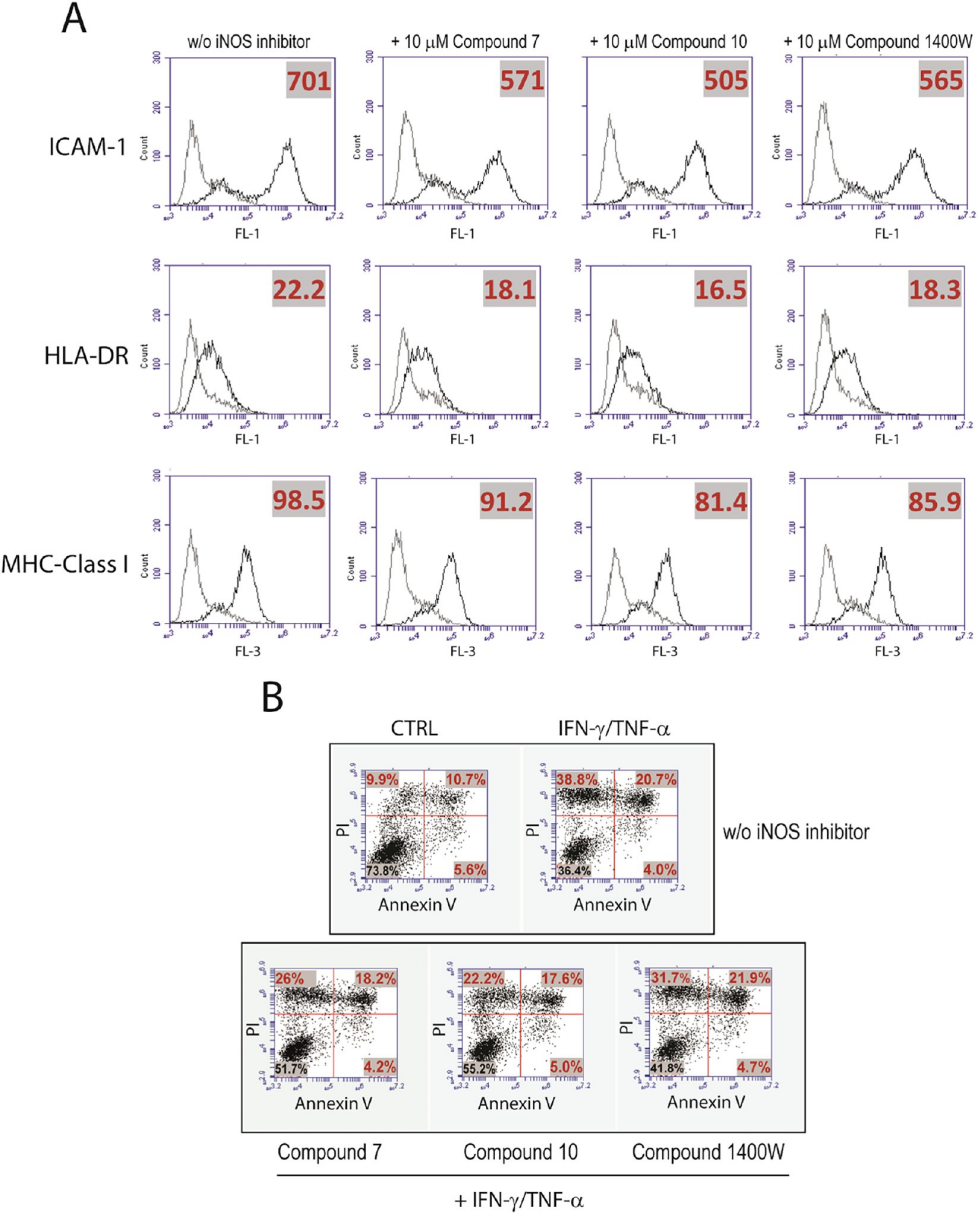
(A) Regulation of membrane molecule expression by iNOS inhibitor compounds in cultured keratinocytes. Cultures were analyzed for ICAM-1, HLA-DR, and MHC class I expression by flow cytometry after 48 h of treatment with IFN-γ and TNF-α, in the presence or absence of 10 μM **7**, **10**, and 1400W iNOS inhibitors. Thin lines represent staining with matched isotype Ig. The *x*-axis and the *y*-axis indicate the relative cell number and fluorescence intensity, respectively. One of three representative experiments is shown. (B) Apoptosis of cultured keratinocytes left untreated (CTRL) or treated with IFN-γ and TNF-α, in the presence or absence of 10 μM **7**, **10**, and 1400W iNOS inhibitors for 24 h, was examined by measuring Annexin/PI (propidium iodide) fluorescence through flow cytometry analysis. In each dot plot, the percentage of PI^+^ (upper left), annexin V^+^ (lower right), PI/annexin V^+^ (upper right), or negative (lower left) cells are indicated. One of three representative experiments is shown.

As a whole, our data indicate that iNOS inhibition, and likely NO level reduction, may be important for reducing cytokine-induced inflammatory responses and necrosis in keratinocytes.

Consistent with these findings, treatment with iNOS inhibitors did not alter the histological features of skin explants obtained from healthy subjects, as shown by H&E staining, even when combined with a cytokine mixture of IFN-γ, TNF-α, and IL-17 (Figure 6A). Both epidermal and dermal structures were perfectly maintained after a 3 h treatment of skin explants with compounds **7**, **10**, and 1400W in the presence of cytokines. As shown in Figure 6B, stimulation with IFN-γ, TNF-α, and IL-17 cytokines to mimic *in vivo* inflammatory conditions strongly upregulated mRNA expression levels of CXCL8, CXCL10, and CCL2 chemokines and ICAM-1, as well as of the iNOS enzyme. In line with *in vitro* findings, all mRNA levels were significantly decreased when skin explants were exposed to iNOS inhibitors, with the inhibitory effect seemingly being more pronounced with compound **10** (Figure 6B). In parallel, immunohistochemical analysis in *ex vivo* explants of phosphorylated STAT1 and STAT3, the two main transcriptional mediators of IFN-γ, revealed a slight reduction in their activation upon treatment with iNOS inhibitors, although this effect did not reach statistical significance (Supporting Figure S2).

**Figure 6:**
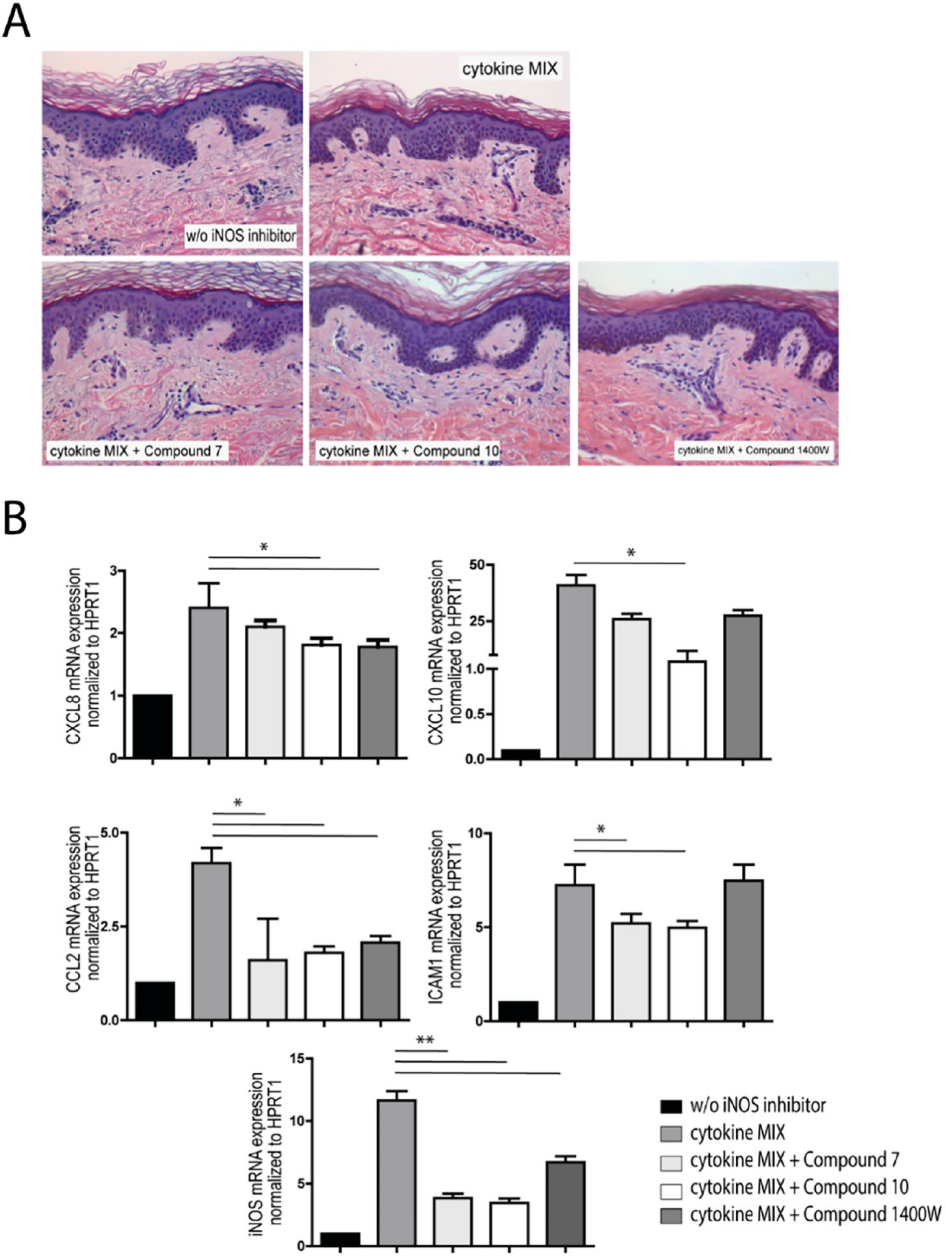
(A) iNOS inhibitors did not alter the histological features of skin explants obtained from healthy subjects, even when combined with a mixture of pro-inflammatory cytokines. H&E staining of paraffin-embedded sections of *ex vivo* skin grafts (*n* = 3), obtained from healthy human skin and cotreated for 3 h with a combination of IFN-γ, TNF-α, and IL-17A (cytokine MIX) together with compound **7**, compound **10**, 1400W (all at 20 μM), or vehicle alone (w/o iNOS inhibitor), is shown. Representative staining for each condition is shown. (B) mRNA expression levels of CXCL8, CXCL10, CCL2, ICAM-1, and iNOS induced in cultured keratinocytes by the cytokine MIX were reduced by compounds **7**, **10**, and 1400W. mRNA expression was measured in *ex vivo* skin explants by quantitative real-time PCR. Data were normalized to HPRT1 mRNA levels and presented as mean ± SD (*n* = 3 independent experiments). Statistical significance is indicated as *p < 0.05 and **p < 0.01.

Overall, our data indicates that iNOS inhibition, and likely NO level reduction, may be important for protecting skin cells against the pro-necrotic effects of cytokines and reducing inflammatory responses in both *in vitro* and *ex vivo* models of skin inflammation.

### Cell Metabolic Activity of Undifferentiated Monocytes and Macrophages under Basal and Pro-Inflammatory Conditions in the Presence of Compounds

Since high levels of monocytes and an increased number of macrophages are found in psoriatic lesions, we investigated the effects of **7** and **10** on these immune cells’ proliferation under basal and pro-inflammatory conditions. Undifferentiated monocytes and macrophages were exposed to **7** and **10** at increasing concentrations under basal conditions for up to 48 h to demonstrate the biocompatibility of compounds (Figure 7). Cell metabolic activity percentages are slightly perturbated compared to the one of untreated cells in the presence of compounds. Since the perturbation does not show dose dependence, it is plausible to assume that compounds show good tolerability when macrophages are not stimulated. When cells are stimulated by LPS to establish an inflamed environment and to upregulate the iNOS enzyme,^38^ their metabolic activity increases dramatically, independently from the cell type, as a sign of LPS-mediated immunostimulation. Compound **10** can restore the cell metabolic activity to the levels of control in LPS-stimulated monocytes (Figure 7A) in a dose-dependent manner. When administered on LPS-stimulated macrophages, **10** significantly decreased the cell metabolic activity mainly at the highest concentrations administered (50 and 100 μM). In parallel, compound **7** seems less effective than **10** and can decrease cell metabolic activity in LPS-stimulated monocytes, mainly in the highest concentration range (50 and 100 μM). When administered on LPS-stimulated macrophages, **7** is more effective from the concentration of 10 μM (Figure 7C).

**Figure 7:**
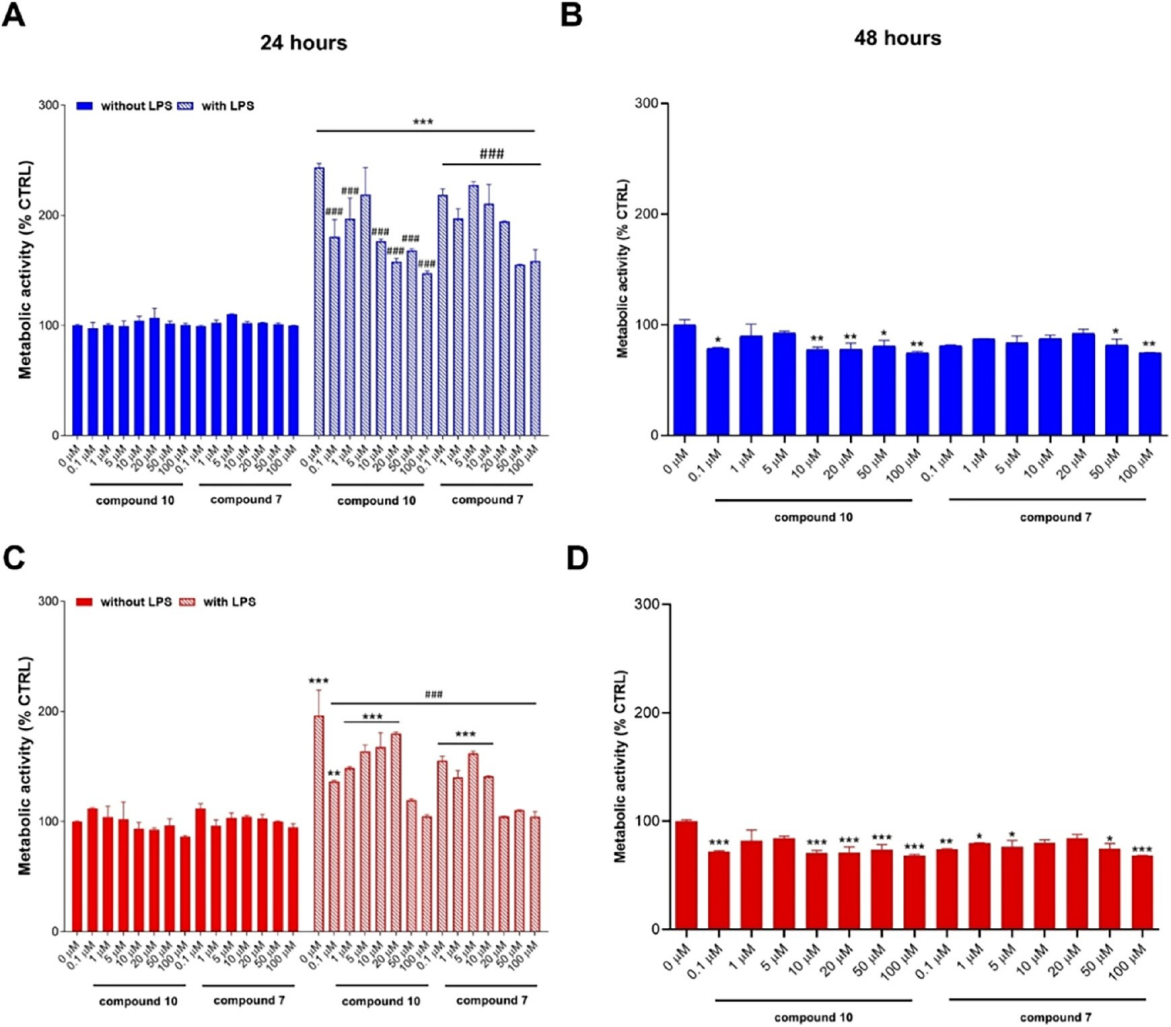
Cell metabolic activity of undifferentiated monocytes (A and B) and of macrophages (C and D) under basal (without LPS) and pro-inflammatory (with LPS) conditions after 24 h in the presence of **7** and **10**. (B and D) Cells exposed to compounds after 48 h under basal conditions (without LPS). The untreated control (0 μM without LPS) was set as 100%. Values are shown as mean ± standard deviations (*n* = 9). *p < 0.01, **p < 0.001, and ***p < 0.0001 versus 0 μM without LPS; ###*p* < 0.0001 versus LPS alone.

Based on these results, it can be inferred that compound **10** seems more efficient in decreasing the LPS-induced immune cell metabolic activity, already in the low concentration range.

### In Vitro Immunophenotype of Macrophages under Pro-Inflammatory Conditions in the Presence of **7** and **10**

Different studies have demonstrated that the pro-inflammatory M1 macrophage phenotype is dominant in the psoriatic lesions and that the “resolving” M2 one is downregulated with respect to the control skin samples from normal individuals.^39–41^ Since recently it has been reported that iNOS deficiency can inhibit M1 macrophage polarization and the consequent release of pro-inflammatory factors,^42^ here, we have hypothesized that **7** and **10** could affect the M1/M2 macrophages ratio. CD163 is a macrophage specific scavenger receptor for haptoglobin–hemoglobin complexes found on the cell membranes of M2 macrophages. Its expression is strongly induced by the anti-inflammatory cytokine IL-10, making CD163 a marker of anti-inflammatory process occurrence. On the contrary, CD80+ cells are classically M1 macrophages.^43^ Therefore, the immunophenotypic profile of LPS-stimulated macrophages afterward exposed to compounds was evaluated (Figure 8). As expected, an LPS stimulation upregulated CD14, CD80, and CD163 on the membrane of inflamed macrophages. Expression levels of CD14 are slightly but significantly downregulated by both compounds independently from their concentrations. In parallel, **10** can decrease CD80 expression and increase CD163 in a dose-dependent manner. The differential dose-dependent effect of compound **10** on CD163 versus CD14 and CD80 likely reflects the compound’s selective modulation of anti-inflammatory signaling pathways, and future investigation into the signaling pathways and cellular responses involved may help clarify this nonlinear behavior. In contrast, compound **7** seems ineffective on the modulation of CD80 and CD163 expressions (Figure 8A). Interleukin-6 (IL-6) has a broad effect on cells of the immune system and those not of the immune system and often display hormone-like characteristics that affect homeostatic processes. IL-6 has context-dependent pro- and anti-inflammatory properties and is now regarded as a prominent target for clinical intervention.^44^ It has been widely reported that NO activates IL-6 production *in vivo* under pro-inflammatory conditions in skin.^45^ In our experimental model, the secretion of IL-6 from LPS-stimulated macrophages is dramatically increased 24 h after stimulation (Figure 9). Both compounds decrease IL-6 amounts, independently of their concentrations.

**Figure 8:**
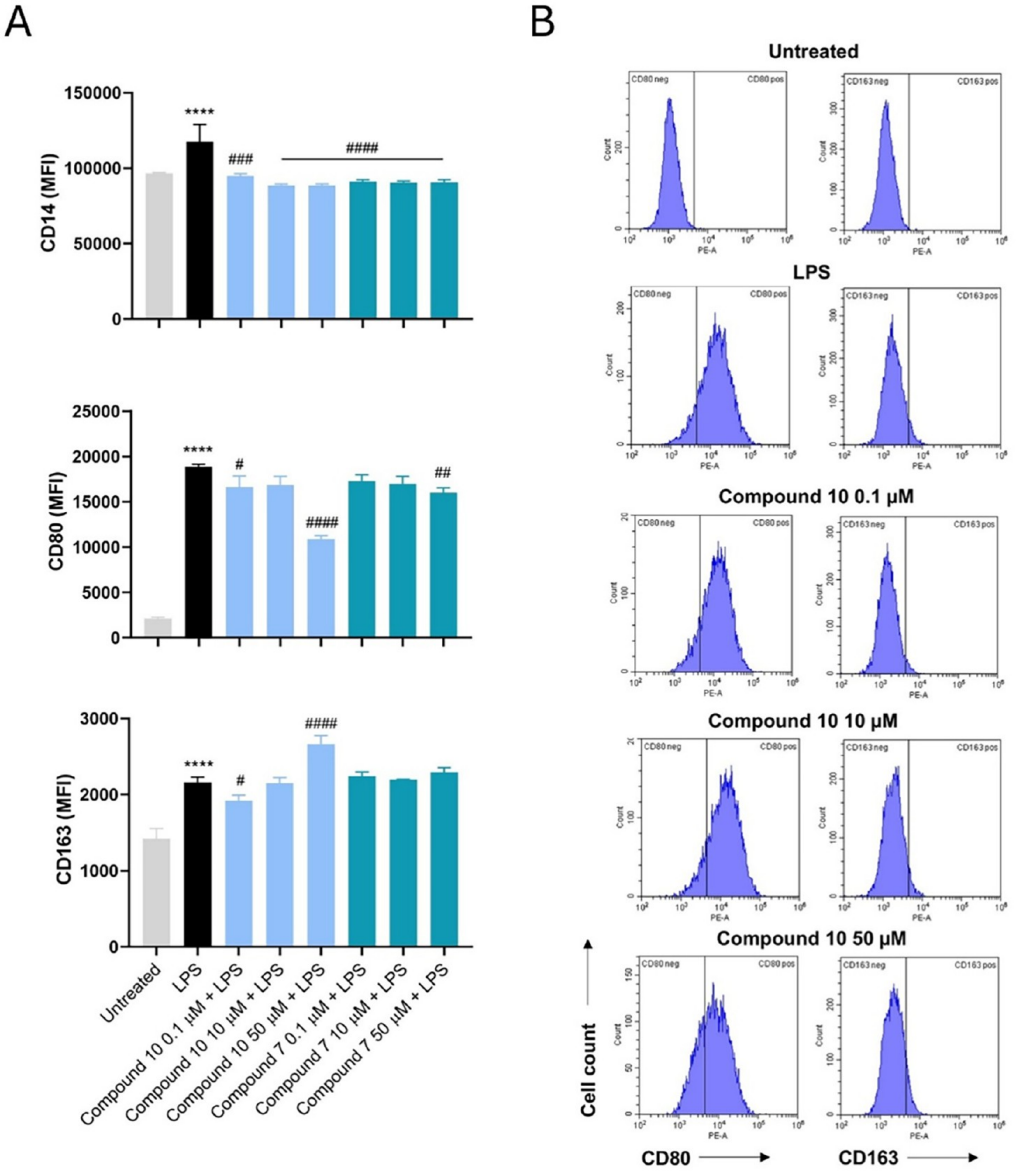
Immunophenotypic profile of LPS-stimulated macrophages in the presence of compounds **7** and **10**. (A) Bar graphs represent fluorescence emissions (MFI = mean fluorescence intensity) related to CD14, CD80, and CD163. (B) Peaks of emission obtained by flow cytometry. ****p < 0.0001 versus untreated; #*p* < 0.01, ###*p* < 0.001, and ####*p* < 0.0001 versus LPS alone.

**Figure 9:**
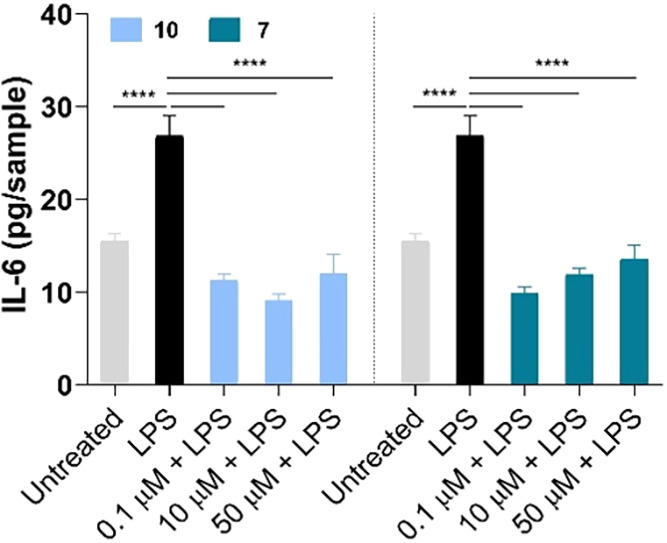
IL-6 secretion from LPS-stimulated macrophages in the presence of selected concentrations of **7** and **10**. Amounts of IL-6 in cell supernatants were normalized on 3-(4,5-dimethylthiazol-2-yl)-2,5-diphenyltetrazolium bromide (MTT) data (*n* = 4). Untreated: cells exposed to growth medium only. LPS: cells stimulated with LPS only. ****p < 0.0001.

From all the collected results, it emerged that compound **10** was more effective with respect to **7** and 1400W in reducing cytokine-induced inflammation and cell necrosis, also shifting macrophages from a pro-inflammatory to a resolving phenotype. Therefore, **10** was selected for further *in vitro* and *in vivo* evaluations.

### Microsomal Stability Evaluation

Compound **10′s** metabolic stability was evaluated in liver microsomes according to a literature method.^46^ Based on the data obtained, the compound shows moderate metabolic stability, with a *t*_1/2_ of 28.5 min and an intermediate *in vivo* intrinsic (hepatic) clearance (CLint) of 10.8 mL min^–1^ kg^1–^. These preliminary data warrant further early preclinical investigations of **10**, although studies on skin-specific stability and penetration are necessary to confirm its suitability for topical use.

### Toxicity Evaluation of Compound **10** in the Zebrafish Model

Considering the promising results obtained with compound **10**, *in vivo* toxicity tests were performed using zebrafish (Danio rerio) embryos and larvae. Zebrafish are increasingly recognized as a valuable and versatile alternative model in drug discovery research, offering several advantages such as ethical acceptability and providing a whole-organism context that bridges the gap between *in vitro* and mammalian models.^47–49^ Their benefits include small size, external development, transparency (which facilitates the assessment of phenotypic abnormalities), rapid development, and a high degree of genetic similarity to humans. In this contest, besides the ortholog of the mammalian NOS1 gene, zebrafish genome encodes two nos2 genes (nos2a and nos2b), which exhibit high homology with mammalian inducible NOS2.^50^ In zebrafish embryos, nos2b is constitutively expressed starting from 6 h post fertilization (hpf),^51^ while nos2a remains low during early development and increases at 96 hpf.^49^ Moreover, it has been demonstrated that both nos2 isoforms can be induced by pro-inflammatory or mechanical stress, such as tail transection.^50^ Based on the results obtained, compound **10** appears to be well tolerated in larvae exposed during the 48–120 hpf window at concentration of 10 and 20 μM (Figure 10A), as the survival is 100% and malformation rates are very low and not statistically different from the controls. The sporadic embryonic defects include pericardial edema, yolk deformation, and developmental delay, evident by a shortened head-to-tail length and uninflated swim bladder (Figure 10C). Conversely, when exposure begins shortly after fertilization (within 3 hpf), compound **10** exhibits toxicity in embryos as early as 24 hpf (data not shown), starting from a concentration of 10 μM, with a significant mortality rate and sublethal effects (Figure 10B).

**Figure 10:**
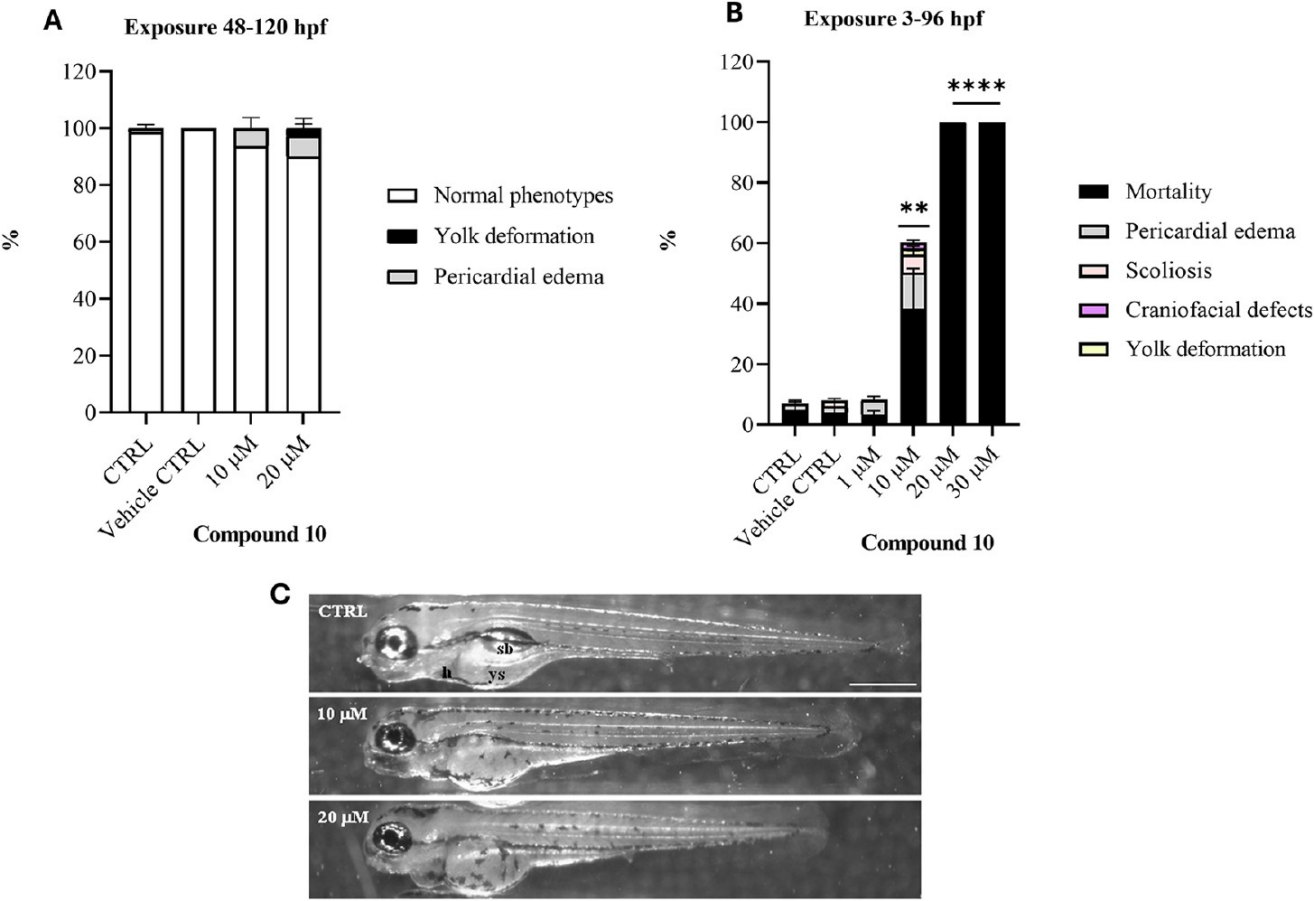
*In vivo* toxicity assay of compound **10** in zebrafish. (A) Survival and malformation rates in larvae at 120 h post fertilization (hpf) following 72 h of exposure starting from 48 hpf. (B) Mortality and malformation rates in 96 hpf larvae exposed to compound **10** starting from 3 hpf. (C) Lateral stereomicroscopic view of control (CTRL) and treated 120 hpf larvae during the 48–120 hpf window. The images show the sporadic phenotypic abnormalities observed in larvae exposed to concentrations of **10** and 20 μM. **p < 0.001, ****p < 0.00001 versus CTRL (*n* = 3, 20 embryos for each treatment). Scale bar 500 μm. Abbreviations: h: heart; ys: yolk sac; sb: swim bladder.

The toxicity of compound **10** during early development underscores the relevance of orthologous forms of the inducible mammalian NOS2 gene in regulating developmental processes such as cellular differentiation, proliferation, and organogenesis in zebrafish embryos. When these developmental processes are nearly complete, compound **10** is found to be nontoxic at the investigated concentrations. Considering the high translational value of zebrafish embryos in terms of their developmental pathways similar to those of mammals, these results suggest that further evaluation of this compound in mammalian models of teratogenicity should be performed in the next stages of preclinical development.

## Docking Studies

### Docking Analysis

A docking study was performed on compounds **7** and **10** to shed light on their binding modes into the iNOS and eNOS, and the results were compared to those of BYK191023. The last is a potent and selective hiNOS inhibitor, and a crystal structure of this inhibitor with murine iNOS has been described.^52,53^ Docking protocol was evaluated by cross-docking BYK191023 into hiNOS (PDB entry 4CX7) and comparing the predicted pose with the cocrystallized conformation observed in the reported murine iNOS complex (PDB entry 3NW2).

The docking pose of BYK191023 on hiNOS (PDB ID: 4CX7) suggested an overall binding placement consistent with key features of the crystallographic pose observed with murine iNOS (PDB ID: 3NW2) (Figure 11a). In this pose, the 4-methoxypyridine ring is displayed in the guanidinium binging site of natural substrate l-arginine, π-stacking the heme cofactor and inserting the methoxy group into a hydrophobic pocket set by Val532 and Phe369. In the docking pose, the N atom of the pyridine ring is h-bonding Glu377, as seen in the crystal structure (PDB ID: 3NW2). As for the imidazo­[4,5-*b*]­pyridine ring, in the docking pose this moiety is displayed toward the entrance of the catalytic site and into a pocket set by residues Trp346, Arg388, Arg266, Gln263, and Tyr373, forming an h-bond with the later residue.^54^ This direct h-bond interaction is not seen in the crystal structure but is established through an intermediate water molecule (shown in Figure 11 for comparison). Notably, this water molecule is not present in the hiNOS structure used for docking (PDB ID: 4CX7) and therefore was not included explicitly in the docking calculations, which may account for the observed local difference in this region. Moreover, in the crystallographic complex (PDB ID: 3NW2), this area is located close to residue Gln257, which could contribute to shaping and stabilizing the local binding environment around the imidazo­[4,5-*b*]­pyridine region. As for ligand **7**, its docking pose on hiNOS (PDB ID 4CX7) matched that of inhibitor BYK191023 with an identical disposition of the 4-nitropyridine and imidazo­[4,5-*b*]­pyridine rings (Figure 11b). The main difference, however, lies in the h-bond interactions established by **7** with Glu377; in this case, two h-bonds are found, one with the 2-amino group of the pyridine and the other with N3 at the imidazo­[4,5-*b*]­pyridine moiety. Moreover, the imidazo­[4,5-*b*]­pyridine moiety is able to form a third h-bond between 4-N and Tyr373 as seen in BYK191023.

**Figure 11:**
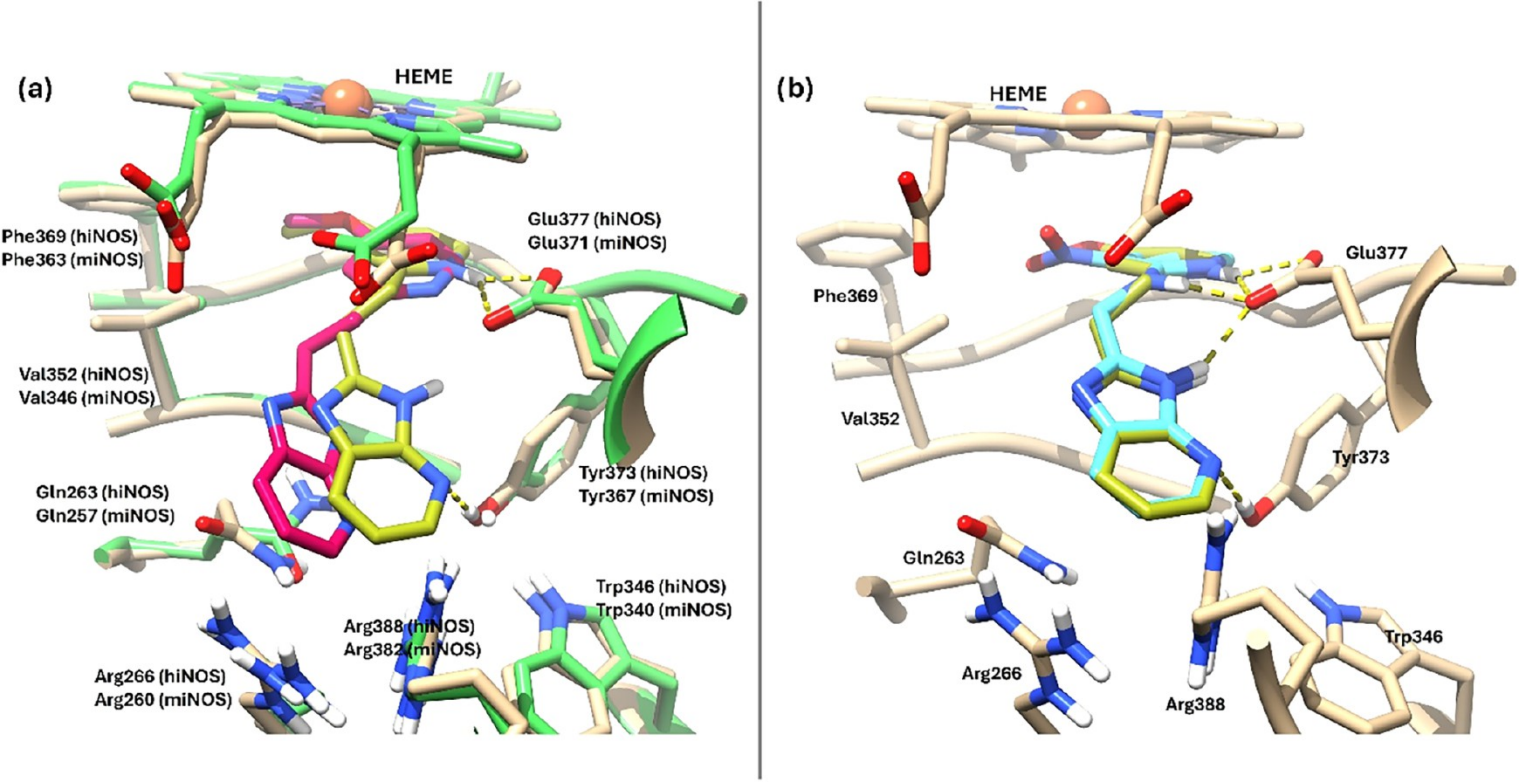
(a) Superimposed predicted binding poses of BYK191023 (light olive green) on human iNOS (pdb ID 4CX7, tan); cognate BYK191023 (dark pink) on murine iNOS (pdb ID 3NW2, lime). (b) Superimposed predicted binding poses of BYK191023 (light olive green) and **7** (cyan) on human iNOS (pdb ID 4CX7, tan). Hydrogen bonds are represented by dashed yellow lines.

Interestingly, while the crystal structure features a water-mediated contact with the heme propionate, our standard rigid docking, performed without explicit solvent to allow unbiased ligand placement, predicts a direct hydrogen bond instead. We interpret this slight translational shift of the imidazo­[4,5-*b*]­pyridine ring simply as the model’s geometric adaptation to the nonhydrated pocket, rather than a definitive mechanistic claim. This observation naturally motivates the subsequent explicit-solvent MD simulations for lead compound **10**, allowing the system to recover and validate the true dynamic hydration network (discussed below).

Subsequently, a molecular docking study of compound **7** was carried out on the bovine eNOS (beNOS) isoform and of compound **10** on the human iNOS (hiNOS) and bovine eNOS (beNOS) isoforms, both used in *in vitro* inhibition studies.

As for the docking pose of ligand **10** in the hiNOS isoenzyme (PDB 4CX7), this shows a similar disposition of its 3-nitropyridine ring underneath the heme cofactor, although in an orientation that diminishes the stacking interaction with the cofactor (Figure 12). This ring orientation, however, allows an H-bond of the nitro group with the backbone chain of Val352 at the hydrophobic pocket of the catalytic site and still retains an h-bond with Glu377 through its 2-amino group. The main difference, however, lies in the orientation of the 3-aminoquinoline ring. Likely due to its larger size, the ring does not fit into the pocket set by residues Trp346, Arg388, Arg266, Gln263, and Tyr373 at the entrance of the catalytic site, lacking the h-bond with Tyr373 and the π-stacking interaction with residue Gln263. This orientation, however, allows the h-bonding interaction of the 2-amino group with the propionate A group of the heme cofactor.

**Figure 12:**
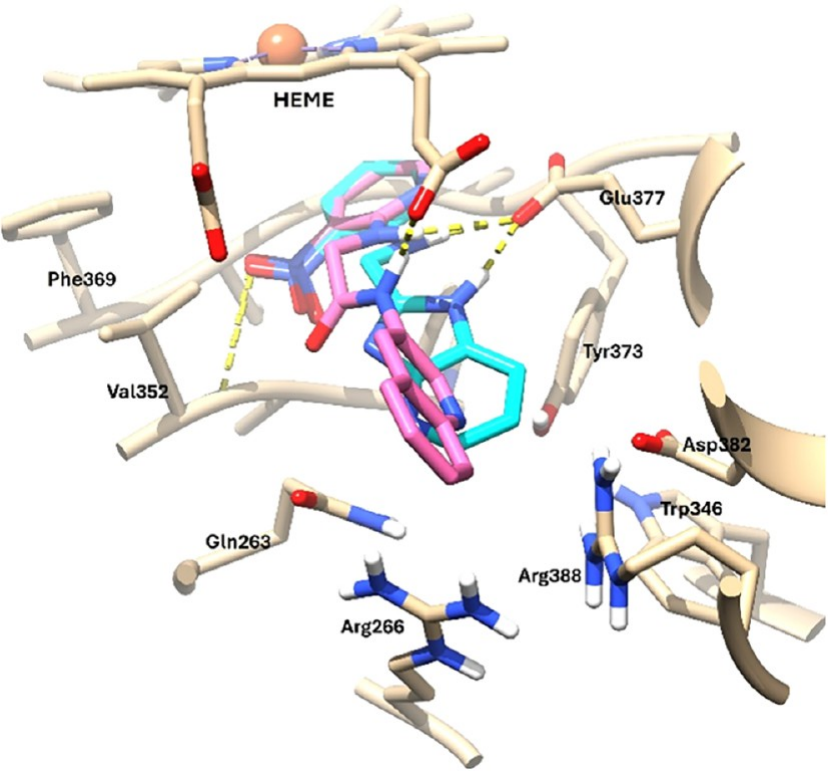
Superimposed predicted binding poses of **7** (light blue) and **10** (dark pink) on human iNOS (PDB entry 4CX7, tan). Hydrogen bonds are represented by dashed yellow lines.

Ligands **7** and **10** were also docked into the beNOS isoform (PDB entry 3E7S). In agreement with the conserved NOS pharmacophore, the top-ranked poses successfully maintained the 2-aminopyridine anchor positioned beneath the heme. Compound **7** shows a different binding pose on beNOS when compared to that of hiNOS. While the 2-aminopyridine ring remains beneath the heme group, in the case of the docking pose in beNOS this is slightly tilted, forming an angle with the heme ring and losing the capacity to form the h-bond between the 2-amino group and residue Glu360 (beNOS numbering). The greatest difference, however, comes from the arrangement of the imidazo­[4,5-*b*]­pyridine bicycle, which in the beNOS isoenzyme is disposed toward the heme group, inserting itself between the two carboxylate residues and establishing a h-bond with the propionate A residue. This is due to a closure of the iNOS specific pocket set by Tyr330, Arg249, Arg371, Gln246, and Tyr356, but also to the upward movement of Trp446 (beNOS numbering) observed in this specific crystal structure (3E7S), and to the presence of the beNOS specific residues: Val103 and Leu104, instead of Met120 and Thr121 present in hiNOS. Although these residues are not part of the catalytic site, they participate indirectly in its conformation from a second level. Thus, the change of Met120 (hiNOS) for Val104 (beNOS) allows the generation of a space in that position, which facilitates the disposition of the ligand in that zone (Figure 13a,b).

**Figure 13:**
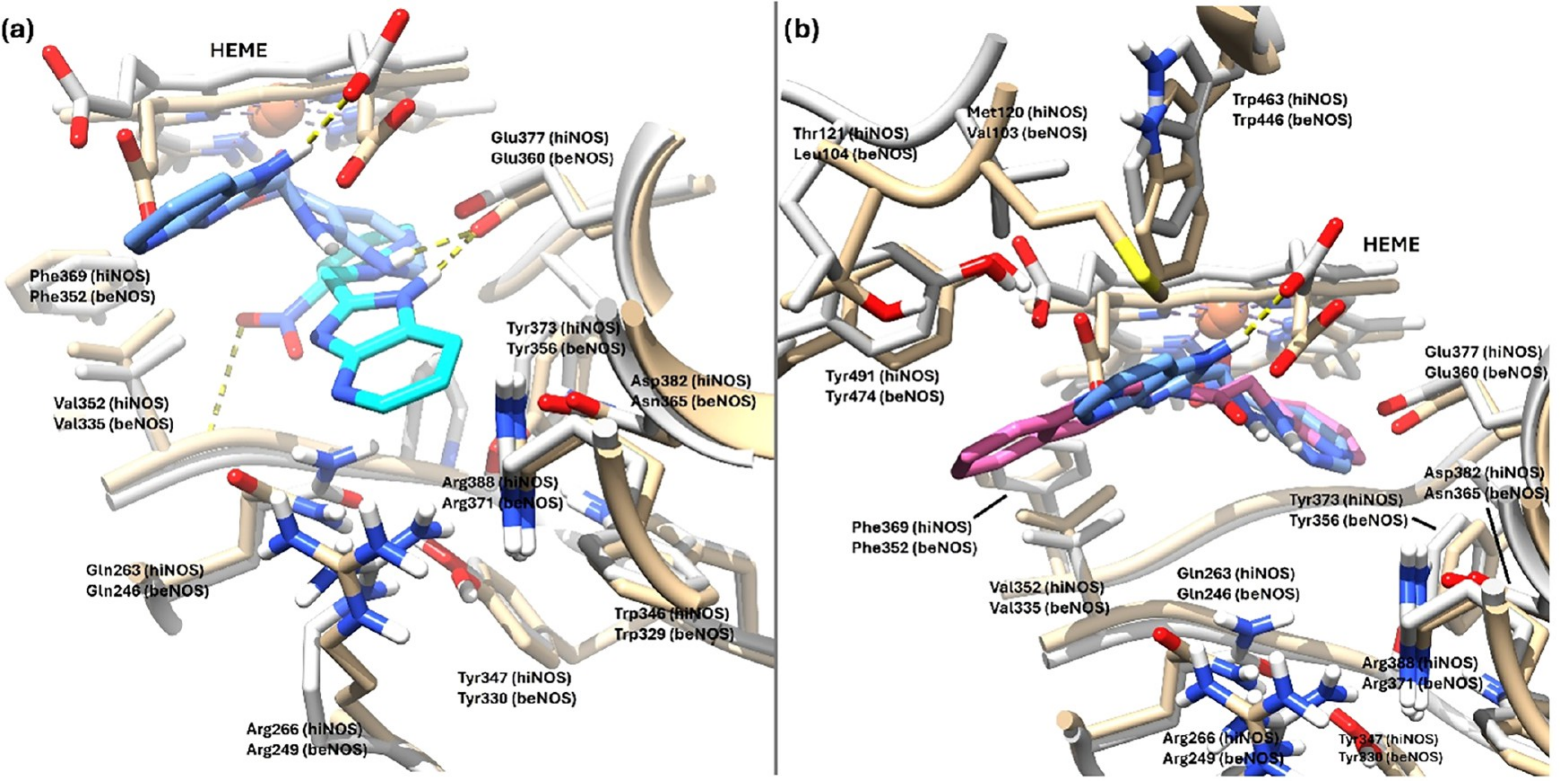
(a) Superimposed predicted binding poses of **7** (cyan) on human iNOS (PDB 4CX7, tan) and **7** (light blue) on bovine eNOS (PDB 3E7S, gray). (b) Predicted binding poses of **7** (light blue) and **10** (pink) on bovine eNOS (PDB entry 3E7S, gray); human iNOS isozyme (PDB entry 4CX7, tan) is superimposed for comparison. Hydrogen bonds are represented by dashed yellow lines.

Regarding ligand **10**, its binding pose on the bovine eNOS isoform (beNOS) (PDB entry 3E7S) is arranged similarly to that observed with **7** on this same isoenzyme; that is, the 2-aminopyridine ring is positioned beneath the heme group forming an angle with it, and the 3-aminoquinoline ring is oriented toward the catalytic site entrance and away from the natural substrate l-arginine binding zone. In addition, contributing to the binding pose of ligand **10** in beNOS is the phenomenon previously described for ligand **7**, which in the case of **10** is more pronounced given the larger size of its aromatic 3-aminoquinoline ring, which is stabilized by a π-interaction between this bicycle and the Tyr474 residue (Figure 13b). These novel poses observed in beNOS, which displace the ligands away from the natural substrate (l-arginine) binding site, could explain the low selectivity of these two compounds for the eNOS isoform.

## Molecular Dynamic Studies

To evaluate binding-mode stability and capture solvent-mediated effects not accessible to rigid docking, explicit-solvent molecular dynamics simulations were performed for compound **10** bound to hiNOS (4CX7) and beNOS (3E7S) in their biologically relevant homodimeric states. Each system was simulated in three independent replicas (50 ns), and monomers A and B were analyzed separately to capture subunit-dependent behavior (see the Supporting Information for the complete MD analysis).

Protein backbone RMSD analysis indicated stable dimeric scaffolds over the production window (Figure S1, Supporting Information), whereas ligand RMSD reveals pronounced isoform- and monomer-dependent heterogeneity (Figure 14). In beNOS monomer A, the ligand undergoes an early adjustment and then remains in a moderate RMSD regime consistent with a pose-like bound ensemble; beNOS monomer B samples higher RMSD values indicative of broader reorientation within the pocket. In hiNOS, ligand RMSD reaches higher values and shows step-like transitions, supporting sampling between recurrent bound arrangements rather than retention of a single rigid docking pose. These data support stable association of compound **10** with both enzymes over the simulated window while highlighting that the bound ensemble depends on both the isoform context and monomeric environment within the dimer.

**Figure 14:**
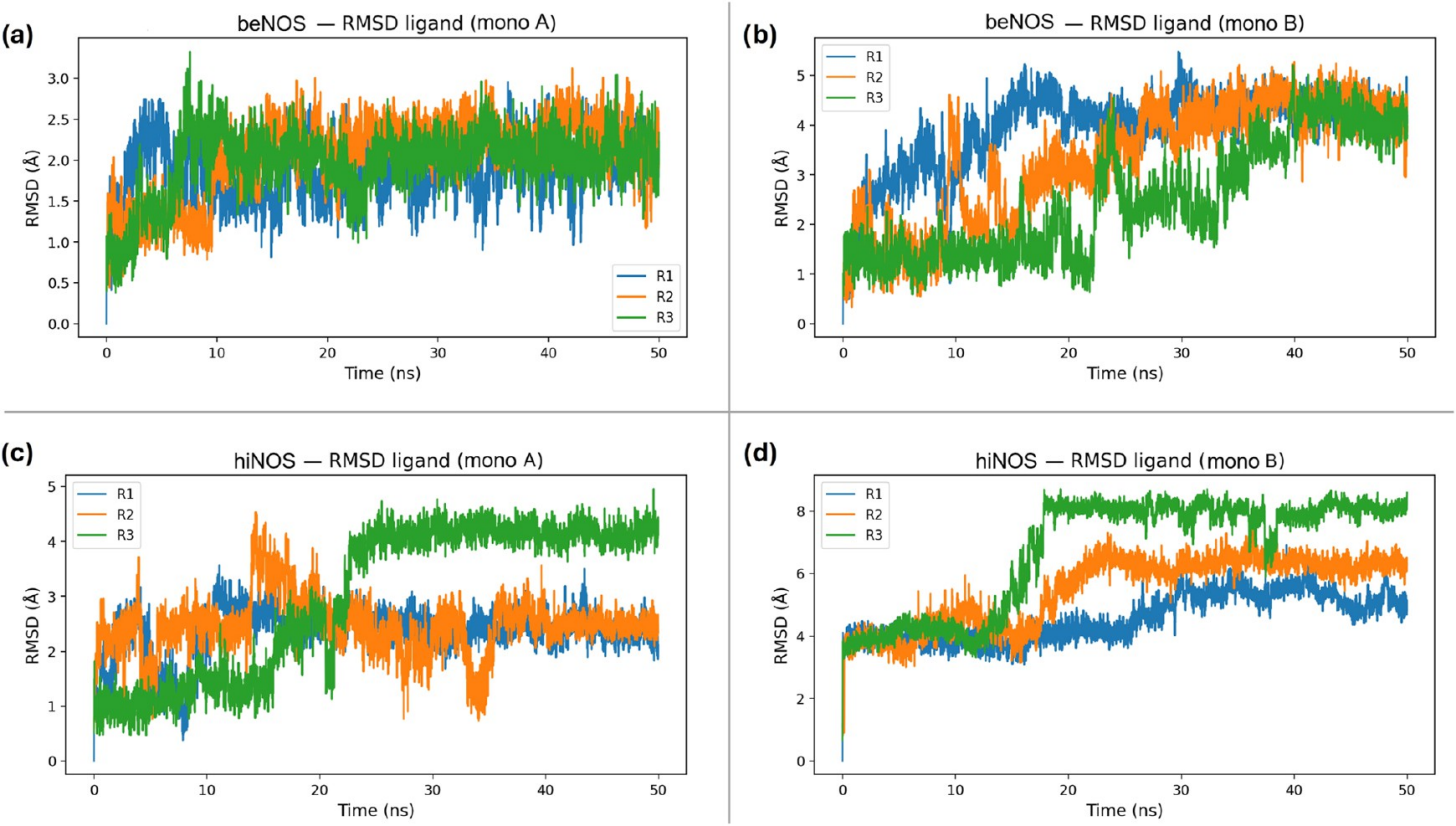
Ligand RMSD for compound **10** relative to the initial bound pose in each monomer. RMSD was computed for ligand heavy atoms after fitting each frame to the backbone of the corresponding monomer: (a) beNOS (PDB 3E7S) monomer A; (b) beNOS (PDB 3E7S) monomer B; (c) hiNOS (PDB 4CX7) monomer A; (d) hiNOS (PDB 4CX7) monomer B.

Building on the earlier RMSD analysis (which reflects overall structural stability and convergence), the RMSF analysis quantifies per-residue backbone fluctuations around the mean structure after least-squares superposition to a common reference. For clarity, RMSF is reported separately for monomers A and B so the two isoforms can be compared on equal footing. In both isoforms, the oxygenase domain remains largely rigid, with noticeable flexibility mainly at the termini and in a few small regions, consistent with a similarly stable structural framework. Differences between isoforms are not widespread; instead, they concentrate in specific regions close to the binding pocket (Figures 15 and S6), particularly around equivalent positions ∼44–55, ∼265–299, and ∼383–385 in monomer A. Importantly, these regions overlap with residues that consistently contact compound **10** or form hydrogen bonds in an isoform-dependent manner. Monomer B shows the same pattern, with the same pocket-adjacent segments accounting for the largest ΔRMSF and the strongest interaction enrichment.

**Figure 15:**
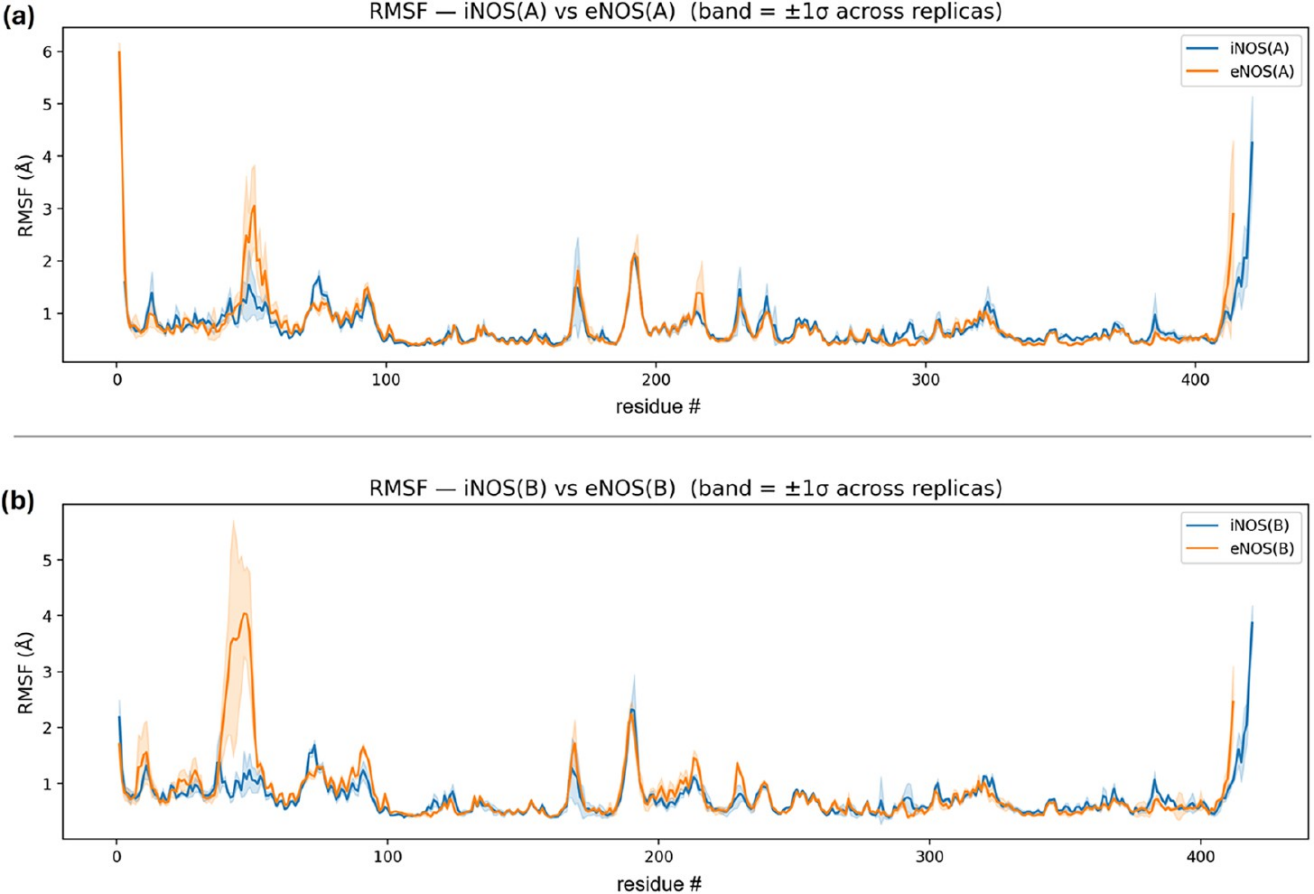
Backbone RMSF profiles (mean ± SD across replicas) for isoform comparisons. (a) hiNOS­(A) vs beNOS­(A). (b) hiNOS­(B) vs beNOS­(B). RMSF was computed after structural alignment and is plotted as a function of equivalent residue position; the solid line shows the replica-averaged RMSF and the shaded band indicates ± SD across R1–R3.

Following the preceding RMSF analysis, radius of gyration (Rg) and solvent-accessible surface area (SASA) studies were carried out as complementary global readouts of compactness and ligand exposure during the 50 ns production simulations (three replicas, R1 and R3; mean ± SD with 0.05 ns binning).

For the protein, Rg remains remarkably stable for both isoforms after the initial equilibration period, supporting preservation of the overall globular architecture across replicas. In beNOS, Rg rises rapidly from ∼29.2–29.3 Å to ∼29.5–29.6 Å within the first few nanoseconds and then fluctuates around a narrow plateau without persistent drift. In hiNOS, Rg is consistently higher (centered around ∼30.2–30.3 Å), shows a comparable early rise, and subsequently displays bounded fluctuations with modest replica dispersion; the limited variance (<∼0.3 Å) indicates no major expansion/compaction transitions on this time scale. In contrast, ligand Rg shows that compound **10** interconverts between more compact and more extended conformations (∼4.2–5.2 Å), with the strongest replica heterogeneity in hiNOS monomer B (Figures S7 and S8). SASA analyses further support global stability: both dimers show an initial relaxation followed by a stable regime without sustained drift, with hiNOS showing a short-lived compaction event around ∼8–10 ns. Ligand SASA reveals clear monomer asymmetry, with progressive burial in beNOS monomer B but increasing exposure in beNOS monomer A, while hiNOS monomer B displays marked replica heterogeneity and hiNOS monomer A remains steadier (Figures S9 and S10).

Binding-mode diversity was next characterized by clustering ligand–pocket configurations using a conservative two-state partition (*K* = 2) to capture the two most recurrent pose families in a directly comparable manner across isoforms and monomers. Representative cluster structures show isoform-dependent divergence in dominant bound arrangements (Figures 16 and 17). In hiNOS, compound **10** preferentially occupies the substrate-associated region and positions polar groups toward key catalytic residues (reported in the MD analysis with ensemble-to-PDB mappings, e.g., Glu295 ≈ Glu377 in PDB 4CX7 numbering, see SI for an extended discussion), while the 3-aminoquinoline system packs into a pocket near the H4B cofactor and surrounding residues. In contrast, beNOS representatives show that the ligand shifted away from the canonical l-arginine region toward an opposing, more hydrophobic pocket, where stabilization is dominated by hydrophobic contacts. This structural divergence provides a coherent mechanistic rationale for isoform-dependent behavior and emphasizes that selectivity-relevant features may arise from differences in pocket adaptation and residue mobility rather than from a single static docked geometry.

**Figure 16:**
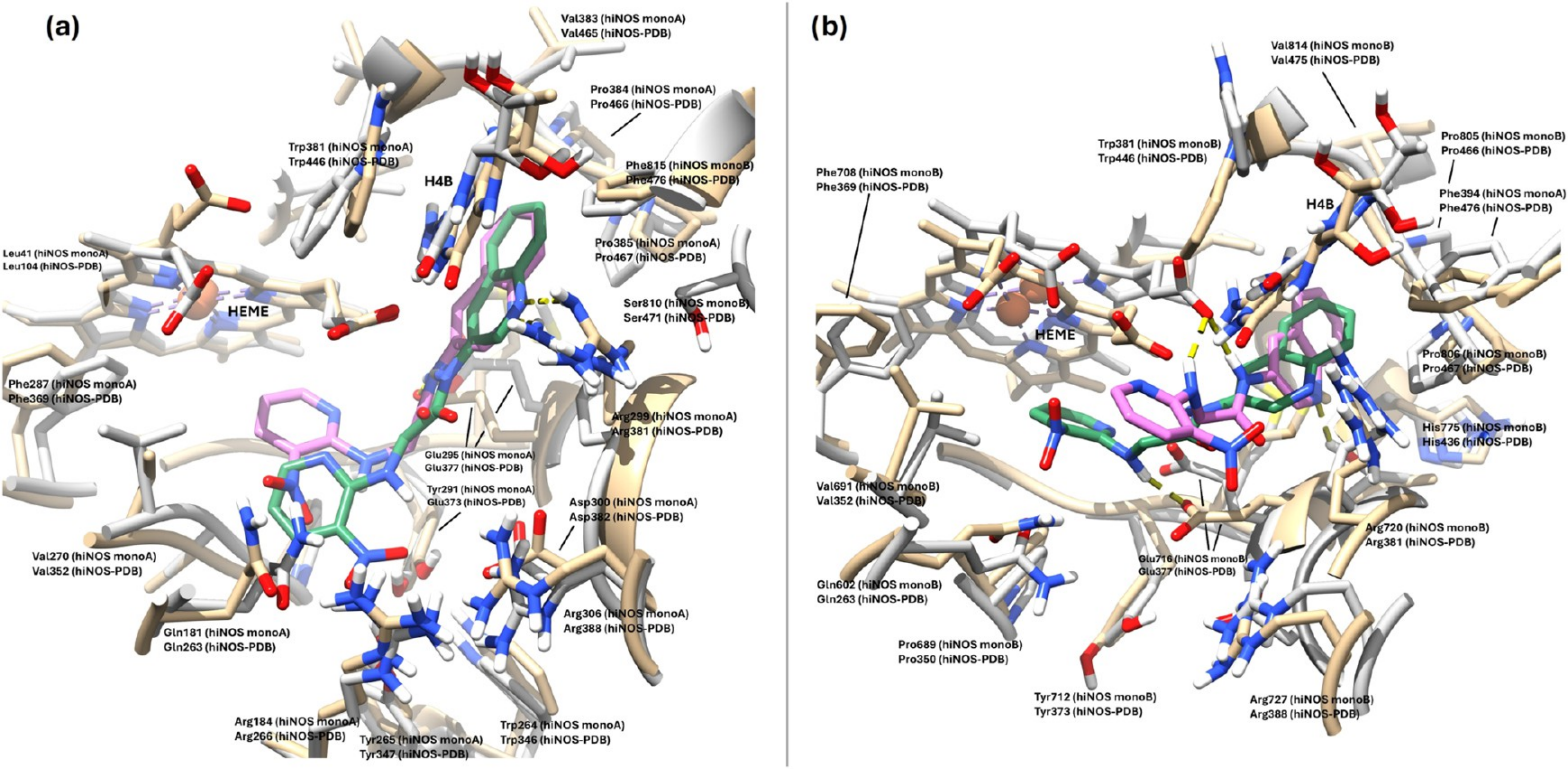
Representative cluster structures for hiNOS (PDB 4CX7) obtained from *K* = 2 clustering: (a) monomer A and (b) monomer B. In each panel, the protein is shown in tan with compound **10** in green for cluster 0, and protein in gray with compound **10** in magenta for cluster 1.

**Figure 17:**
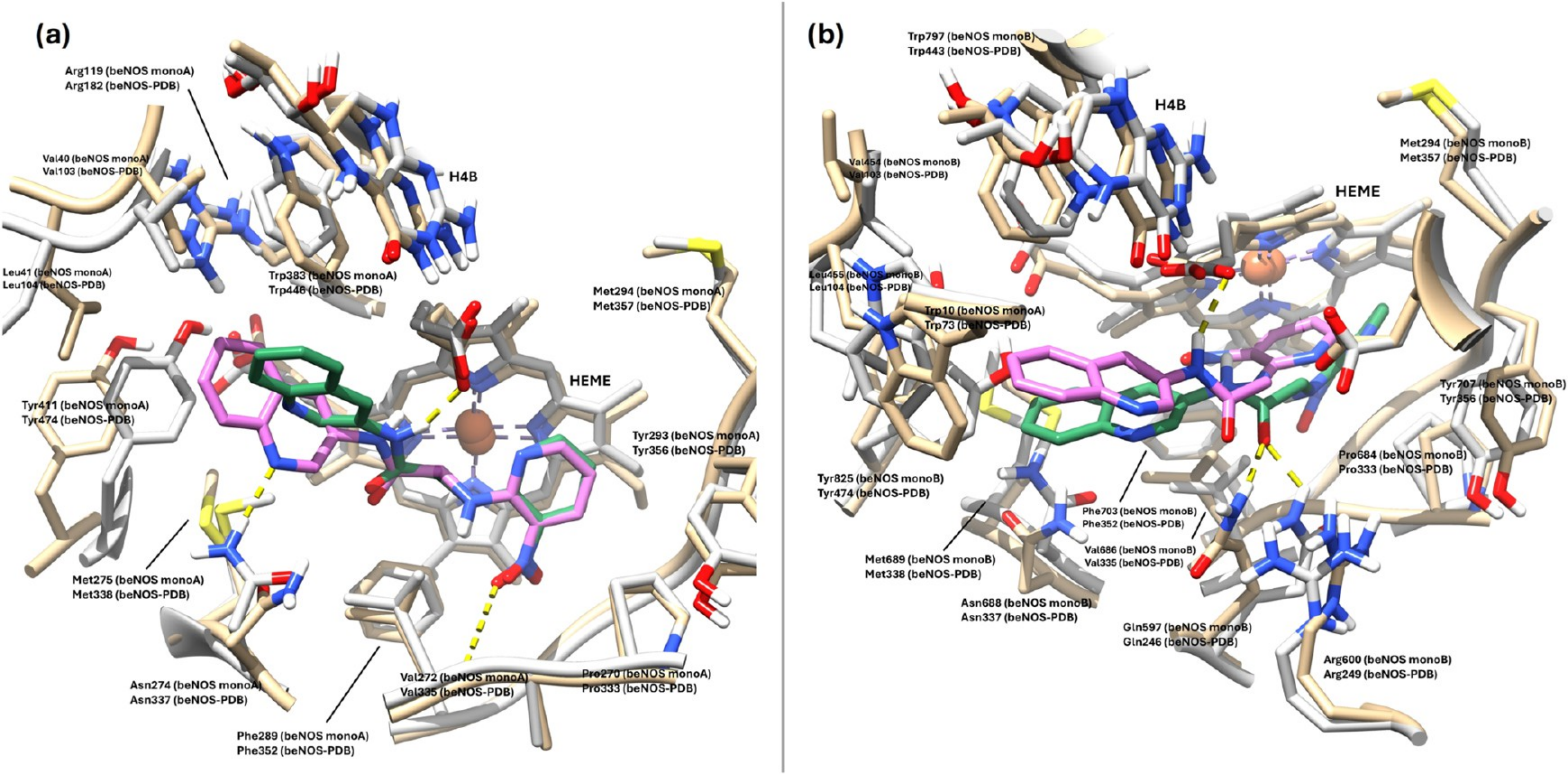
Representative cluster structures for beNOS (PDB 3E7S) obtained from *K* = 2 clustering: (a) monomer A and (b) monomer B. In each panel, the protein is shown in tan with compound **10** in green for cluster 0, and protein in gray with compound **10** in magenta for cluster 1.

## Conclusions

Although the involvement of iNOS in the pathogenesis of psoriasis is ascertained in different disease models, there is a lack of evidence that this enzyme could represent a therapeutic target and that its pharmacological modulation may lead to a remission of psoriasis. In the present work, we have disclosed new iNOS inhibitors with promising *in vitro* activity as antipsoriatic agents. The compounds were designed combining pharmacophoric moieties from known iNOS inhibitors, such as the 2-aminopyridine, indazole, and quinoline groups, and **6**, **7**, and **10** were revealed to be potent and selective compounds. The performed computational analysis shed light on the binding mode of the selected iNOS inhibitors **7** and **10** into both the iNOS and eNOS. The 2-amino,3-nitropyridine head can stabilize the molecules into the enzyme catalytic site by occupying the region underneath the heme cofactor, while the different orientation into the eNOS with respect to the iNOS of the imidazo­[4,5-*b*]­pyridine and 3-aminoquinoline rings of **7** and **10**, respectively, provide a plausible explanation for the observed isoform selectivity. Despite its slightly lower potency of action, compound **10** gave the best results with respect to **7** when evaluated on inflamed human keratinocytes, reducing the released NO levels as well as the cytokine-induced inflammatory responses and cells necrosis. This discrepancy between the in vitro enzymatic inhibition potency and the cellular biological activity of compounds **7** and **10** may stem from differences in their intracellular affinity for iNOS. Further mechanistic studies are warranted to clarify this point. Moreover, **10** could be effective both in reducing the activation of the immune response in inflamed keratinocytes, since it reduced the expression levels of adhesion molecules such as ICAM-1, HLA-DR, and MHC class I in the adopted cell model, and in resolving inflammation, as it modified the macrophage immunophenotype from the pro-inflammatory M1 one to the “pro-resolving” M2 one. Consistent with the *in vitro* findings, **10** also reduced inflammatory signatures in *ex vivo* models of skin inflammation, further confirming its therapeutic potential in immune-mediated skin diseases, such as psoriasis, supported by its reasonable metabolic stability and acceptable safety profile in the zebrafish model. Overall, the collected results confirm the relevance of iNOS as a target for the therapy of psoriasis and pave the way for future dermatological applications of compound **10**.

## Experimental Section

### General Methods and Materials for the Synthesis of Target Compounds

All chemicals were commercially available. 1400W was synthesized as previously reported.^28^ Chromatography was performed on silica gel 60 (Merck) and TLC on silica gel 60, F254. Melting points were determined on a Buchi apparatus and are given uncorrected. NMR spectra were run on a Varian instrument, operating at 300 (^1^H) or 75 (^13^C) MHz; chemical shifts (δ) are reported in ppm. HPLC was performed with a Waters (Milford, MA, USA) system composed of a P600 model pump, a 2996 photodiode array detector, and a 7725i model sample injector (Rheodyne, Cotati, CA, USA). The analyses were run on an Xterra MS C8 column (250 4.6 mm i.d., 5 mm particle size) (Waters), equipped with an Xterra MS C8 guard column (Waters). A column thermostat oven module Igloo-Cil (Cil Cluzeau Info Labo, France) was used. Target compounds purity was assessed by eluting the column with a mixture of CH_3_CN/15 mM sodium borate buffer (pH = 9.4) at a flow rate of 1 mL/min. All compounds are >95% pure by HPLC. Elemental analyses were carried out by a Eurovector Euro EA 3000 model analyzer. Analyses indicated by the symbols of the elements were within ± 0.4% of the theoretical values.

#### Benzyl {2-[(3-Aminopyridin-2-yl)­amino]-2-oxoethyl}­carbamate (**3**)

1-Ethyl-3-(3-(dimethylamino)­propyl)­carbodiimide (EDC) hydrochloride (4.6 mmol, 880 mg), hydroxybenzotriazole (4.6 mmol, 703 mg), and 4-dimethylaminopyridine (7.6 mmol, 934 mg) were added to a solution of the Z-Gly (3.8 mmol, 800 mg) in DMF dry (6 mL), at 0 °C, under N_2_. After 15 min stirring, 1,2 diamino-pyridine was added under the same conditions. The mixture was then allowed to react at room temperature for 20 h. Then, the solvent was removed under reduced pressure, and the residue was suspended in ethyl acetate (15 mL), and washed with Na_2_CO_3_ s.s. (15 mL) and brine (15 mL). The organic phase was dried over anhydrous Na_2_SO_4_, filtered, and evaporated. Finally, the crude was purified on silica gel (eluent: CH_2_Cl_2_/MeOH 9:1 saturated with NH_4_OH). The compound was obtained as a white solid oil; 73% yield. 1H NMR (300 MHz, CDCl_3_): δ 4.01 (d, *J* = 6.3 Hz, 2H), 5.14 (s, 2H), 5.73 (br s, 1H), 6.67 (dd, *J* = 5.1, 7.5 Hz, 1H), 7.34 (br s, 5H), 7.59 (d, *J* = 6.6 Hz, 1H), 7.89 (d, *J* = 5.1 Hz, 1H). ^13^C NMR (75 MHz, DMSO-d6): δ 44.29, 65.91, 112.53, 118.39, 128.14, 128.20, 128.75, 132.40, 137.42, 144.78, 154.06, 157.00, 168.89. Anal. Calcd for C_15_H_16_N_4_O_3_: C 59.99, H 5.37, N 18.66; found C 60.08, H 5.39, N 18.69.

#### Benzyl [(1H-1,3-Benzimidazol-2-yl)­methyl]­carbamate (**4**)

Compound **1** (1,48 mmol, 447 mg) was treated with glacial acetic acid (3 mL) at room temperature for 20 h. Then, the solvent was evaporated at reduced pressure, and the crude product was used without any further purification. Brown solid; 96% yield. M.P. 117–118 °C. 1H NMR (300 MHz, CD_3_OD): δ 4.61 (s, 2H), 5.13 (s, 2H), 7.25–7.37 (m, 6H), 7.94 (d, *J* = 7.8 Hz, 1H), 8.32 (d, *J* = 4.8 Hz, 1H). ^13^C NMR (75 MHz, DMSO-d6): δ 21.45, 66.08, 117.87, 128.20, 128.75, 137.32, 143.50, 156.83, 172.40. Anal. Calcd for C_16_H_15_N_3_O_2_: C 68.31, H 5.37, N 14.94; found C 68.57, H 5.40, N 14.91.

#### 1-(1H-1,3-Benzimidazol-2-yl)­methanamine (**5**)

Pd/C (0.16 mmol, 17 mg) was added at room temperature, under N_2_, to a solution of **4** (1.34 mmol, 458 mg) in CH_3_OH (10 mL). Gaseous H_2_ (1 bar) was bubbled into the solution for 3.5 h. Then, the mixture was filtered through Celite, and the filtrate was concentrated at dryness. The obtained crude was treated with 1,4 dioxane, obtaining an orange solid which was filtered off and used without further purification. 89% yield. Mp 109–111 °C. ^1^H NMR (300 MHz, DMSO-d6): δ 4.27 (s, 2H), 7.35–7.43 (m, 1H), 8.27–8.37 (m, 2H), 8.85 (s, 2H). ^13^C NMR (75 MHz, DMSO-d6): δ 36.59, 119.16, 127.75, 130.58, 138.88, 148.09, 154.60. Anal. Calcd for C_7_H_8_N_4_: C 56.74, H 5.44, N 37.81; found C 56.95, H 5.72, N 37.69.

#### General Synthesis of **6** and **7**

Compound **5** (0.769 mmol, 170 mg) was dissolved in DMF (3 mL) with TEA (3.076 mmol, 430 μL), and 2-chloropyridine (0.769 mmol, 88 mg) or 2-chloro,3-nitropyridine (0.769 mmol, 122 mg) was added. The mixture reacted at 90 °C for 4 h under stirring. Then, the solvent was removed under reduced pressure, and the crude product was purified on silica gel (eluent: CH_2_Cl_2_/MeOH 95:5).

#### N-[(1H-Imidazo­[4,5-*b*]­pyridin-2-yl)­methyl]­pyridin-2-amine (**6**)

Pale yellow solid, 19% yield. M.p.: 146–147 °C. ^1^H NMR (300 MHz, CD_3_OD): δ 4.80 (s, 2H), 6.59–6.65 (m, 2H), 7.25 (dd, *J* = 5.1, 8.4 Hz, 1H), 7.43–7.49 (m, 1H), 7.91 (dd, *J* = 0.9, 8.4 Hz, 1H), 7.95–7.98 (m, 1H), 8.30 (dd, *J* = 0.9, 5.4 Hz, 1H). ^13^C NMR (75 MHz, CD_3_OD): δ 39.61, 108.93, 112.96, 117.83, 137.32, 143.01, 146.81. Anal. Calcd for C_12_H_11_N_5_: C 63.99, H 4.92, N 31.09; found C 64.03, H 4.99, N 31.00.

#### N-[(1H-1,3-Benzimidazol-2-yl)­methyl]-3-nitropyridin-2-amine (**7**)

Yellow solid, 23% yield. M.p.: 175–177 °C. ^1^H NMR (300 MHz, DMSO-d6): δ 5.01 (d, *J* = 6.0 Hz, 2H), 6.80 (dd, *J* = 4.8, 8.1 Hz, 1H), 7.15 (dd, *J* = 4.8, 8.1 Hz, 1H), 7.85–7.88 (m, 1H), 8.23 (dd, *J* = 1.2, 4.8 Hz, 1H), 8.39 (dd, *J* = 1.5, 4.2 Hz, 1H), 8.47 (dd, *J* = 1.8, 8.1 Hz, 1H), 9.05 (t, *J* = 5.7 Hz, 1H). ^13^C NMR (75 MHz, DMSO-d6): δ 36.59, 104.25, 110.00, 113.08, 117.87, 129.06, 135.65, 140.66, 143.36, 143.84, 152.18, 156.06. Anal. Calcd for C_12_H_**10**_N_6_O_2_: C 53.33, H 3.73, N 31.10; found C 53.29, H 3.77, N 31.27.

#### Methyl 2-[(3-Nitropyridine-2-yl)­amino]­ethanoate (**8**)

To a solution of glycine methyl ester (1.593 mmol, 200 mg) and TEA (4.779 mmol, 660 μL) in DMF (2 mL), 2-chloro-3-nitropyridine (1.593 mmol, 253 mg) was added, and the mixture was reacted under magnetic stirring at 90 °C, for 3 h. Then, it was diluted with H_2_O until a precipitate was obtained, which was filtered off, washed with cold H_2_O, and dried. Yellow solid, 78% yield. M.p.: 112–113 °C. ^1^H NMR (CDCl_3_): δ 3.79 (s, 3H), 4.39 (d, J = 5.7 Hz, 2H), 6.73 (dd, J = 4.5, 8.1 Hz, 2H), 8.40 (d, *J* = 3.9 Hz, 1H), 8.44 (d, *J* = 8.1 Hz, 1H), 8.49 (br s, 1H). ^13^C NMR (CDCl_3_): δ 47.36, 56.73, 117.08, 133.17, 139.55, 156.24, 159.58, 174.78. Anal. Calcd for C_8_H_9_N_3_O_4_: C 45.50, H 4.30, N 19.90; found: C 45.66, H 4.38, N 19.77.

#### 2-[(3-Nitropyridine-2-yl)­amino]­ethanoic Acid (**9**)

NaOH 5% (w/v) (1.136 mmol, 0.910 mL) was added to a solution of **8** (1.136 mmol, 240 mg) in methanol (3.0 mL), and the mixture was stirred at room temperature for 3 h. Then, the solvent was evaporated, and HCl 1 N was added until a precipitate was obtained, which was filtered off, washed with H_2_O, and crystallized by ethyl acetate/*n*-hexane. Light brown solid. 65% yield. M.p.: 126–127 °C. ^1^H NMR (DMSO-d6): δ 4.20 (d, *J* = 5.7 Hz, 2H), 6.80 (dd, *J* = 4.5, 8.1 Hz, 1H), 8.43–8.46 (m, 2H), 8.68 (t, *J* = 5.7 Hz, 1H), 12.69 (br s, 1H). ^13^C NMR (CDCl_3_): δ 57.3, 117.21, 132.9, 139.7, 156.0, 159.8, 175.1. Anal. Calcd for C_7_H_7_N_3_O_4_ C 42.65, H 3.58, N 21.31; found C 42.73, H 3.22, N 21.18.

#### N-(Quinolin-3-yl)-2-[(3-nitropyridin-2-yl)­amino]­ethanamide (**10**)

To a solution of **9** in DMF dry (3 mL), *i*-BuOCOCl (0.609 mmol, 100 μL) and NMM (0.609 mmol, 70 μL) were added at – 15 °C, under N_2_, with magnetic stirring. After 15 min, 3-aminoquinoline (0.609 mmol, 88 mg) was added under the same conditions, and then the mixture was reacted for 20 h at 0 °C, and for a further 4 h at room temperature. Then, the mixture was filtered, and the filtrate was concentrated to dryness. The crude product was dissolved in ethyl acetate (10 mL) and washed with NaOH 1 N (2 x10 mL) and NaCl s.s. (10 mL). The organic layer was dried over Na_2_SO_4_, and then the solvent was removed under reduced pressure. The residue was purified on a silica gel column (eluent: CH_2_Cl_2_/AcOEt 1:1). Light brown solid, 21% yield. M.p.: 181–182 °C. ^1^H NMR (CDCl_3_): δ 4.01 (d, *J* = 6.6 Hz, 2H), 6.75 (dd, *J* = 4.8, 8.7 Hz, 1H), 7.22 (br s, 1H), 7.26 (d, *J* = 0.9 Hz 1H), 7.55 (t, *J* = 8.1 Hz, 1H), 7.62 (t, *J* = 8.1 Hz, 1H), 7.79 (d, *J* = 7.5 Hz, 1H), 8.05 (d, *J* = 8.7 Hz, 1H), 8.35 (d, *J* = 4.8 Hz, 1H), 8.43 (d, *J* = 8.7 Hz, 1H), 8.57 (br s, 1H), 8.77 (d, *J* = 1.8 Hz, 1H). ^13^C NMR (CDCl_3_): δ 71.83, 98.77, 113.43, 122.60, 127.41, 127.51, 128.14, 128.34, 128.46, 131.92, 135.10, 162.00. Anal. Calcd for C_16_H_13_N_5_O_3_: C 59.44, H 4.05, N 21.66; found C 59.24, H 3.99, N 21.75.

#### (1H-Indazol-6-yl)­methanamine (**11**)

1H-indazole-6-carbonitrile (3.5 mmol, 500 mg) was dissolved in dry THF (6 mL) and added to a solution of LiAlH_4_ 2 M in dry THF (3.5 mmol, 8 mL) at 0 °C, and the resulting mixture was stirred under N_2_. Then, the reaction temperature was set at room temperature for 2 h; subsequently, further dry THF (10 mL) was added, and the mixture was stirred under reflux for 4 h. The reaction was quenched by H_2_O (10 mL) and the resulting suspension was filtered over Celite. The filtrate was evaporated at reduced pressure, and the crude product was purified by column chromatography on silica gel (dichloromethane/methanol, 9:1). White solid; 65% yield. M.p.: 130–135 °C; ^1^H NMR (CD_3_OD): δ 3.98 (s, 2H), 7.17–8.0 (m, 4H); ^13^C NMR (CD_3_OD): δ 45.2, 108.6, 120.9, 121.1, 122.5, 133.5, 139.4, 140.8. Anal. Calcd for C_8_H_9_N_3_: C 65.29, H 6.16, N 28.55; found C 65.17, H 6.08 N, 28,48.

#### General Procedure for the Synthesis of **12**, **13**, and **15**

EDC hydrochloride (1 mmol, 195 mg) and DMAP (1 mmol, 125 mg) were added under N_2_, at 0 °C, to a solution of proper carboxylic acid (1 mmol) in dry DMF (5 mL). The mixture was stirred for 15 min, and then, a solution of **11** (1 mmol, 150 mg) in dry DMF (1 mL) was added. Subsequently, the mixture was brought to room temperature and reacted for 24 h. The solvent was removed under reduced pressure, and the residue was dissolved in ethyl acetate (10 mL) and washed with Na_2_CO_3_ s.s. (3 × 10 mL) and NaCl s.s. (10 mL). The organic layer was dried over Na_2_SO_4_, and then the solvent was removed under reduced pressure.

#### N-[(1H-Indazol-6-yl)­methyl]-2-phenylacetamide (**12**)

The crude product was purified on a silica gel column (eluent: CH_2_Cl_2_/MeOH 9:1). White solid; 73% yield. M.p.: 145–146 °C; ^1^H NMR (CD_3_OD): δ 3.55 (s, 2H), 4.48 (s, 2H), 7.03 (d, J = 8.1 Hz, 1H), 7.19–7.30 (m, 5H), 7.41 (s, 1H), 7.67 (d, *J* = 8.7 Hz, 1H), 7.98 (s, 1H). ^13^C NMR (CD_3_OD): δ 42.5, 43.1, 108.1, 120.4, 120.6, 122.1, 126.5, 128.1, 128.6, 133.3, 135.5, 137.6, 140.4, 172.5. Anal. Calcd for C_16_H_15_N_3_O C 72.43, H 5.70, N 15,84; found C 72.71, H 5.57, N 15,56.

#### *tert*-Butyl 4-{[(1H-indazol-6-yl)­methyl]­amino}-4-oxobutanoate (**13**)

The crude product was purified on a silica gel column (eluent: CH_2_Cl_2_/MeOH 9:1). White solid; 81% yield. M.p.: 151–152 °C; ^1^H NMR (CDCl_3_): δ 1.41 (s, 9H,), 2.51 (t, *J* = 6.3 Hz, 2H), 2.62 (t, *J* = 6.3 Hz, 2H), 4.56 (d,, *J* = 6.3 Hz, 2H), (br, 1H), 7.10 (d, *J* = 8.7 Hz, 1H), 7.47 (s, 1H), 7.68 (d, 1H, *J* = 8.7 Hz), 8.09 (s, 1H). ^13^C NMR (CD_3_OD): δ. 28.3, 29.1, 30.5, 42.4, 81.5, 108.3, 120.4, 121.2, 121.9, 132.5, 139.4, 142.1, 172.4; 175.1. Anal. Calcd for C_16_H_21_N_3_O_3_ C 63.35, H 6.98, N 13.85; found C 63.61, H 7.01, N 13.61.

#### Methyl 4-{[(1H-Indazol-6-yl)­methyl]­carbamoyl}­benzoate (**15**)

The crude product was purified on a silica gel column (eluent: CH_2_Cl_2_/Ethyl Acetate 8:2). White solid; 69% yield. M.p.: 178–179 °C; ^1^H NMR (CDCl_3_): δ 3.91 (s, 3H), 4.25 (d, *J* = 6.6 Hz, 2H), 7.66 (d, *J* = 8.1 Hz, 1H), 7.87 (d, *J* = 8.4 Hz, 2H), 8.04 (d, *J* = 8.4 Hz, 2H), 8.12 (s, 2H). ^13^C NMR (CD_3_OD): δ 19.4, 43.7, 108.2, 121.8, 122.5, 126.9, 129.1, 133.2, 136.5, 138.7, 140.2, 167.1, 167.7. Anal. Calcd for C_17_H_15_N_3_O_3_ C 66.01, H 4.89, N 13.58; found C 66.11, H 4.97, N 13.21.

#### 4-{[(1H-Indazol-6-yl)­methyl]­amino}-4-oxobutanoic Acid

(14)­To a solution of **13** (0.62 mmol, 188 mg) in dichloromethane (6 mL) was dropwise added trifluoroacetic acid (1 mL) at 0 °C, under magnetic stirring. The mixture was reacted for 12 h at room temperature, and then the solvent was removed at reduced pressure. The crude product was triturated with diethyl ether, affording the desired compound. White solid; 89% yield. M.p.: 167–168 °C; 1H NMR (CD_3_OD): 2.52 (t, *J* = 5.7 Hz, 2H), 2.61 (t, J= 6.3 Hz, 2H), 4.50 (s, 2H), 7.09 (dd, *J* = 0.9 Hz; 8.1 Hz, 1H), 7.46 (s, 1H), 7.70 (d, *J* = 7.8 Hz, 1H), 8.04 (d, *J* = 1.2 Hz, 1H). ^13^C NMR (CD_3_OD): δ. 28.8, 30.1, 42.9, 108.1, 120.5, 120.9, 121.9, 132.9, 138.2, 140.4, 173.0, and 174.8. Anal. Calcd for C_12_H_13_N_3_O_3_ C 58.29, H 5.30, N 17.00; found C 58.15, H 5.27, N 17.22.

#### 4-{[(1H-Indazol-6-yl)­methyl]­carbamoyl}­benzoic Acid (**16**)

NaOH 5% (w/v) (0.31 mmol, 0.25 mL) was added to a solution of **15** (0.26 mmol, 80 mg) in THF (4 mL), and the mixture was stirred at room temperature for 1 h. Then, the solvent was evaporated, and the residue was treated with HCl 1 N, until a solid was obtained. The last was filtered, washed with H_2_O and crystallized by a mixture of CH_2_Cl_2_/methanol. White solid; 75% yield. M.p.: 189–191 °C; ^1^H NMR (DMSO-d6): 4.58 (s, 2H), 7.06 (d, *J* = 6.3 Hz, 1H), 7.40 (s, 1H), 7.66 (d, *J* = 7.5 Hz, 1H), 7.98 (s, 4H), 9.26 (s, 1H, CHAr), 13.03 (br, 1H, OH). ^13^C NMR (CD_3_OD): δ 43.4, 108.5, 120.8, 122.3, 127.9, 129.7, 133.6, 137.9, 138.4, 140.6, 166.0, 167.2. Anal. Calcd for C_16_H_13_N_3_O_3_ C 65.08, H 4.44, N 14.23; found C 65.12, H 4.38, N 14.20.

### Biological Experimental Section

#### NOS Inhibition Assay

Recombinat human iNOS was purchased from Enzo Life Sciences, Inc. (New York, USA). Recombinant bovine eNOS were purchased from Cayman Chemical (Ann Arbor, USA). To measure iNOS activity, 10 μL of enzyme stock solution were added to 80 μL of 2-[4-(2-hydroxyethyl)­piperazin-1-yl]­ethanesulfonic acid (HEPES) buffer pH = 7.4, 100 mM, containing 0.1 mM CaCl_2_, 1 mM D,L-dithiothreitol (DTT), 0.5 mg/mL BSA, 10 μM flavin mononucleotide (FMN), 10 μM flavin adenine dinucleotide (FAD), 30 μM tetrahydrobiopterin (BH4), 10 μg/mL calmodulin (CaM), 10 μM l-Arg. For the eNOS activity evaluation, 25 μL of the enzyme stock solution were added to 65 μL of HEPES buffer containing 2 mM CaCl_2_ and the same cofactors cocktail used for the iNOS assay. Then, 10 μL of the test compound solution (0.01–10 μM) were added to the enzyme assay solution, followed by preincubation of 15 min at 37 °C. Each reaction was initiated by the addition of 10 μL of NADPH 7.5 mM, carried out at 37 °C for 30 min, and stopped by adding 500 μL of ice-cold CH_3_CN. The mixture was brought to dryness under *vacuum* and eventually stored at – 20 °C, before the HPLC analysis.^30^

#### Metabolic Stability

The stability of **10** in liver microsomes was measured according to a literature method.^46^ Briefly, 0.5 μL of the compound (2 mM in DMSO stock solution) was diluted with PBS (432 μL) and a 13 μL aliquot of Sprague–Dawley rat liver microsomes (Sigma-Aldrich, no. M9066) was added. The tube was vortexed at 37 °C for 5 min, and then NADPH (50 μL, 10 mM in PBS stock solution) was added. The mixture was incubated at 37 °C for 60 min and quenched by 250 μL of ice-cold CH_3_CN and centrifuged at 6000 rpm for 10 min. The supernatant was then analyzed by PDA-HPLC on an Atlantis dC_18_ column (250 × 4.6 mm i.d., 5 μm particle size) (Waters). The HPLC column was eluted at a flow rate of 1 mL/min using a mixture of CH_3_CN and Milli-Q H_2_O (70:30). The injection volume was 5 μL. The procedure was repeated at least three times, and verapamil was used as a positive control. The microsomal intrinsic clearance (CL_int,micr_) was calculated according to the following equation:

$${CL}_{\mathrm{int},\mathrm{micr}}=\frac{0.693}{t_{1/2}}\times \frac{\mathrm{volume}\;\mathrm{of}\;\mathrm{incubation}\;\mathrm{medium}}{\mathrm{mg}\;\mathrm{microsomal}\;\mathrm{protein}}$$

Finally, CL_int,micr_ was scaled to intermediate *in vivo* intrinsic (hepatic) clearance (CL_int_), using suitable scaling factors obtained from the literature,^55^ according to the following equation:

$${\mathrm{CL}}_{\mathrm{int}}={CL}_{\mathrm{int},micr}\times \frac{mg\;microsome}{g\;liver}\times \frac{liver\;weight\;\left(g\right)}{body\;weight\;\left(Kg\right)}$$

where 45 mg of microsomal protein per gram of liver tissue and 40 g of liver tissue per kilogram of body weight were applied.

#### Cell Cultures, Ex Vivo Skin Explants, and Treatments

A cell line of undifferentiated human monocytes (CRL-9855) was purchased from ATCC and subcultured in complete RPMI 1640 (Merck, Darmstadt, Germany) supplemented with 10% heat-inactivated fetal bovine serum (FBS), 1% penicillin/streptomycin, and 1% sodium pyruvate (all from Gibco, Invitrogen, Life Technologies, Carlsbad, CA, USA) at 37 °C and 5% CO_2_. For differentiation into macrophages, monocytes were seeded in multiwell culture plates and stimulated with 100 ng/mL of PMA (phorbol-12-myristate-13-acetate, purchased from Merck, Darmstadt, Germany, stock solution 1 mM in DMSO) as previously described.^56^ Both cell lines were subsequently exposed to the growth medium (CTRL) and to **7** or **10** (0.1–1–5–10–20–50–100 μM final dilutions from the stock solution in DMSO 100 mM).

To establish an inflamed environment, macrophages were stimulated with LPS of 0.5 μg/mL (lipopolysaccharide from *E. coli*, purchased from Merck, Darmstadt, Germany, stock solution 1 mg/mL in water) and further exposed to compounds **7** and **10** (0–100 μM).

A cell line of human keratinocytes (HaCaT cells) was purchased from Cytion and subcultured in DMEM high glucose (Merck, Darmstadt, Germany) supplemented with 10% heat-inactivated fetal bovine serum (FBS) and 1% penicillin/streptomycin at 37 °C and 5% CO_2_. To establish pro-inflammatory conditions, cells were stimulated by TNF-α (10 ng/mL), IFN-γ (10 ng/mL), and IL-17 (50 ng/mL) as described previously,^33^ and subsequently exposed to treatments.

Human keratinocytes were obtained from skin biopsies of healthy donors undergoing abdominoplastic surgery, as previously reported.^57^ Skin biopsies were obtained after patient’s informed written consent, with the approval of the IDI-IRCCS Local Ethics Committee (Prot. N. CE-475), and in conformity with the Helsinki guidelines. Second-passage keratinocytes were used in all experiments and cultured in the serum-free medium KGM (Clonetics, San Diego, CA), for at least 3–5 days (at 60–80% confluence) before performing treatment with iNOS inhibitors and cytokines. Cells were starved for 18 h in keratinocyte basal medium (KBM-GOLD, Clonetics), and treated with different concentrations (1, 10, and 50 μM) of iNOS **7**, **10**, and 1400W inhibitors. In some experiments, cell cultures were stimulated with 50 ng/mL recombinant human (rh) IL-17A, 200 U/mL rh IFN-γ or 50 ng/mL rh TNF-α (all from R&D Systems, Minneapolis, MN) in KBM for different time of stimulation depending on the experiments.

*Ex vivo* skin models were established by using 6 mm punch skin explants of healthy volunteers (*n* = 3) undergoing abdominoplasty at IDI-IRCCS (Prot. CE-475/2016). Skin explants were treated for 3 h with a combination of rh IFN-γ (1000 U/mL), rh TNF-α, and IL-17A (both at 250 ng/mL) and, in the presence of 20 μM iNOS **7**, **10**, and 1400W inhibitors or vehicle alone (DMSO). Samples were divided into two equal parts for subsequent immunohistochemistry (IHC) and quantitative reverse transcription-PCR (qRT-PCR) analyses.

Cytotoxicity of **7**, **10**, and 1400W compounds at 1, 10, and 50 μM doses was tested by measuring the activity of lactate dehydrogenase (LDH) released from keratinocyte cultures after 24, 48, and 72 h of inhibition, using Cytotoxicity Detection Kit Plus-LDH (Roche Diagnostics, Milan, Italy) following the manufacturer’ instructions.

#### Cell-Cycle Analysis of Keratinocyte Cultures

To determine cell-cycle distribution analysis, second-passage keratinocytes were cultured in 12-well plates and, at 60–80% confluence, treated with 1, 10, and 50 μM **7**, **10**, and 1400W compounds for 24 h. After treatment, cells were collected by trypsinization, fixed in 70% ethanol, washed in PBS, resuspended in PBS containing 1 mg/mL RNase and 50 μg/mL propidium iodide (PI). Then, cells were incubated in the dark for 30 min at room temperature and analyzed by flow cytometry (Accuri C6 Flow cytometer, Becton Dickinson, Mountain View, CA). The data were analyzed using Multicycle software (Phoenix Flow Systems, San Diego, CA).

#### Apoptosis Analysis

Human keratinocytes were cultured in 12-well plates, and at 60–80% confluence, cells were starved for 18 h in KBM, and treated with 10 and 50 μM **7**, **10**, and 1400W for 24 h. For experiments evaluating the effects of iNOS compounds on cytokine-induced apoptosis in keratinocytes, 10 μM **10** and 1400W iNOS inhibitors were added to keratinocyte cultures together with IFN-γ and TNF-α for 24 h. Apoptosis of keratinocytes was evaluated using an FITC Annexin V/propidium iodide (PI) apoptosis detection kit (BD Biosciences, Milan, Italy). Viable (Annexin V/PI^–^), necrotic (PI^+^), and apoptotic (early, Annexin V+, and late, Annexin/PI^+^) cells were analyzed by flow cytometry with the Accuri C6 flow cytometer equipped with Cell Quest software (Becton Dickinson).

#### Cell Metabolic Activity (MTT Assay)

Undifferentiated monocytes and macrophages were seeded (5 × 10^3^ cells/well) in 96-multiwell culture plates (Corning Falcon, Glendale, USA) and exposed to treatments as previously described in this section under basal and pro-inflammatory conditions. At the established time points (24–48 h), the exposure media were replaced by fresh RPMI containing 3-(4,5-dimethylthiazol-2-yl)-2,5-diphenyltetrazolium bromide (MTT) 0.5 mg/mL (Merck, Darmstadt, Germany) and incubated for 3 h at 37 °C and 5% CO_2_. Then, the MTT solution was removed and replaced with 100 μL/well of DMSO and gently swirled for 10 min. The optical density in each well was immediately measured by using a spectrophotometer (Thermo Fisher Scientific, Waltham, MA, USA) at a wavelength of 540 nm. Each experiment was performed three times in triplicate per experimental condition (n = 9).

#### Immunophenotyping In Vitro by Flow Cytometry

The expression of surface markers (CDs) in differentiated macrophages was analyzed by flow cytometry. After differentiation, macrophages were stimulated with LPS and exposed to compounds **7** and **10** at 0.1, 50, and 100 μM for 24 h. Then, cells were harvested with a Stem Pro Accutase cell dissociation reagent (Thermo Fisher Scientific, Waltham, MA, USA), collected by centrifugation in the cold, and washed once with flow cytometry buffer made by HEPES buffer at pH 7.4, 140 mM NaCl, and 2.5 mM CaCl_2_. Cells were incubated with fluorochrome-conjugated antibodies (1:50 dilutions) in 50 μL of flow cytometry buffer for 15 min in the dark. Cells were stained separately in each single screening tube with cluster of differentiation (CD)­80-PE, CD163-PE, and CD73-PE (all purchased by BD Biosciences, MA, USA) Then, the excess of antibodies was removed by adding fresh flow cytometry buffer and centrifugation. After that, approximately 20,000 events were run in a Beckman Coulter CytoFLEX flow cytometer (CA, USA). Relative fluorescence emissions of gated cells by forward and side scatter properties (FSC/SSC) were analyzed using CytExpert Software (Beckman Coulter) and expressed as the mean fluorescence intensity (MFI) ratio on the isotype control. Individual values obtained from three independent experiments (n = 3) were summarized as means and standard deviations.

The expression of membrane markers on keratinocytes was analyzed by flow cytometry in cultures 24 h stimulated with a mix of IFN-γ and TNF-α cytokines in the presence of 10 μM **7**, **10**, and 1400W or vehicle alone. Keratinocyte membrane expression of ICAM-1, HLA-DR, and major histocompatibility complex (MHC) class I was evaluated using FITC-conjugated anti-CD54 (clone 84H10; Immunotech, Marseille, France) and anti-HLA-DR (clone L243, BD Pharmingen, Franklin Lakes, NJ, USA) monoclonal Abs, respectively, whereas MHC class I expression on keratinocyte membrane was detected by using APC-conjugated antihuman MHC class I (clone 51-10C9, BD Pharmingen). Keratinocytes were analyzed using the Accuri C6 Flow cytometer (BD) equipped with Cell Quest software (BD).

#### Immunohistochemistry

Human skin samples were fixed with 10% formalin, prior to embedding in paraffin. 5-μm sections were dewaxed and rehydrated, then incubated with primary anti-p-STAT1 (Tyr701, #9167, Cell Signaling Technologies) or anti-p-STAT3 (Tyr705, #9145, Cell Signaling Technologies). Secondary biotinylated Ab and staining kits (Vector Laboratories, Burlingame, CA, USA) were used to develop immunoreactivities. Sections were stained with Mayer’s hematoxylin and eosin (H&E) and were visually analyzed by two pathologists experienced in dermatology, and positivity was evaluated. For each skin specimen, two sections were analyzed, and positive cells were counted in three adjacent fields at a magnification of 200X.

#### Interleukin-6 Secretion

Cell culture supernatants from wells used to perform the MTT assay were collected after 24 h and analyzed for cytokine release. The amount (pg/mL) of interleukin-6 (IL-6) was quantified using commercial ELISA kits (Enzo Life Sciences Inc., Lausen, Switzerland) as already reported.^58^

#### RNA Isolation and Real-Time Polymerase Chain Reaction (PCR)

Total RNA was extracted from keratinocyte cultures treated with iNOS **7**, **10**, and 1400W inhibitors (all at 10 μM concentration) and/or IFN-γ alone or together with TNF-α, and by a mix of three cytokines IFN-γ, TNF-α, and IL-17A, using the TRIzol reagent (InVitrogen, Waltham, MA, USA). mRNA was reverse-transcribed into cDNA and analyzed by real-time PCR. The expression of inflammatory genes CXCL8, CCL2, and CXCL10, as key skin-homing chemokines, ICAM-1 as immunomodulatory molecule, and iNOS in stimulated keratinocytes was evaluated by ABI Prism SDS 7000 PCR instrument (Applied Biosystems, Branchburg, NJ), using SYBR Green PCR reagents (Applied Biosystems). The forward and reverse primers employed for real-time PCR were as follows: for CXCL8, 5′-CTCTGTGTGAAGGTGCAGTTTT-3′ and 5′-GGGTGGAAAGGTTTGGAGTAT-3′; for CCL2, 5′-CACCAGCAGCAAGTGTCCC-3′ and 5′-CCATGGAATCCTGAACCCAC-3′; for CXCL10, 5′-TGGCATTCAAGGAGTACCTCTCT-3′ and 5′-CTGATGCAGGTACAGCGTACG-3′ for NOS2, 5′-CTTATTCAGCTGTGCCTTCAA-3′ and 5′-CGATTTCTTCAGTTTCTCTCC-3′; for ICAM-1, 5′-GCTTCGTGTCCTGTATGGCC-3′ and 5′-TTTCCCGGACAATCCCTCTC-3′; for HPRT1, 5′- TCCTCAGACCGCTTTTTGCC-3′ and 5′-ATCGCTAATCACGACGCT GG-3′. mRNA levels were normalized to HPRT1 mRNA, the values obtained from triplicate experiments were averaged, and data are presented as mean 2−ΔΔCT ± SD.

#### In Vivo Assay in Zebrafish

*In vivo* assay was performed on zebrafish embryos and larvae obtained from adult AB wild-type pairs sourced from the European Zebrafish Resource Center (Karlsruhe Institute of Technology, Germany) and bred at the zebrafish facility of the University of Milan-Bicocca (ethical approval ATS MetroMilano Prot. n. 0020984-12/02/2018), as reported in Negrini et al.^59^ For each treatment, compound **10** was freshly dissolved in DMSO at the 1 mM concentration (stock) that was then diluted in embryo solution (ES, 100 mg/L NaHCO_3_, 100 mg/L Instant Ocean salt, 190 mg/L CaSO_4_) to achieve final working concentrations ranging from 1 to 30 μM. To explore the effects of **10** on zebrafish development, an assay was performed using a 24-well plate, with one embryo per well and 20 embryos per condition. Each embryo was incubated at 26 °C in 2 mL of ES medium (control), ES medium supplemented with **10** (treatment), or DMSO at a final concentration of 0.13% v/v (vehicle control). Two exposure conditions were applied: the first starting from 1–3 h post fertilization (hpf) up to 96 hpf according to OECD n 236 ^60^ and the second starting from 48 hpf until 120 hpf. All treatment and control conditions were performed in biological and technical triplicate. Lethal end points indicating acute toxicity (coagulation of fertilized eggs, lack of somite formation, lack of detachment of the tail-bud from the yolk sac, and lack of heartbeat) were inspected through a stereomicroscope every 24 h until the end of the test. Besides, additional sublethal end points such as edemas and defects to spinal cord, tail, heart, eyes, and head were screened and reported at 96 or 120 hpf. All experiments were performed on embryos within 120 h post fertilization (hpf), thus were not subject to animal experimentation rules according to European and Italian directives (E. Commission, Directive 2010/63/EU of the European Parliament and of the Council of 22 September 2010 on the protection of animals used for scientific purposes, Official Journal of the European Union, 2010, L276).

#### Statistics

Statistics were performed using the one-way analysis of variance (ANOVA) followed by the Tukey’s multiple comparison test by means of the Prism 8.0 software (GraphPad, San Diego, CA, USA). Results are the mean values ± standard deviations. Values of *p* ≤ 0.05 were considered statistically significant.

#### Docking Study

Autodock 4.2.6 (AD4)^61^ was used for the docking studies on human iNOS (hiNOS) and bovine eNOS (beNOS) isozymes (pdb IDs 4CX7 and 3E7S respectively). Ligands structures were built on Avogadro^62^ and optimized using Gaussian 09 ^63^ (HF/6-31G­(d,p)). Once optimized, ligands PDBs were prepared for docking using the prepare_ligand4.py script included in MGLTools 1.5.4.^61^ Protein structures, on the other hand, were prepared for docking using MOE 2024 QuickPrep module.^64^ Water molecules and nonrelevant ligands were removed, while the heme and H4B cofactors were retained; protonation states were assigned at pH 7.4 and hydrogen atoms were added accordingly (polar hydrogens added and nonpolar hydrogens merged during AD4 preparation). The produced structures were saved as.pdb files and prepared for docking using the prepare_receptor4.py script from MGLTools. The Fe atom of heme was assigned a charge of +2. AD4 was used to automatically dock the ligands into the hiNOS and beNOS binding sites. For both enzymes, the docking grid was centered on the ligand binding site and defined as 70 × 70 × 70 grid points with 0.375 Å spacing. In all calculations, the Lamarckian genetic algorithm local search (GALS) method was used for the docking calculations. AD4 parameter file was set to 10 GA–LS runs, 2,500,000 energy evaluations, 27,000 generations, and a population size of 150. A Solis and Wets local search of 300 rounds was applied with a probability of 0.06. A mutation rate of 0.02 and a crossover rate of 0.8 were used. The docking results from each of the 10 calculations were clustered based on root-mean-square deviation (RMSD) solutions differing by less than 2.0 Å between the Cartesian coordinates of the atoms and ranked according to their AD4 predicted binding energy (docking score). The representative conformations of the most favorable clusters were subsequently prioritized based on structural consistency with the conserved binding mode of related 2-aminopyridine inhibitors described in the literature.^52,53,66^ Docking protocol validation was performed by cross-docking the reference iNOS inhibitor BYK191023 into hiNOS (PDB 4CX7) and comparing the resulting pose with its crystallographic conformation in murine iNOS (PDB 3NW2), as described in the Results/Analysis section.^67,68^ This structural assessment and figure generation were performed using UCSF Chimera 1.18.^65^

#### Molecular Dynamics Simulations

Initial protein–ligand complexes for both isoforms were taken from the molecular docking results of compound **10** in the iNOS and eNOS oxygenase domains (human iNOS, PDB entry 4CX7; bovine eNOS, PDB entry 3E7S). Dimeric oxygenase-domain assemblies containing the structural ZnS4 site, heme and tetrahydrobiopterin (H4B) cofactors, and one copy of compound **10** per monomer were generated by enforcing C2 symmetry in PyMOL (v3.0.0 Open-Source)^69^ and standardized for AMBER (AMBER 24; AmberTools 22)^70–72^ with pdb 4amber. Protonation states were assigned at a physiological pH (7.4). Protein parameters were taken from the AMBER ff14SB force field;^73^ heme parameters were taken from the AmberTools ″hemall″ set and included the axial Cys-Fe linkage. The ZnS4 site was described with a bonded model generated with MCPB.py^74^ using Gaussian-derived force constants (B3LYP/6-31G*)^75–78^ and RESP charges. Compound **10** and H4B parameters were generated with GAFF2 ^79^ using AM1-BCC partial charges.^80^ Each system was solvated in a water box (10 Å buffer), neutralized, and supplemented with NaCl to approximate physiological ionic strength.

### Simulation Protocol

Energy minimization was performed in two stages (solvent/ions relaxed under solute restraints, followed by unrestrained minimization). Systems were heated from 0 to 310 K under NVT with harmonic restraints on the solute, followed by short NPT density relaxation at 310 K and 1 bar and a 1 ns unrestrained equilibration. Production simulations were carried out in the NPT ensemble as independent replicas initiated with randomized velocities. Temperature was controlled with Langevin dynamics; bonds involving hydrogen were constrained (SHAKE),^81^ enabling a 2 fs time step. Periodic boundary conditions were applied with a 10 Å nonbonded cutoff. Coordinates were saved every 10 ps for analysis.

### Trajectory Processing and Analysis

Trajectories were reimaged and centered with cpptraj^82^ (autoimage anchored to the protein–cofactor–ligand complex) and stripped of solvent and ions to generate analysis-ready trajectories/topologies. Analyses were performed using cpptraj and custom Python workflows (MDAnalysis),^83,84^ including RMSD/RMSF, solvent-accessible surface area, radius of gyration, protein–ligand contact metrics, and conformational clustering. Representative cluster structures were rendered with UCSF Chimera (v1.18).^65^

## Supplementary Material





## Notes

HPLC analysis of the NOS assay; generation of nitric oxide in HaCaT cells under inflamed conditions in the presence of compound **10** and Figure S1. Figure S2: iNOS inhibitors slightly reduces STAT1 and STAT3. Molecular Dynamics analysis and Figures S3–S18. NMR spectra and HPLC trace for the target compounds. Dose–response curves for iNOS inhibition. Prediction of physicochemical and pharmacokinetic properties and Table S2 (PDF)
Table S1: Residue equivalence mapping between hiNOS (4CX7) and beNOS (3E7S) across ensemble numbering, PDB numbering, and monomer offsets (XLSX)

## Abbreviations

TNF-α: tumor necrosis factor (TNF)-α
IL: interleukin
CCL2: CC chemokine ligand 2
CXCL10: C-X-C motif chemokine ligand
CXCL8: C-X-C motif chemokine ligand
DMAP: 4-dimethylaminopyridine
DMF: dimethylformamide
EDC HCl: 1-Ethyl-3-(3-(dimethylamino)­propyl)­carbodiimide hydrochloride
eNOS: endothelial nitric oxide synthase
GlyOMe: glycine methylester
HPRT1: hypoxanthine phosphoribosyltransferase 1
HEPES: (2-hydroxyethyl)-1-piperazineethanesulfonic acid
hiNOS: human inducible nitric oxide synthase
i-BuOCOCl: isobuthylchloroformate
ICAM-1: intracellular adhesion molecule 1
KBM: keratinocyte basal medium
KGM: keratinocyte growth medium
LDH: lactate dehydrogenase
LPS: lipopolysaccharide
MHC: major histocompatibility complex
NF-κB: nuclear factor kappa-light-chain-enhancer of activated B cells
NMM: *N*-methylmorpholine
MTT: 3-(4,5-dimethylthiazol-2-yl)-2,5-diphenyltetrazolium bromide
nNOS: neuronal nitric oxide synthase
NO: nitric oxide
OHBt: hydroxybenzotriazole
PI: propidium iodide
TEA: triethylamine
TLC: thin layer chromatography
