# Supplementary material for: Synthesis, Biological Evaluation, and Computational Study of Pyridine- and Indazole-Based Inhibitors of the Inducible Nitric Oxide Synthase as Promising Antipsoriatic Agents
Source: ACS Pharmacol Transl Sci. 2026 Apr 22;9(5):1109–33. doi: 10.1021/acsptsci.5c00683 (PMC13162167; doi:10.1021/acsptsci.5c00683)
Supplement: Supplementary file 1 [file pt5c00683_si_001.pdf]

Supporting Information

Synthesis, biological evaluation and computational study of pyridine- and indazole-based inhibitors of the inducible Nitric Oxide Synthase as promising anti-psoriatic agents

Pasquale Amoia,[§] Marialucia Gallorini,[§,#] Claudia Scarponi,[†,#] Francisco Franco-Montalban,[‡,#] Patrizia Bonfanti,[¥] Anita Emilia Colombo,[¥] Valentina Di Francesco,[†] Stefania Madonna,[†] Alessandra Ammazzalorso,[§] Barbara De Filippis,[§] Letizia Giampietro,[§] Amelia Cataldi,[§] Rosa Amoroso,[§] Cristina Albanesi,[†*] Cristina Maccallini[§,*]*

[§]Department of Pharmacy, University "G.d'Annunzio" of Chieti-Pescara, Via dei Vestini 31, 66100 Chieti, Italy

[†] Laboratory of Experimental Immunology, Istituto Dermopatico dell'Immacolata, IDI-IRCCS, Via Monti di Creta 104, 00167, Rome, Italy

[‡] Department of Medicinal and Organic Chemistry, Faculty of Pharmacy, Campus Cartuja s/n, University of Granada, 18071, Granada, Spain

[¥] POLARIS Research Center, Department of Earth and Environmental Sciences, University of Milano-Bicocca, Piazza della Scienza 1, 20126, Milan, Italy.

[#] These Authors contributed equally to this work**;**

Corresponding Author *cristina.maccallini@unich.it; * cristina.albanesi@idi.it; *rosa.amoroso@unich.it

Table of Contents

HPLC analysis of the NOS assay……………………………………………………………S-3

Generation of nitric oxide in HaCaT cells under inflamed conditions in the presence of compound **10**…………………………………………………………………………..…………S-4

Figure S1…………………………………………………………………….………..…S-5

Figure S2……………………………………………………………………………...S-5

Molecular Dynamics analysis and Figures S3-S18……………………………………………..S-6

NMR spectra and HPLC trace for the target compounds……………………………...…S-25

Dose–response curves for iNOS inhibition by compounds **6**, **7** and **10**……………………………...………………………………………………….…S-35

Prediction of physicochemical and pharmacokinetic properties……………..………………...S-37

References……………………………………………………………………………..S-38

HPLC analysis of the NOS assay

The analysis were performed according to a previously reported method [1], with minor modifications. The o-phthalaldehyde-N-acetylcysteine (OPA/NAC) reagent for fluorescence derivatization of the NOS reaction mixtures was prepared with the molar ratios of 1:3, reacting 5 mL of methanolic OPA solution and 20 mL of 0.2 M borate buffer containing 0.1 g of NAC for 90 min to final pH 9.3 ± 0.05. The OPA/NAC solution was stored at 4 ◦C and saved for no longer than seven days. 600 μL of HPLC grade water was added to the residue of the enzymatic assay and centrifuged at 6000 rpm for 20 min. The fluorescence reaction is realized stirring 190 μL of supernatant and 60 μL of OPA/NAC solution for 5 min. HPLC analyses were performed using a Waters (Milford, MA, USA) system composed of a P600 model pump, a 2996 photodiode array detector, a 2475 multi-fluorescence detector, and a 7725i model sample injector (Rheodyne, Cotati, CA, USA). Chromatograms were recorded on a Fujitsu Siemens Esprimo computer and the Empower Pro software (Waters) processed data. The analyses were performed on an XTerra MS C8 column (250 × 4.6 mm id, 5 μm particle size) (Waters), equipped with an XTerra MS C8 guard column (Waters). A column thermostat oven mod-ule Igloo-Cil (Cil Cluzeau Info Labo, France) was used. The HPLC column was eluted at a flow rate of 0.7 mL/min with linear gradients of buffers A (5% CH3CN in 15 mM sodium borate with 0.1% v/v TFA, pH 9.4) and B (50% CH3CN in 8 mM sodium borate with 0.1% v/v TFA, pH 9.4). The solvent gradient was 0–20% B at 0–10 min, B to 25% at 10–15 min, then to 40% at 15–20 min and to 70% at 20–28 min. This composition was maintained until t = 35 min, before being reduced to the initial 0% B composition. The injection volume was 5 μL. The fluorescence intensity in the column eluate was monitored at 335 nm (excitation) and 439 nm (emission).

Generation of NO•

Nitrixyte™ probe (Cell Meter™ Fluorimetric Intracellular Nitric Oxide Assay Kit, AAT Bioquest, CA, USA) was used for detecting the production of free NO• in HaCaT cells by flow cytometry as reported previously [2]. The analysis was performed by using a CytoFLEX Flow Cytometer (Beckman Coulter, FL, USA) with a FL2 (PE) detector in linear mode. The experiment was performed in triplicate. Results are expressed as the mean value ± SD of the phycoerythrin peak of emission medians obtained analyzing samples with the CytExpert software (Beckman Coulter, FL, USA) and are provided to quantify the fluorescent changes in the FL2 channel.

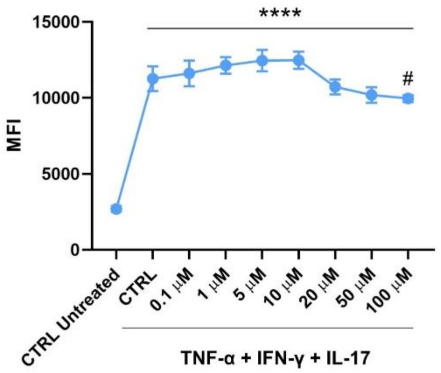

Figure S1. The trend line represents the effect of **8** on the intracellular nitric oxide generation after 24 h in HaCaT cells under basal (CTRL untreated = untreated cells) and under pro-inflammatory conditions (CTRL = cells stimulated with TNF-α, IFN-γ and IL-17) which is proportional to the MFI (mean fluorescence intensity) in the phycoerythrin channel. ****$p<0.0001$ between treatments and the untreated control; # $p<0.05$ between treatments and CTRL (cells stimulated with TNF-α, IFN-γ and IL-17).

A

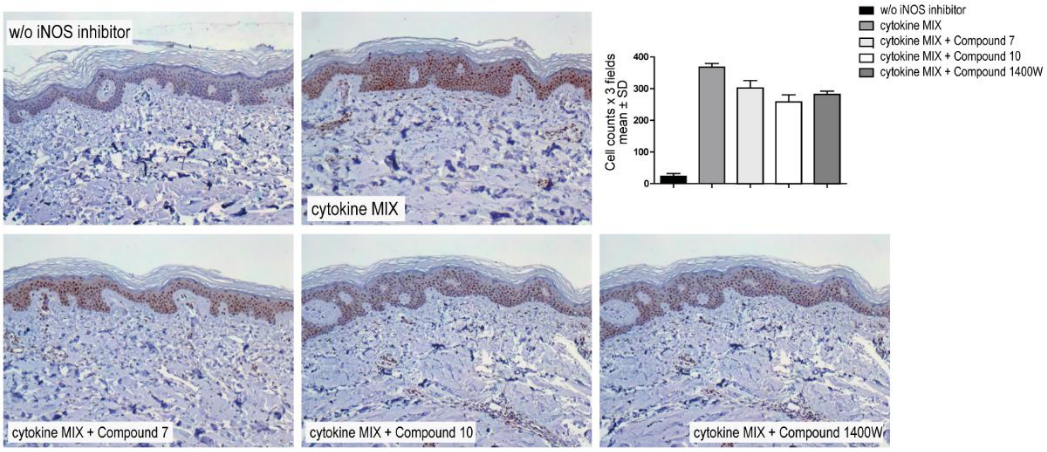

B

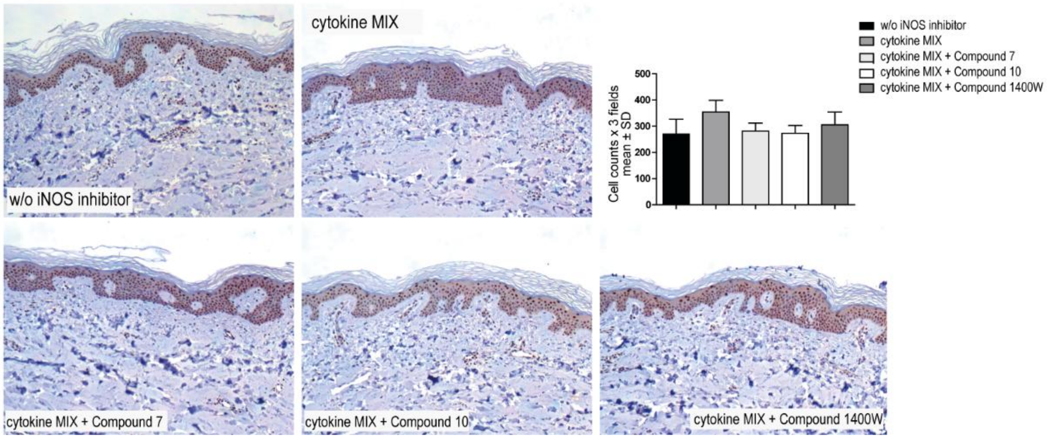

Figure S2. iNOS inhibitors slightly reduces A) STAT1 and B) STAT3 activation in the epidermis of skin explants mediated by IFN-γ, TNF-α and IL-17 cytokines. IHC analyses for p-STAT1 Tyr701 and p-STAT3 Tyr705 (stained in red/brown) were performed on paraffin-embedded sections of ex vivo skin grafts (n = 3), isolated from human healthy skin, treated with a combination of the cytokine mix with or without compounds **7**, **10** and 1400W. Sections were counterstained with Mayer's hematoxylin. Representative staining is shown. Graphs show the mean pixel intensity values ± SD. Analyses were performed in three different fields for each section. $p > 0.05$.

Molecular Dynamic analysis

To facilitate a direct comparison with the biochemical assays, molecular dynamics (MD) simulations were conducted using the same nitric oxide synthase (NOS) isoforms evaluated experimentally: human inducible NOS (hiNOS; PDB 4CX7) and bovine endothelial NOS (beNOS; PDB 3E7S). Starting with the docking poses of compound **10**, the proteins were modeled in their biologically relevant homodimeric states, with the docking pose of **10** occupying the active sites of both subunits (monomers A and B). The resulting complexes were analyzed separately for each monomer to capture potential subunit-dependent variations within the dimers. For each system, three independent replicas were simulated over trajectories extending to 50 ns.[3,4]

RMSD analysis

Root-mean-square deviation (RMSD) was used to assess structural stability of the protein scaffold and the positional stability of compound **10** during the 50 ns production trajectories. Protein RMSD was computed for backbone atoms after least-squares fitting to the corresponding initial structure. Subsequently, ligand RMSD was computed for ligand heavy atoms after fitting each frame to the backbone of the corresponding protein monomer and referenced to the initial bound pose for that monomer.

Backbone RMSD (dimer stability)

For both isoforms, the dimeric scaffold shows a rapid relaxation during the first few nanoseconds, followed by stable plateaus over the remainder of the simulation (Figure S3). The hiNOS dimer typically fluctuates within a narrow range (approximately 1.2–1.7 Å), with one replica showing a modest late increase toward ~1.8–1.9 Å after ~40 ns. The beNOS dimer reaches a slightly higher plateau (approximately 1.6–2.0 Å) but remains similarly stable thereafter. Overall, these RMSD magnitudes are consistent with preservation of the global fold over the simulated window and do not suggest large-scale destabilization that could explain differences in ligand behavior.

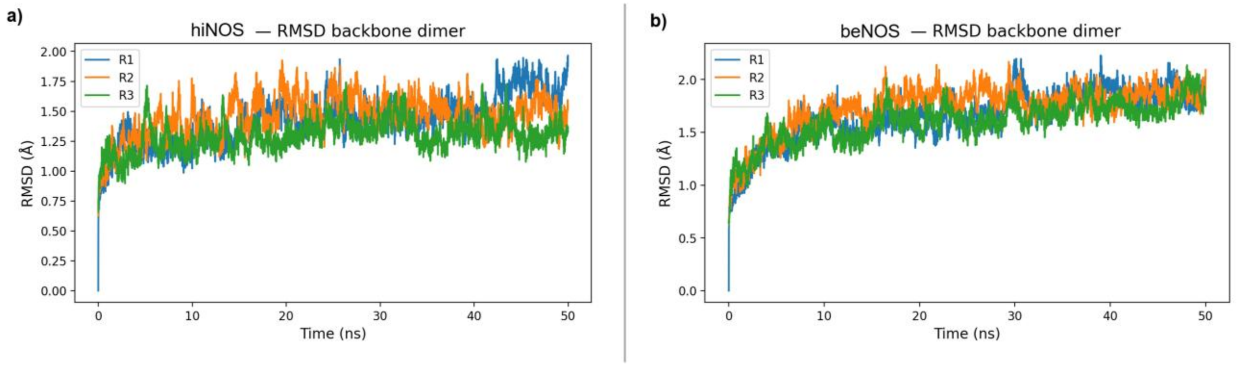

Figure S3. Backbone RMSD of the NOS dimers across three independent replicas (R1–R3). RMSD was computed for protein backbone atoms after least-squares fitting to the corresponding initial structure. (a) hiNOS (PDB 4CX7) dimer; (b) beNOS (PDB 3E7S) dimer.

Ligand RMSD (pose stability and reorganization)

Having confirmed the overall structural integrity of the dimeric assemblies, we next focused on the positional stability of compound **10** within the active sites. Compound **10** RMSD shows stronger monomer- and isoform-dependent heterogeneity than the protein backbone. In beNOS monomer A, the ligand undergoes an early adjustment and then remains in a moderate RMSD regime (roughly 1–2.5 Å), consistent with maintenance of a similar binding pose with local fluctuations (Figure S4a). In beNOS monomer B, RMSD increases more strongly and stabilizes at higher values (typically ~3–5 Å), indicating that the ligand explores alternative orientations or microstates within the pocket more extensively than in monomer A (Figure S4b).

In hiNOS, compound **10** displays broader rearrangements relative to the starting pose. In monomer A, ligand RMSD reaches high values (approximately 4–8 Å) with step-like transitions, implying that the initial docking geometry is not retained as a single rigid binding mode and that distinct bound arrangements are sampled over time (Figure S4c). In monomer B, RMSD is also elevated and replica dependent, with transitions to a higher-RMSD regime (typically ~4–5 Å) and occasional excursions, again consistent with multiple accessible poses (Figure S4d). Higher ligand RMSD values indicate a larger deviation from the initial pose and are consistent with increased mobility and/or reorientation of the ligand during the trajectory.

Taken together, these data indicate that both NOS dimers remain structurally stable across replicas, while the ligand samples an ensemble of bound configurations whose breadth depends on the monomeric context. Within the present simulations, hiNOS monomer A shows the highest ligand

RMSD values among the cases analyzed, consistent with a more pronounced rearrangement relative to the starting pose, whereas beNOS monomer A displays the lowest ligand RMSD and the most stable pose-like behavior.

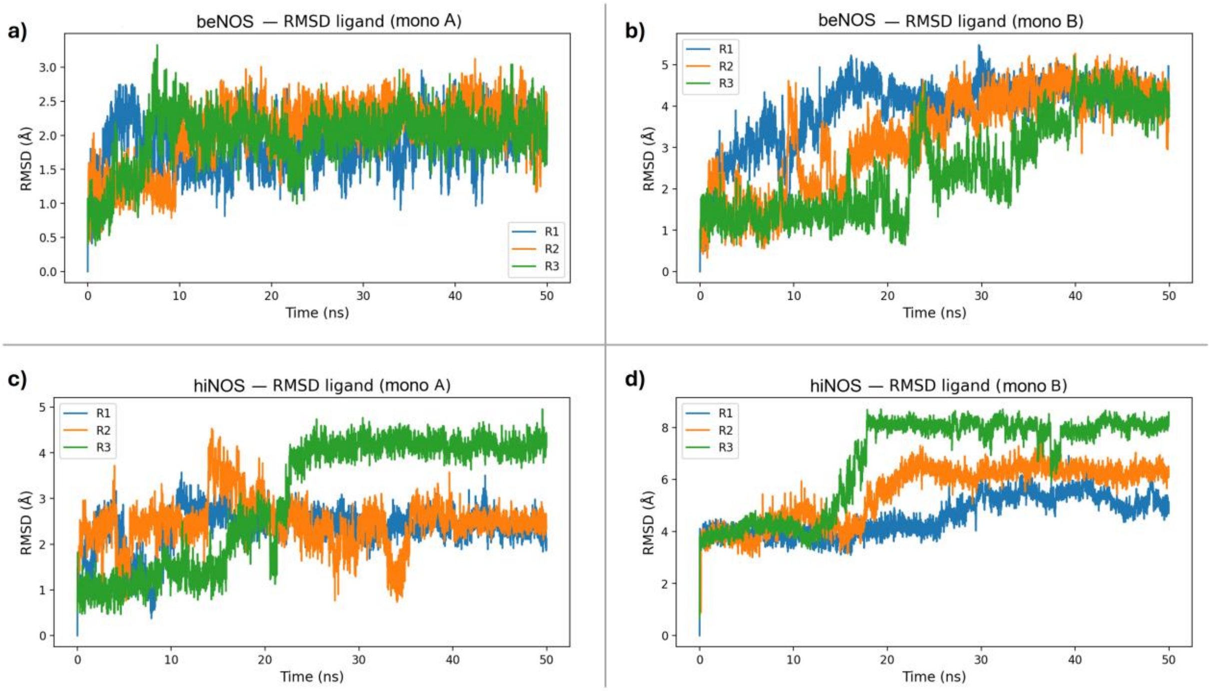

Figure S4. Ligand RMSD for compound **10** relative to the initial bound pose in each monomer. RMSD was computed for ligand heavy atoms after fitting each frame to the backbone of the corresponding monomer. (a) beNOS (PDB 3E7S) monomer A; (b) beNOS (PDB 3E7S) monomer B; (c) hiNOS (PDB 4CX7) monomer A; (d) hiNOS (PDB 4CX7) monomer B.

RMSF analysis and relationship to ligand-contact patterns

Backbone root-mean-square fluctuations (RMSF) were computed per residue after least-squares fitting of each trajectory to a common reference, and were compared between isoforms and between the two monomers within each dimer. Because RMSF reports the amplitude of positional fluctuations around the average structure, it highlights flexible segments and can help pinpoint regions that adapt during ligand binding. Here, we focus on isoform contrasts (hiNOS vs beNOS) for monomer A and monomer B, and summarize the residues that contribute most strongly to the differences using the top |ΔRMSF| ranking. For clarity and to preserve direct isoform comparability, RMSF is reported separately for monomer A and monomer B after structural alignment, and plotted as a function of equivalent residue position.

Across both proteins, the RMSF profiles show the expected pattern of a relatively rigid core with larger fluctuations at terminal regions and in a limited number of loop segments. In the isoform comparisons, most residues display similar fluctuation amplitudes, indicating broadly comparable backbone stability of the oxygenase-domain scaffold. The largest isoform-dependent differences are concentrated in discrete patches rather than distributed globally, consistent with local adaptations of the binding-site environment (Figures S5 and S6).

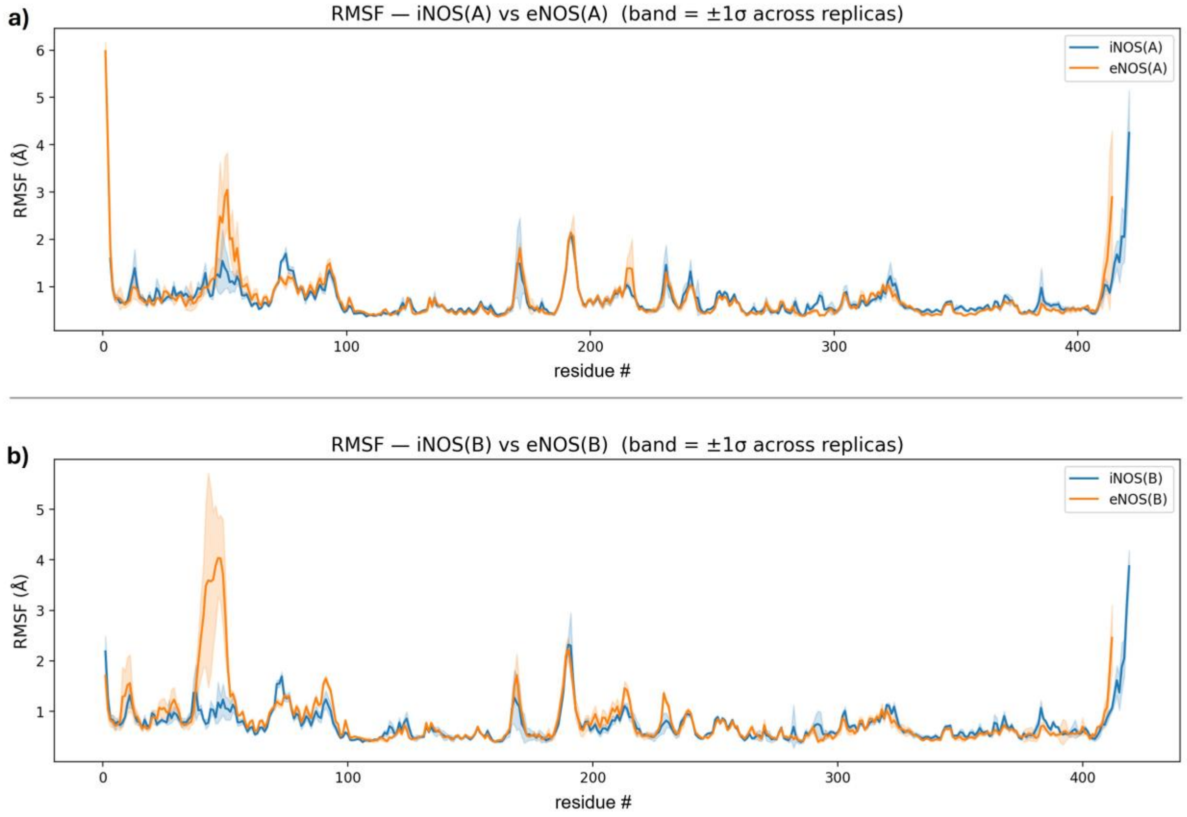

Figure S5. Backbone RMSF profiles (mean ± SD across replicas) for isoform comparisons. (a) hiNOS(A) vs beNOS(A). (b) hiNOS(B) vs beNOS(B). RMSF was computed after structural alignment and is plotted as a function of equivalent residue position; the solid line shows the replica-averaged RMSF and the shaded band indicates ±SD across R1–R3.

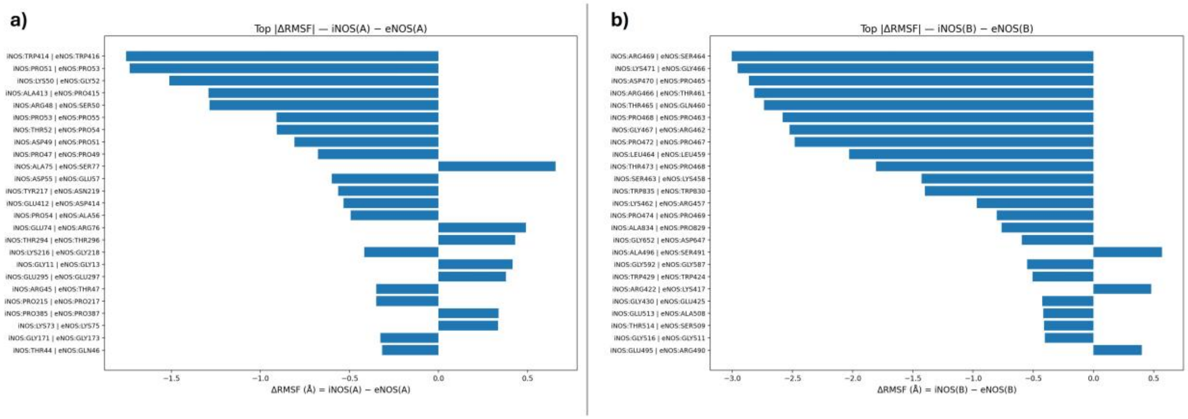

Figure S6. Residues contributing most strongly to isoform-dependent RMSF differences (top |ΔRMSF|). (a) hiNOS(A) − beNOS(A); (b) hiNOS(B) − beNOS(B). Bars report ΔRMSF (Å) at equivalent positions, annotated with the mapped residue identities in each isoform. Numbers of the residues correspond to the MD ensemble. For a complete residues hiNOS/beNOS number correspondence see Table Residues_equivalence in the SI.

Notably, several of the residues with the highest |ΔRMSF| between hiNOS(A) and beNOS(A) fall within regions that also show the strongest isoform-specific ligand contacts in the contact and hydrogen-bond analyses (see Figures 15-18 and section *Contacts and H-bonds analysis* below). In monomer A, prominent ΔRMSF contributions arise from an N-terminal segment around equivalent positions ~44–55 (and neighboring residues), as well as from pocket-lining segments around positions ~265–299 and ~383–385 (see Table Residues_equivalence for a residue mapping between isoforms and monomers). Within these regions, hiNOS shows markedly higher ligand contact and H-bond occupancy for residues such as Glu295 (377 in PDB 4CX7 numbering) and Arg299 (381 in PDB 4CX7 numbering), and strong contact enrichment at Pro385 (Pro467 in PDB 4CX7 numbering), whereas beNOS displays isoform-preferential contacts for residues including Val272, Phe289, Pro270, Trp292, and Tyr411 (Val335, Phe352, Pro333, Trp355 and Tyr474 in beNOS monomer A PDB 3E7S numbering). The coincidence between (i) residues that differ most in backbone mobility and (ii) residues that most consistently engage compound **10** supports the interpretation that the dominant isoform differences in RMSF are driven by local rearrangements within the binding pocket rather than by remote, unrelated flexibility.

The monomer B comparison shows a closely related picture. After accounting for the residue-number offset in the second subunit, the same equivalent pocket regions contribute strongly to

ΔRMSF and to isoform-specific ligand interactions, including the segment around equivalent positions ~292–301 (corresponding to hiNOS A residues 290-299 and their beNOS B counterparts 706-715) and the region around ~385-387 (corresponding to hiNOS A residues 383-385 and their beNOS B counterparts 799-801). These positions are among the most enriched in hiNOS contacts and H-bonds in monomer B (e.g., Glu716 (Glu295 in hiNOS A; Glu377 hiNOS (PDB 4CX7) numbering), Thr715 (Thr294 in hiNOS A; Thr376 hiNOS (PDB 4CX7) numbering) and Arg720 (Arg299 in hiNOS A ensemble; Arg381 hiNOS (PDB 4CX7) numbering), while beNOS shows preferential interactions in the corresponding region for residues such as Gln597 (Gln183 in beNOS A ensemble; Gln246 beNOS (PDB 3E7S) numbering), Asn688 (Asn274 in beNOS A; Asn337 beNOS mono A (PDB 3E7S) numbering), and Val686 (Val272 in beNOS A; Val335 beNOS mono A (PDB 3E7S) numbering).

Overall, the RMSF data confirm that the oxygenase domain remains structurally stable throughout the simulation. Significant mobility is restricted to specific regions near the binding pocket, varying between isoforms and monomers. These localized fluctuations provide the necessary context to interpret the global structural changes (Rg and SASA) discussed next. Furthermore, they set the stage for the binding analysis, helping to explain how the ligand adapts to a dynamic, flexible binding site.

Radius of gyration (Rg) analysis

The radius of gyration (Rg, Å) was calculated to quantify global compactness of the NOS dimers and to monitor conformational compactness of the bound compound **10** within each monomeric site during the 50 ns production simulations. Rg values were obtained with cpptraj[5] from the preprocessed trajectories for three independent replicas (R1–R3). For the protein, replica-wise time series were aggregated as the mean across replicas with the corresponding ±SD envelope (0.05 ns binning), consistent with the aggregation workflow used for other time-series metrics in this work.

Protein Rg (global compactness)

Protein Rg remains remarkably stable for both isoforms after the initial equilibration period (Figure S7), supporting preservation of the overall globular architecture across replicas. In beNOS, Rg increases rapidly from ~29.2–29.3 Å to ~29.5–29.6 Å during the first few nanoseconds and then fluctuates around a narrow plateau for the remainder of the trajectory, with only small-amplitude excursions and no persistent drift. In hiNOS, Rg is consistently higher (centered around ~30.2–

30.3 Å), shows a comparable early rise, and subsequently displays bounded fluctuations with modest replica dispersion. Overall, the limited variance (<~0.3 Å around the mean) and absence of long-term trends indicate that the dimers do not undergo major expansion/compaction transitions on the simulated timescale.

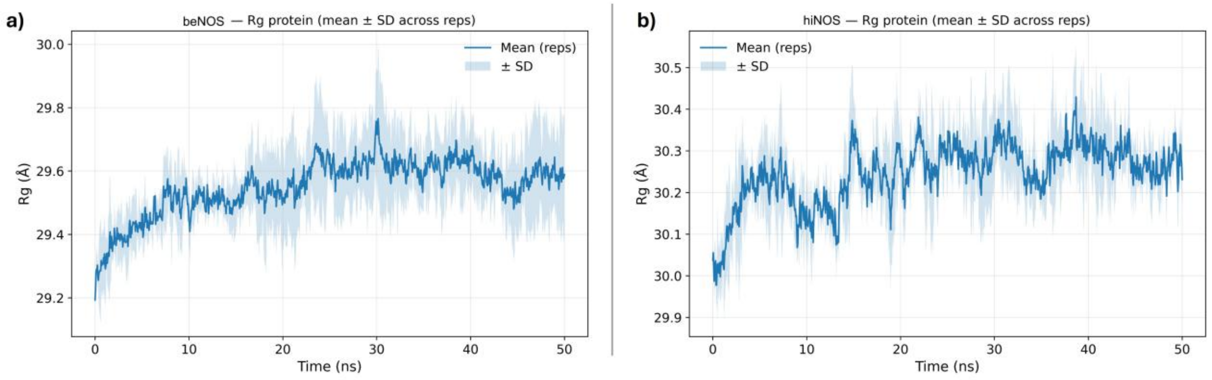

Figure S7. Protein radius of gyration (Rg; mean ± SD across replicas) along 50 ns production MD. (a) beNOS; (b) hiNOS. The solid line shows the replica-averaged Rg and the shaded band indicates ±SD across R1–R3.

Ligand Rg (compound 10 conformational compactness)

Ligand Rg reports on the compactness of compound **10** itself and provides a complementary view of intra-pocket conformational flexibility (Figure S8). Across systems, compound **10** samples Rg values in the ~4.2–5.2 Å range, indicative of interconversion between more compact and more extended conformations. In hiNOS, monomer B exhibits the most pronounced replica heterogeneity, with trajectories switching between compact states (Rg ≈ 4.1–4.4 Å) and more extended conformers (Rg ≈ 5.0–5.2 Å), whereas monomer A remains comparatively centered near ~4.8–5.1 Å with transient compaction events. In beNOS, ligand Rg is broadly stable in monomer A, while monomer B displays more noticeable shifts, including late-stage compaction in one replica. Together, these profiles suggest that compound **10** undergoes monomer- and isoform-dependent intramolecular rearrangements while the protein scaffold remains globally stable.

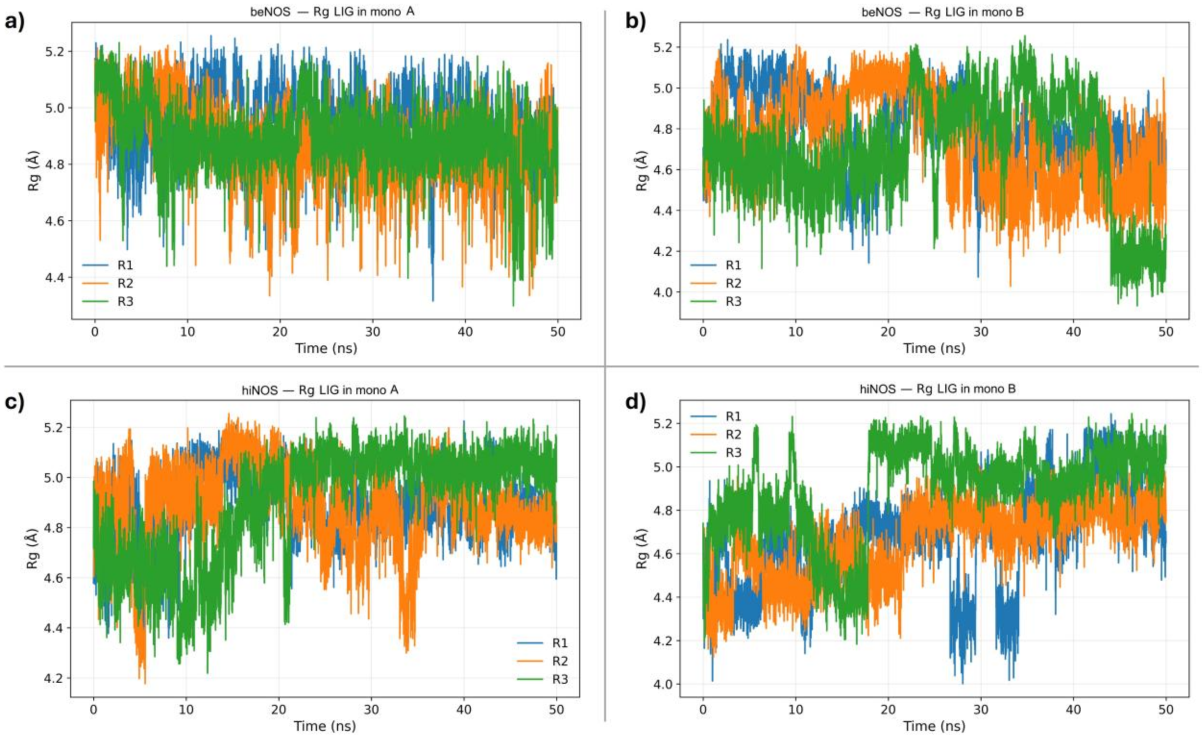

Figure S8. Radius of gyration (Rg) of compound 10 within each monomeric site across three independent replicas (R1–R3). (a) beNOS monomer A; (b) beNOS monomer B; (c) hiNOS monomer A; (d) hiNOS monomer B.

SASA analysis

Solvent accessible surface area (SASA) was used to monitor global compactness of the NOS homodimer and to quantify the solvent exposure of the bound compound **10** within each active site throughout the production trajectories. SASA ($Å^2$) was computed with cpptraj[3] on the preprocessed, imaged and stripped trajectories for three independent replicas (R1–R3) per system. To facilitate replica-wise comparisons, time series were aggregated as the mean across replicas with the corresponding ±SD envelope (binned at 0.05 ns).

Protein SASA (global compactness)

For both isoforms, the protein SASA shows an initial relaxation phase followed by a stable regime without sustained drift (Figure S9). In beNOS (PDB 3E7S), SASA rises rapidly during the first few nanoseconds and subsequently fluctuates around a near-stationary plateau, consistent with routine "breathing" motions of the dimer rather than large-scale expansion or unfolding. In hiNOS

(PDB 4CX7), a comparable early increase is observed, including a short-lived compaction event around ~8–10 ns, after which SASA remains broadly stable for the remainder of the trajectory. Overall, the magnitude and stationarity of the SASA fluctuations support that both dimers maintain their global integrity over 50 ns.

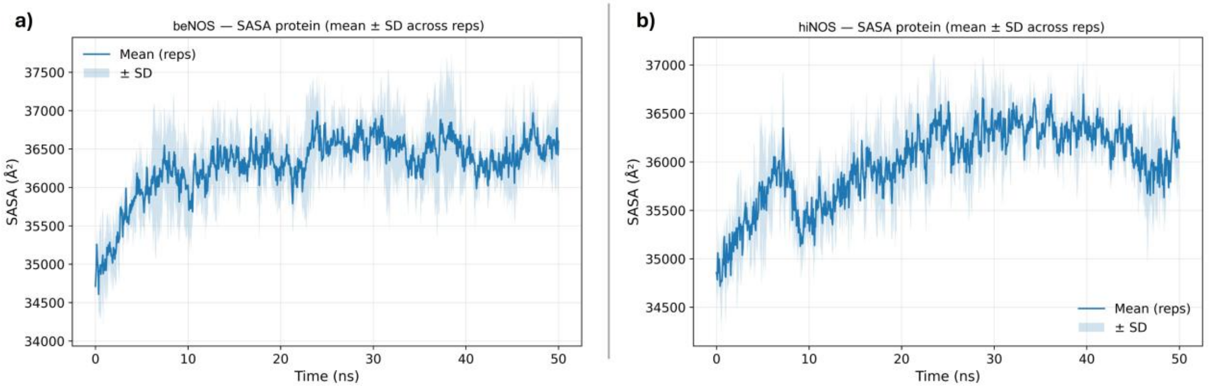

Figure S9. Protein SASA (mean ± SD across replicas) along 50 ns production MD. (a) beNOS (PDB 3E7S); (b) hiNOS (PDB 4CX7). The solid line shows the replica-averaged SASA and the shaded band indicates ±SD across R1–R3.

Ligand SASA and monomer asymmetry

Ligand SASA provides a direct measure of compound **10** burial within each binding pocket and reveals pronounced monomer-dependent behavior (Figure S10). In beNOS, compound **10** becomes progressively less solvent-exposed in monomer B, consistent with increased burial of the ligand within that pocket over time. In contrast, monomer A displays an opposite trend with increasing ligand exposure, suggesting intermittent opening at the binding-site entrance or a gradual shift toward a more solvent-accessible pose. In hiNOS, the mean ligand SASA remains more moderate overall; however, monomer B exhibits marked replica heterogeneity (increasing SD after the initial equilibration), consistent with alternating burial/exposure substates across replicas, while monomer A shows a smaller net change and comparatively steadier dispersion.

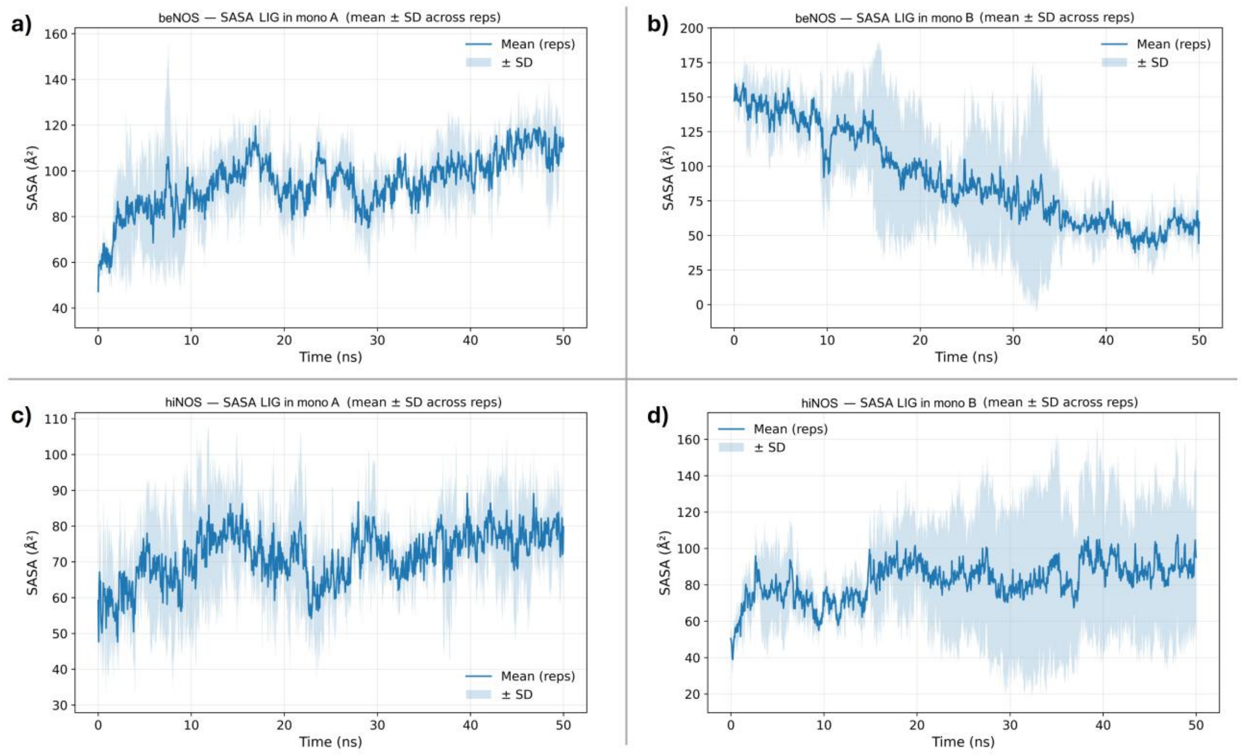

Figure S10. SASA of compound 10 within each monomeric site across three independent replicas (R1–R3) (mean ± SD across replicas). (a) beNOS monomer A; (b) beNOS monomer B; (c) hiNOS monomer A; (d) hiNOS monomer B.

Cluster analysis and representative binding modes

Binding-mode diversity was evaluated by clustering the ligand–pocket configurations using a simple two-state model (K = 2), aimed at capturing the two most recurrent binding families while keeping the analysis directly comparable across isoforms and monomers. A preliminary sweep of K values (2–8) using standard internal validation indices (Davies–Bouldin, mean silhouette and pseudo-F) indicated that the “best” K can vary depending on the metric and the system. For example, hiNOS monomer A shows its strongest support around K = 3 in all three indices, whereas for beNOS monomer A the indices do not converge on a single optimum (pseudo-F favors K = 2, silhouette peaks near K = 3, and Davies–Bouldin improves up to larger K before degrading). In practice, increasing K mainly subdivides the dominant family into smaller subclusters rather than revealing additional well-populated, clearly separated binding modes. For this reason, K = 2 was retained as a conservative and interpretable partition into two dominant families, suitable for robust downstream ensemble sampling.[6-8]

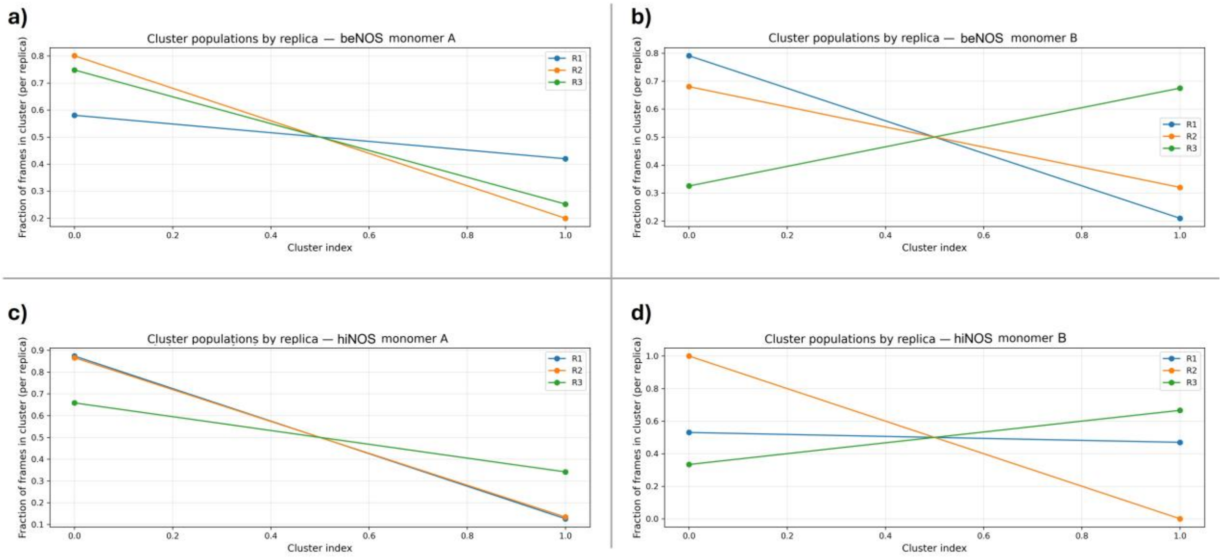

Figure S11. Cluster populations by replica for beNOS (3E7S) and hiNOS (4CX7) monomers under a two-state binding-mode model (K = 2). Panels: a), beNOS monomer A; b), beNOS monomer B; c), hiNOS monomer A; d), hiNOS monomer B. For each independent replica, the y-axis reports the fraction of trajectory frames assigned to cluster 0 or cluster 1 (cluster labels are arbitrary and do not imply a rank).

Across both isoforms, monomer A shows a relatively consistent clustering profile across replicas, with one cluster remaining dominant and the second contributing a smaller fraction of frames (Figure S11). In beNOS monomer A, replica 1 is more mixed than replicas 2–3, whereas in hiNOS monomer A the dominant cluster is strongly populated in replicas 1–2 and remains prevalent in replica 3. In contrast, monomer B displays larger replica-to-replica shifts in cluster occupancy in both isoforms. In beNOS monomer B, replicas 1–2 preferentially populate cluster 0 while replica 3 predominantly samples cluster 1; a similar pattern is observed for hiNOS monomer B, where replica 2 remains almost entirely in cluster 0, replica 3 shifts toward cluster 1, and replica 1 samples both clusters to a comparable extent. Overall, these profiles support the presence of at least two recurrent ligand arrangements in the binding site and suggest that their relative populations can remain sensitive to replica-specific sampling over the 50 ns trajectories, particularly for monomer B.

To visualize the two dominant pose families identified by the K = 2 clustering described above, representative structures (cluster centroids) were extracted separately for each isoform and

monomer within the dimer. For each monomer in hiNOS (PDB 4CX7) and beNOS (PDB 3E7S), representative structures (cluster centroids) were extracted for cluster 0 and cluster 1, yielding a total of eight representative PDB snapshots (2 clusters × 2 monomers × 2 isoforms). The superposition of the two cluster representatives within each monomer highlights the main pose families sampled by ligand **10** (Figures S12–S13).

hiNOS (4CX7)

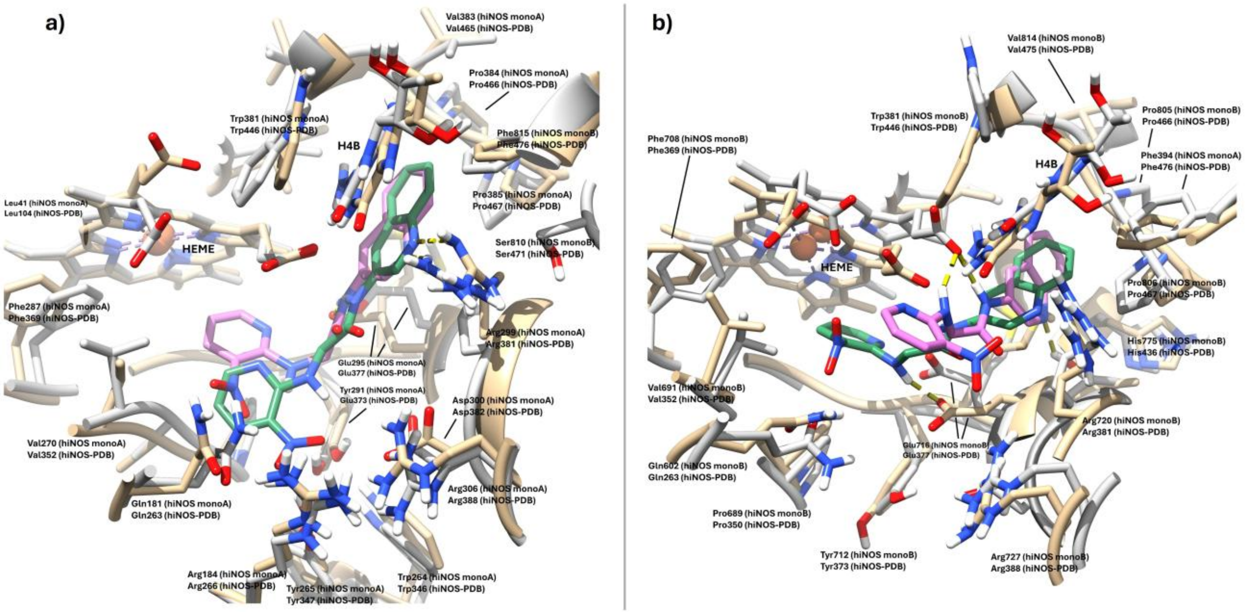

Figure S12. Representative cluster structures for hiNOS (PDB 4CX7) obtained from K = 2 clustering: (a) monomer A and (b) monomer B. In each panel, the protein is shown in tan with compound **10** in green for cluster 0, and protein in grey with compound **10** in magenta for cluster 1.

In hiNOS monomer A (Figure S12a), compound **10** predominantly occupies the substrate-binding region associated with L-arginine, positioning polar groups toward key catalytic residues Glu295 (Glu377 in PDB numbering), Arg299 (Arg381 in PDB numbering), and Tyr265 (Tyr347 in PDB numbering), consistent with recurrent hydrogen-bonding interactions across the clustered representatives (vide infra). A distinctive feature of the hiNOS binding mode involves the orientation of the 3-quinoline bicyclic moiety, which inserts into a pocket between the H4B (tetrahydrobiopterin) cofactor and surrounding residues. In particular, this aromatic system packs against Phe394 (Phe476 in PDB numbering) from monomer B (ensemble residue 815) and is further stabilized by contacts with Pro384 (Pro466 in PDB 4CX7 numbering) and Pro385 (Pro467

in PDB 4CX7 numbering). Notably, a comparable pose family is observed for monomer B, indicating that the two monomers sample closely related ligand orientations in this isoform (Figure S12b).

beNOS (3E7S)

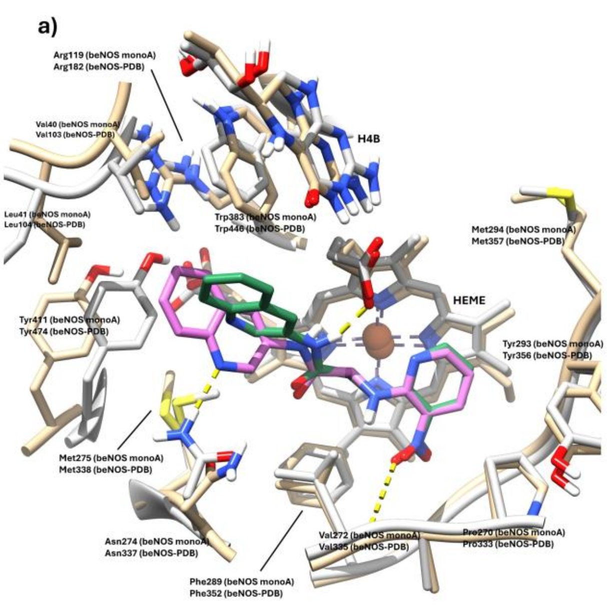

Figure S13. Representative cluster structures for beNOS (PDB 3E7S) obtained from K = 2 clustering: (a) monomer A and (b) monomer B. In each panel, the protein is shown in tan with compound **10** in green for cluster 0, and protein in grey with compound **10** in magenta for cluster 1.

In contrast, the beNOS representatives reveal an alternative binding disposition relative to hiNOS. In this isoform, compound **10** is shifted away from the canonical L-arginine region toward an opposing pocket, where the 3-quinoline ring is primarily stabilized through hydrophobic contacts with residues such as Tyr411 (Tyr474 in PDB numbering), Leu41 (Leu104 in PDB numbering), Val272 (Val335 in PDB numbering), Trp383 (Trp446 in PDB numbering), and Phe289 (Phe352 in PDB numbering), among others (Figure S13a–b). In agreement with this more hydrophobic environment, the number of persistent hydrogen bonds is reduced compared with hiNOS, and when present they involve residues including Val272 (Val335 in PDB numbering), Tyr411 (Tyr474 in PDB numbering), Asn274 (Asn337 in PDB numbering), Arg600 (Arg249 in PDB numbering) and Gln597 (Gln246 in PDB numbering).

Overall, the K = 2 clustering highlights isoform-dependent pose families for compound **10**. The preferential localization of the ligand in the substrate-associated region in hiNOS, together with a distinct packing of the 3-quinoline moiety near the H4B cofactor, contrasts with the alternative, more distant pocket sampled in beNOS. This divergence in binding mode provides a plausible structural rationale for the in vitro selectivity reported for hiNOS relative to beNOS isoforms.

While clustering highlights recurrent pose families and isoform-dependent interaction patterns, such structural observations do not by themselves provide a quantitative estimate of relative stabilization. We therefore complemented the pose analysis with an approximate end-state energetic descriptor (MM/GBSA), used here to compare the relative stabilization of the dominant bound ensembles across isoforms and monomers. Because MM/GBSA estimates can be sensitive to conformational sampling and to the representation of distinct pose families, the energy evaluation was performed on a cluster-balanced set of representative frames.

To reduce the risk that energetic estimates would be dominated by a single, overrepresented binding arrangement in a given replica, representative snapshots were selected in a cluster-balanced manner (20 snapshots per cluster; 40 snapshots per case). This ensures that both recurrent binding modes contribute comparably to the MM/GBSA evaluation, even when one mode is preferentially sampled in a specific replica. MM/GBSA energies were therefore computed from representative frames drawn from both dominant clusters, so that the recurrent binding arrangements observed in the trajectories are consistently reflected in the energetic analysis.[9-10]

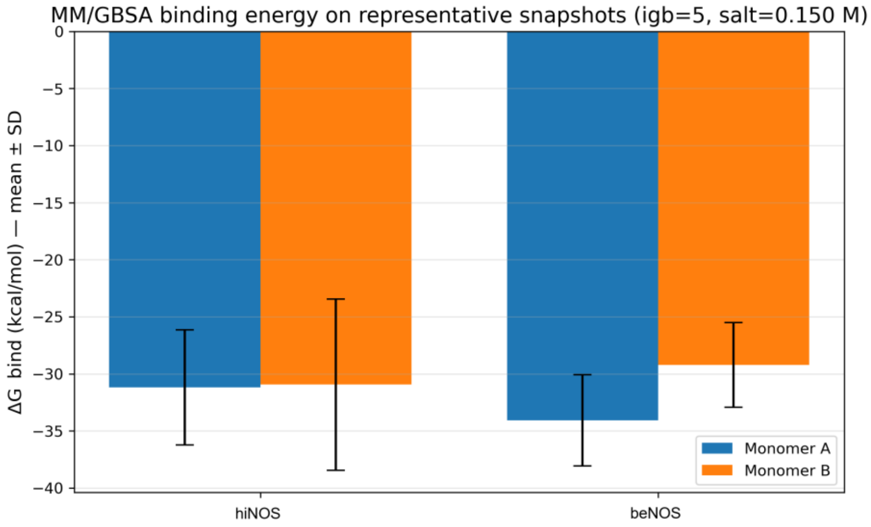

Figure S14. MM/GBSA binding energy (GB model) estimated on representative snapshots. ΔG bind values were computed with an implicit-solvent GB model (igb = 5) at 0.150 M salt on 40 representative snapshots per case. Error bars correspond to the dispersion across the selected snapshots (as plotted). Because snapshots were selected as 20+20 from the two binding-mode clusters, these values represent an equal-weight mixture of the two clustered binding arrangements rather than a population-weighted estimate.

Across the four monomer-specific cases, the MM/GBSA results show a clear dependence on subunit context. For monomer A, beNOS exhibits a more favorable mean binding energy than hiNOS, whereas for monomer B the trend is reversed, with beNOS appearing less favorable than hiNOS. At the same time, the snapshot-to-snapshot variability is appreciable—most notably for hiNOS monomer B—so the separation between mean values should be interpreted cautiously rather than as a definitive energetic ranking. Taken together, the simulations are consistent with stable ligand binding in both isoforms, while any apparent energetic preference depends on the monomeric environment within the dimer and on the coexistence of two recurrent binding arrangements sampled over the trajectories.

Contacts and H-bonds analysis

Hydrogen-bond analyses indicate that polar engagement differs between isoforms. The beNOS simulations show generally low hydrogen-bond counts in both monomers, most often fluctuating around 0–1 with sporadic short-lived excursions to 2–3 (Figure S15). In contrast, the hiNOS simulations exhibit more frequent hydrogen bonding, typically sampling 1–3 hydrogen bonds with occasional transient increases (up to ~5 in monomer A and ~4 in monomer B). While replica-dependent fluctuations are evident in all panels, the overall pattern suggests that the ligand forms a more recurrent polar contact network in hiNOS than in beNOS within the simulated time window. Importantly, hydrogen-bond counts alone do not provide a direct proxy for binding affinity, as favorable binding can also arise from hydrophobic packing, π-stacking, and other noncovalent contributions captured in the overall interaction ensemble.

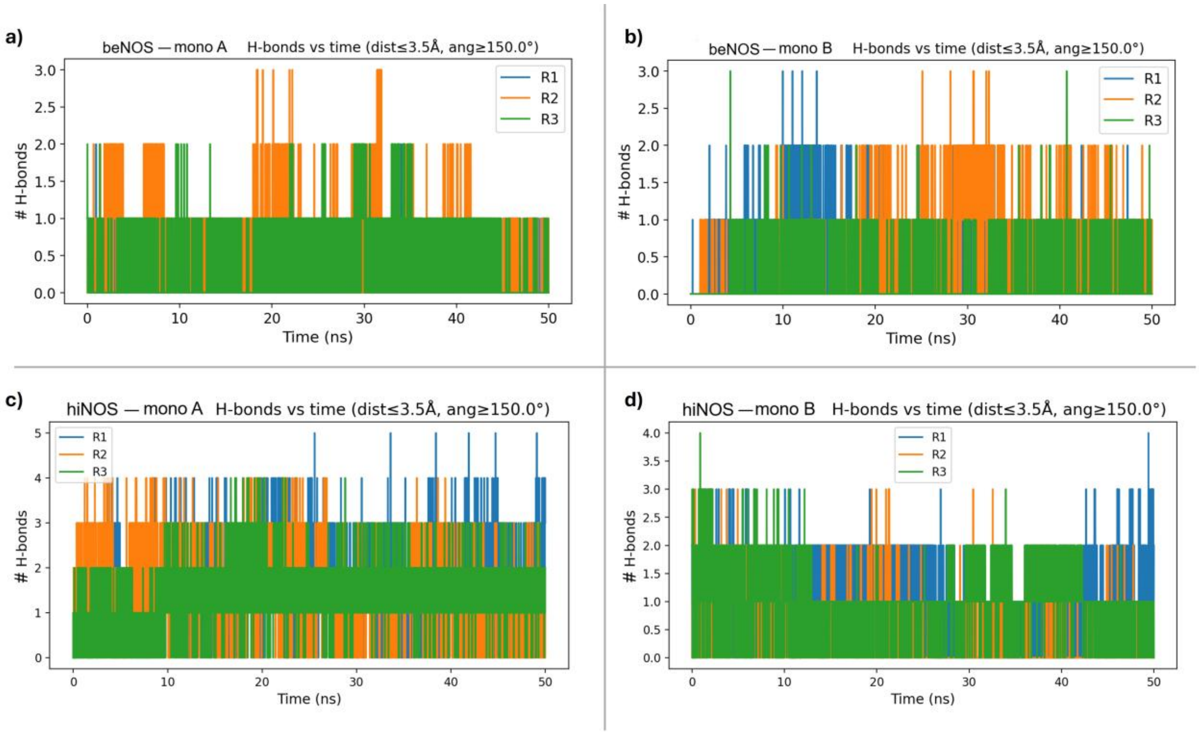

Figure S15. Hydrogen-bond counts over time for beNOS and hiNOS (monomers A and B; three replicas each). Hydrogen bonds were counted using a geometric criterion (distance ≤ 3.5 Å and angle ≥ 150°). Each colored trace corresponds to one independent replica. Panels: (a) beNOS monomer A; (b) beNOS monomer B; (c) hiNOS monomer A; (d) hiNOS monomer B.

Per-residue hydrogen-bond occupancies further support distinct polar anchoring patterns across isoforms and subunits (Figure S16). In beNOS, the most frequent hydrogen-bonding partners show only moderate occupancies, with Val272 (Val686 in monomer B) emerging as the dominant contributor in monomer A and Gln597 (Gln183 in monomer A) in monomer B, accompanied by smaller contributions from residues such as Tyr411/Asn274 (monomer A) and Asn688/Val686 (monomer B). In hiNOS, hydrogen bonding is instead concentrated in a smaller set of residues with higher persistence: Glu295 (Glu716 in monomer B) and Arg299 (Arg720 in monomer B) (with an additional contribution from Tyr265 (Tyr686 in monomer B) dominate in monomer A, whereas Glu716 together with Thr715 and Arg720 are the main contributors in monomer B. These isoform- and subunit-specific occupancy patterns indicate that the ligand can be stabilized by different polar microenvironments in each NOS structure, with hiNOS providing more persistent, residue-localized hydrogen-bond anchors than beNOS in the present simulations.

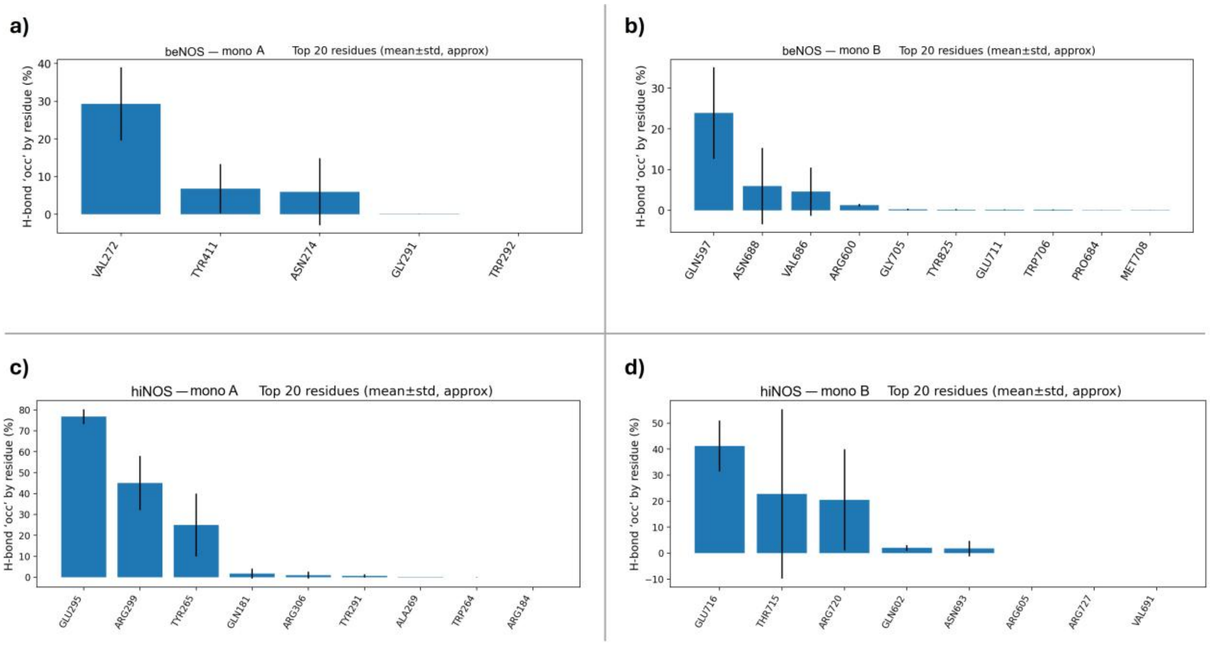

Figure S16. Major hydrogen-bond partners identified from per-residue hydrogen-bond occupancies (approximate). Bars summarize the approximate percentage of frames in which a given residue participates in at least one hydrogen bond with the ligand, reported as mean ± standard deviation across replicas (as plotted). Residue names and numbering correspond to each structure. For a complete hiNOS/beNOS residues number correspondence see Table S1 Residues_equivalence.

Contact analyses support the conclusion that the ligand remains engaged with the binding site in all simulations, while still allowing for conformational rearrangements and replica-dependent differences (Figure S17). The number of contacting residues fluctuates over time and can shift between plateaus, consistent with local reorganization within the pocket and/or transitions between the two clustered binding arrangements. Notably, a comparative assessment reveals that the ligand maintains a slightly higher average number of contacts in hiNOS compared to beNOS. This denser contact network is consistent with more persistent engagement of compound **10** in the human isoform and could contribute to the experimentally observed preference.

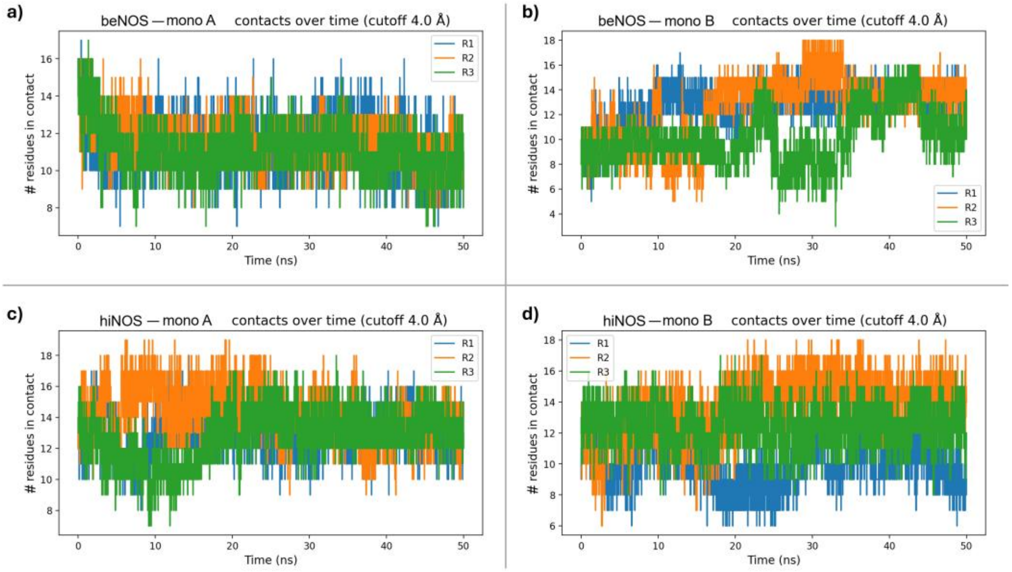

Figure S17. Time evolution of the number of protein residues contacting the ligand (4.0 Å cutoff). A residue is counted as in contact when any heavy-atom distance between the residue and the ligand is ≤ 4.0 Å. Each trace corresponds to one replica.

Figure S18 summarizes the residues that most frequently remain in close contact with compound **10** during the simulations. The contact patterns differ between isoforms, in agreement with the representative binding modes obtained from clustering. In hiNOS, the dominant contacts in monomer A include residues from the substrate-associated region, such as Glu295, Arg299, and Tyr265, together with Pro384/Pro385, consistent with the clustered poses in which compound 10 occupies the L-arginine/H4B-associated environment and the 3-quinoline moiety is accommodated in that region. In beNOS, the dominant contacts shift toward a more hydrophobic set characteristic of the alternative pocket observed in the clustered representatives, including Tyr411, Leu41, Val272, Trp383, and Phe289 in monomer A, and contacts involving Gln597 and Arg600 in monomer B.

Overall, the residue fingerprints in Figure S16 support the view that compound **10** engages different contact networks in hiNOS and beNOS, consistent with isoform-dependent binding arrangements. While contact occupancies alone do not quantify selectivity, the observed differences are compatible with the distinct pose families described by clustering.

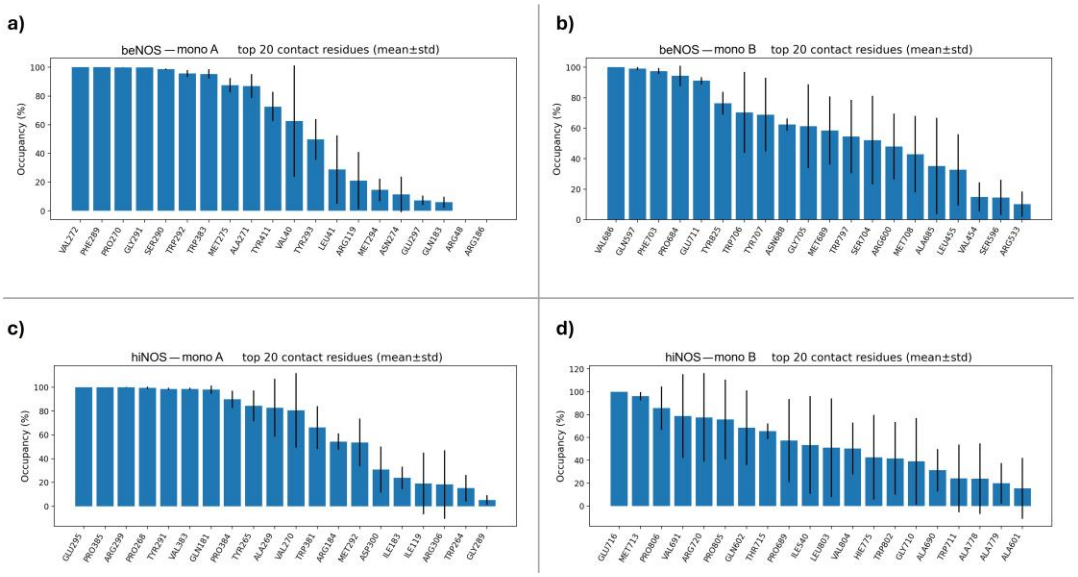

Figure S18. Dominant contact residues (mean ± SD across replicas; 4.0 Å cutoff). Bars report the occupancy of contact (≤ 4.0 Å) for the most frequently contacting residues, shown as mean ± SD across replicas. Large error bars indicate contact persistence that varies substantially between replicas. Numbers of the residues correspond to the MD ensemble. For a complete hiNOS/beNOS residues number correspondence see Table S1 Residues_equivalence.

Collectively, the simulations suggest that compound **10** remains associated with both NOS dimers during the simulated time window, but it tends to adopt different binding arrangements in each isoform and can behave differently in each monomer. The energy estimates used here do not give a single, clear ordering that matches the experimental selectivity, so they are best taken as supportive trends rather than a definitive answer. The clearest difference comes from the binding pattern: in hiNOS, the ligand more often remains near the substrate/H4B region and keeps a more consistent set of polar contacts, whereas in beNOS the 3-quinoline ring more often favors a more distant, more hydrophobic pocket and the polar contacts are less continuously maintained. These isoform-dependent differences are consistent with prior structural work showing that changes in the mobility of conserved residues can shape how inhibitors are accommodated in different NOS isoforms.[3]

Residue equivalence table

See file: *Residues_equivalence.xlsx* **Table S1.** Residue equivalence mapping between hiNOS (4CX7) and beNOS (3E7S) across ensemble numbering, PDB numbering, and monomer offsets.

NMR spectra and HPLC trace for the target compounds

Compound 6

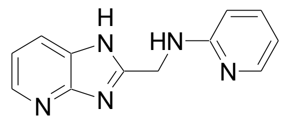

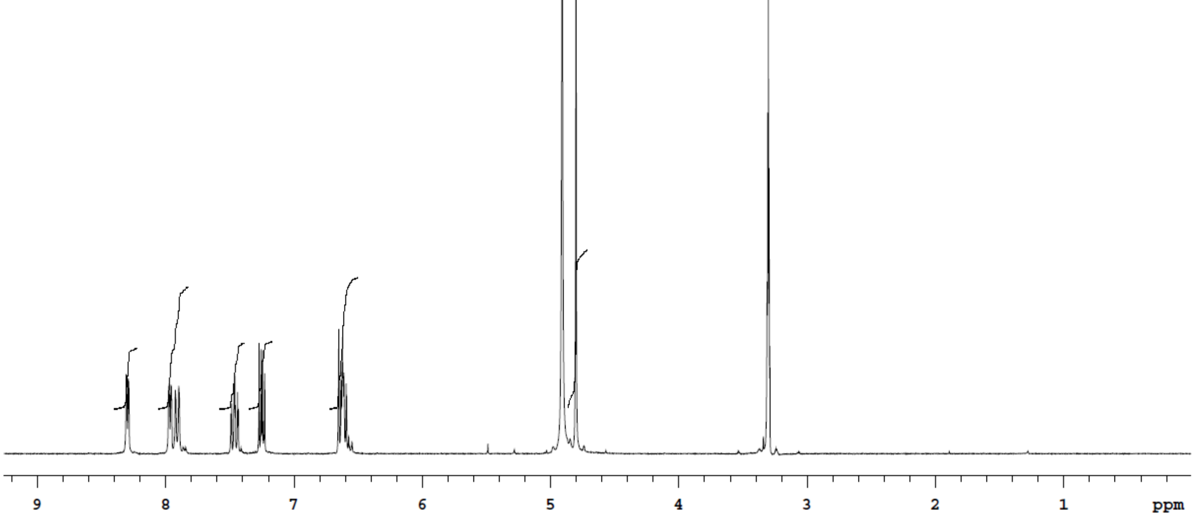

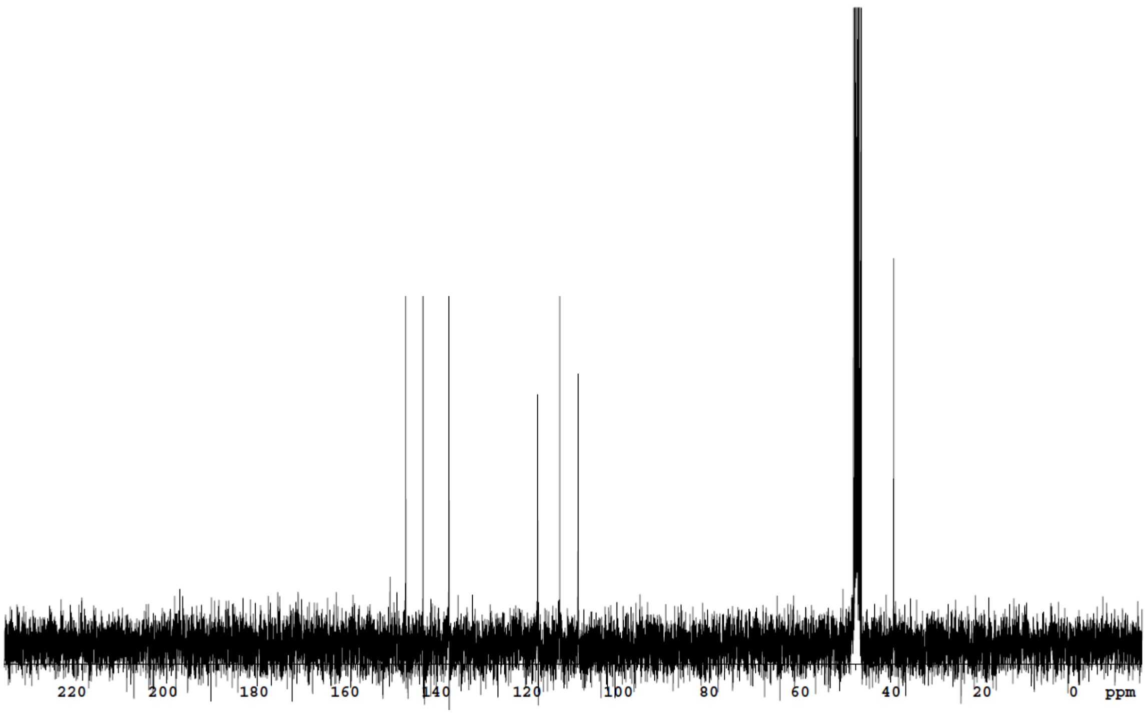

Compound **7**

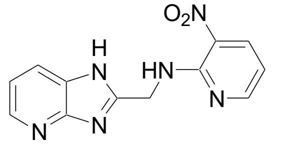
O2N
H
N
HN
N
N
N

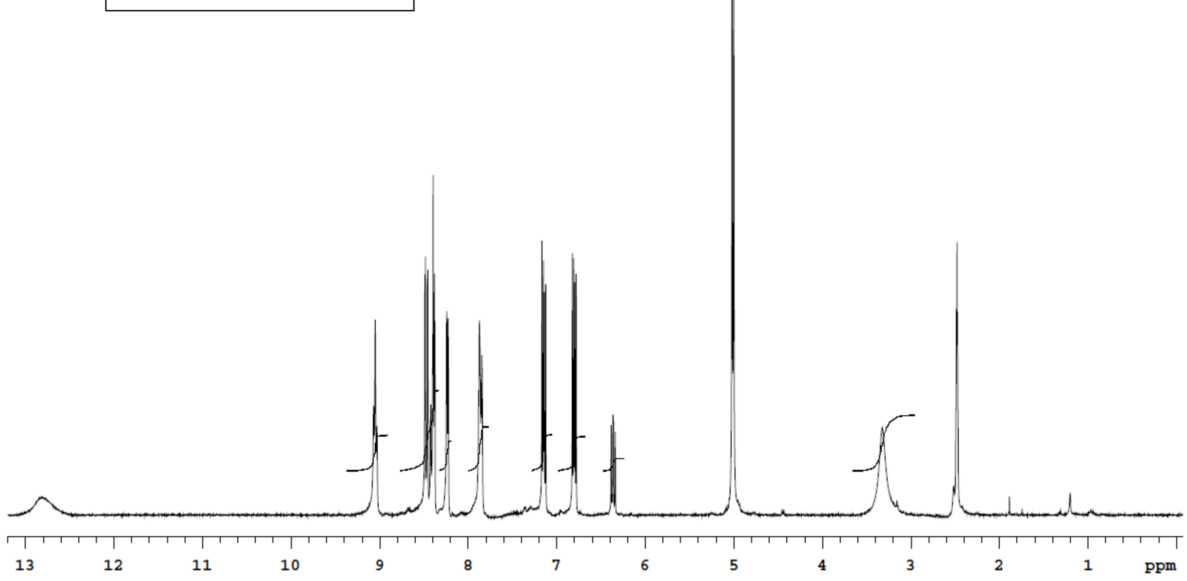
13 12 11 10 9 8 7 6 5 4 3 2 1 ppm

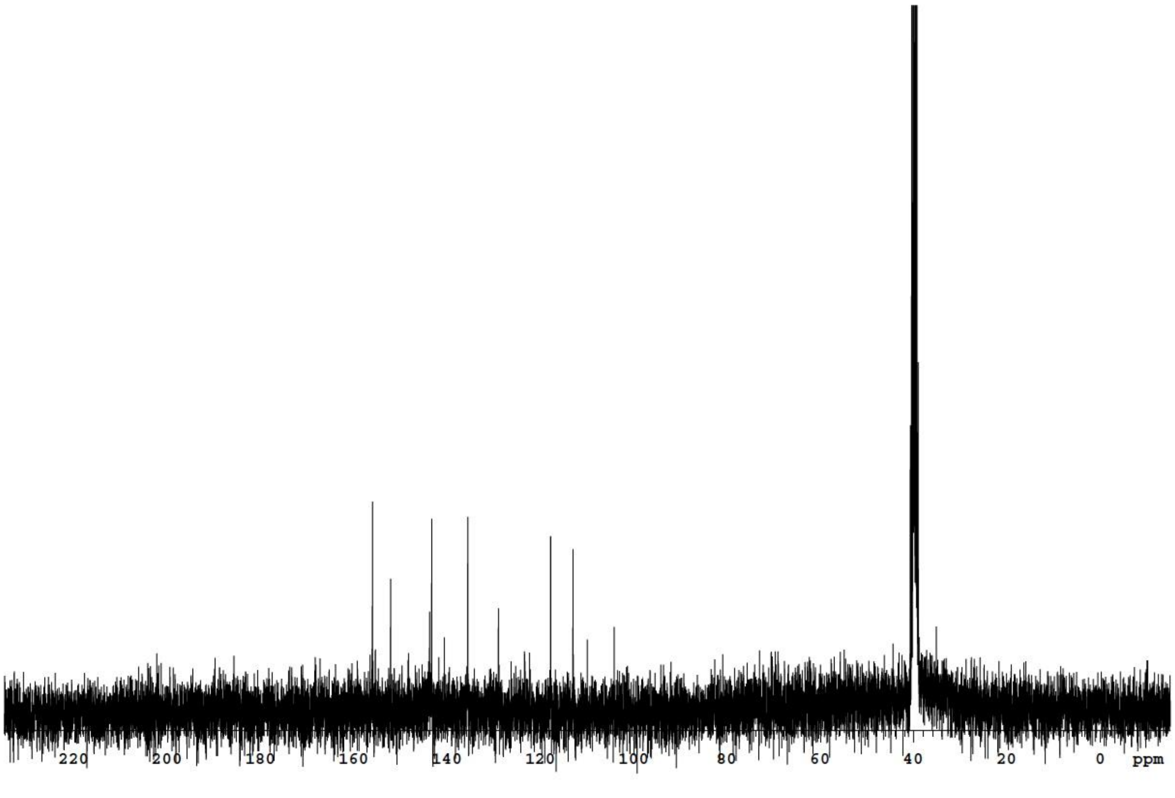
220 200 180 160 140 120 100 80 60 40 20 0 ppm

Compound **10**

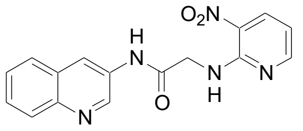

O_2N
H
N
N
N
H
O
N

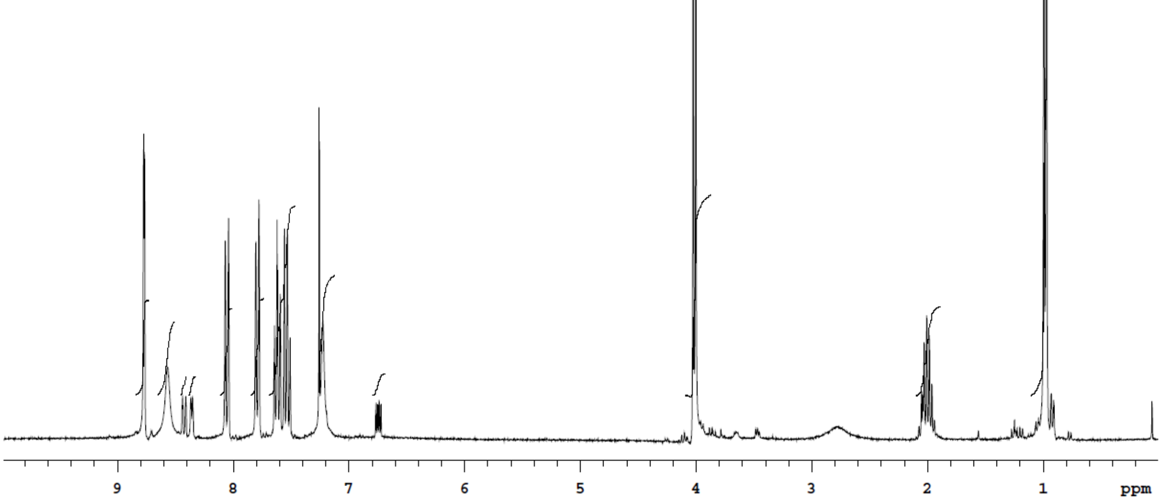

9
8
7
6
5
4
3
2
1
ppm

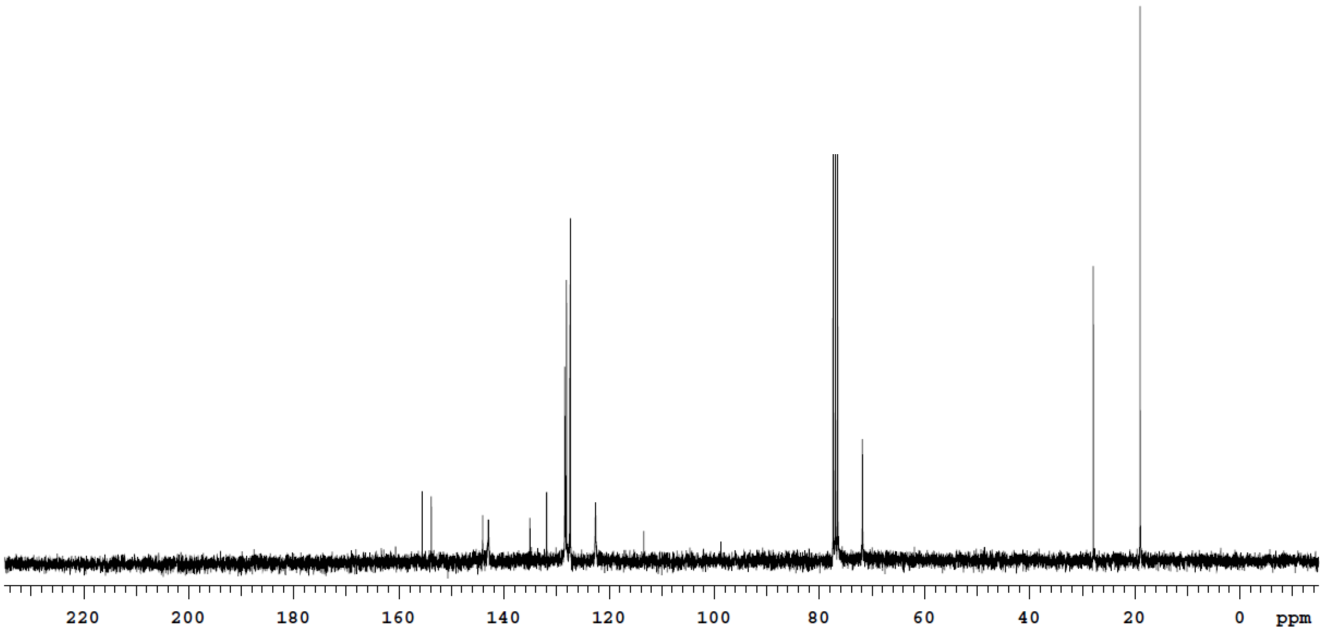

220
200
180
160
140
120
100
80
60
40
20
0
ppm

Compound **12**

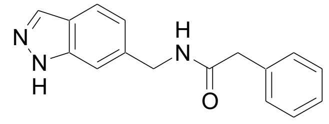

N
N
H
H
N
O

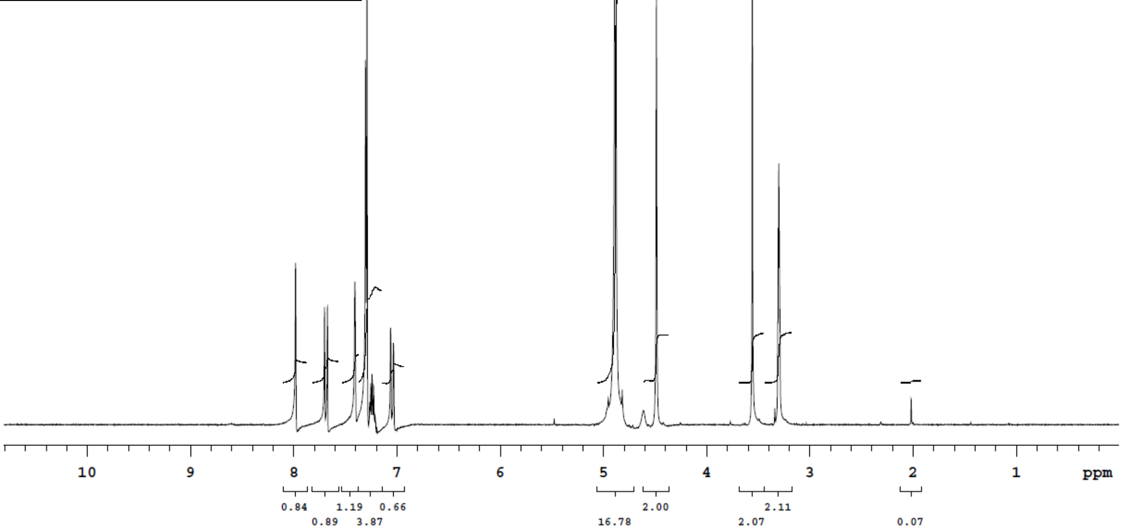

10 9 8 7 6 5 4 3 2 1 ppm
0.84
0.89
1.19
3.87
0.66
16.78
2.00
2.07
2.11
0.07

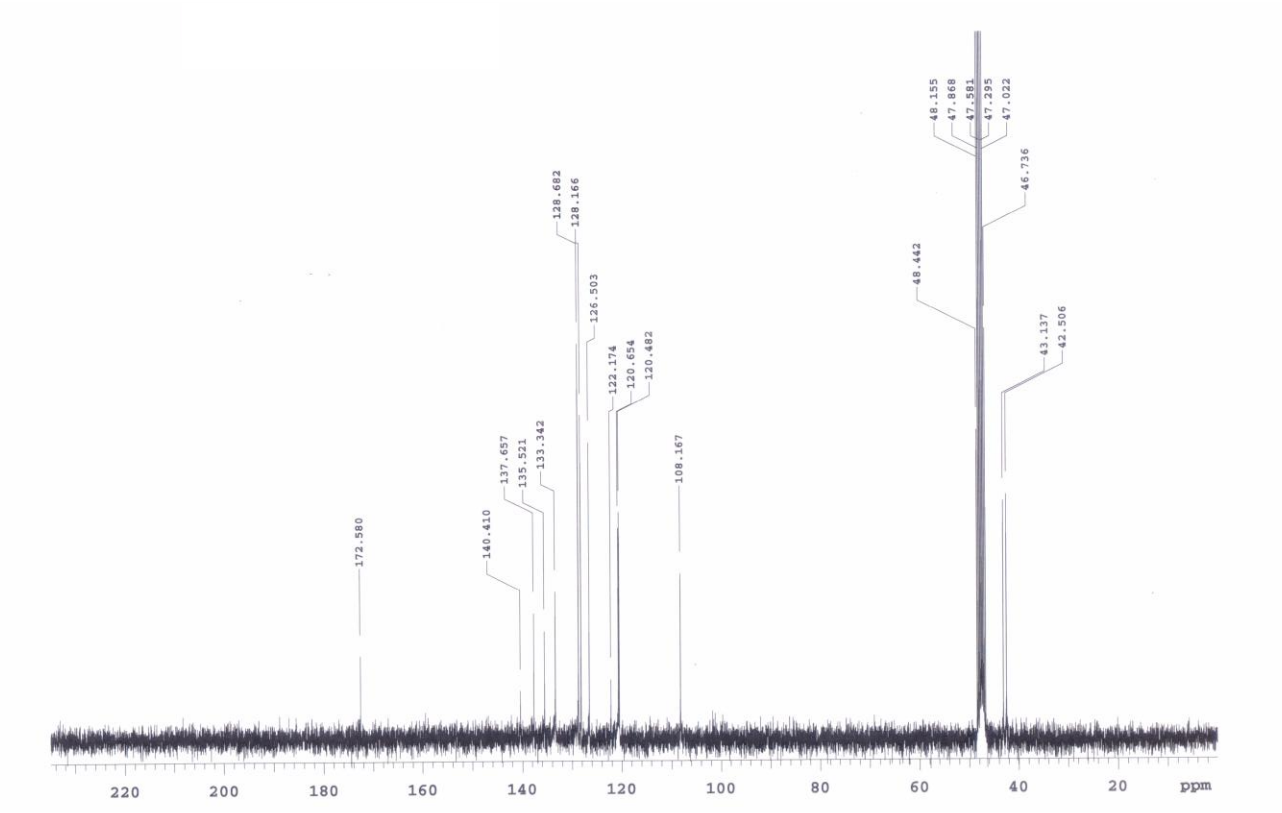

172.580
140.410
137.657
135.521
133.342
128.682
128.166
126.503
122.174
120.654
120.482
108.167
48.442
48.155
47.868
47.581
47.295
47.022
46.736
43.137
42.506
220 200 180 160 140 120 100 80 60 40 20 ppm

Compound **14**

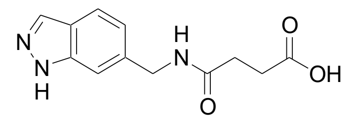
N
N
H
H
N
O
O
OH

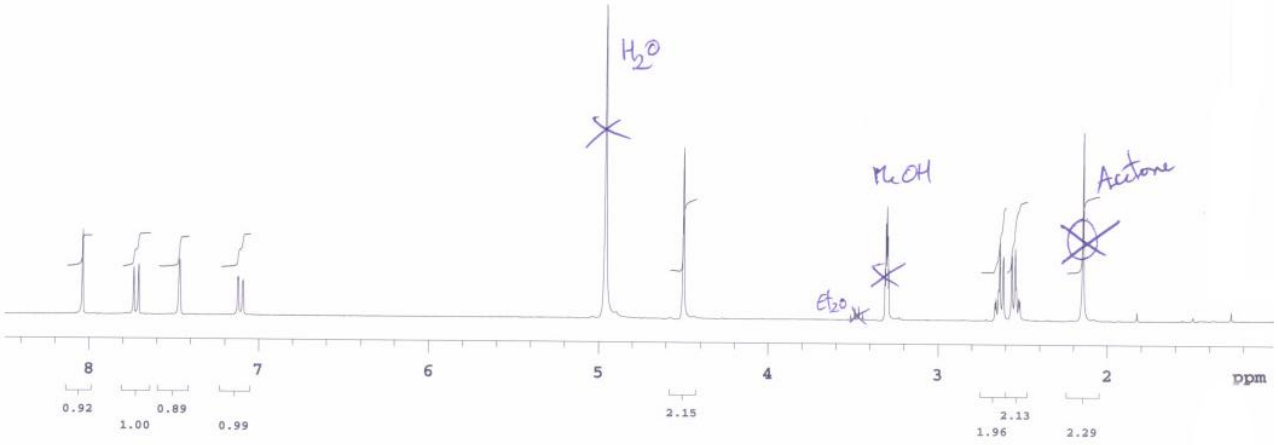
H2O
MeOH
Acetone
Et2O
8
7
6
5
4
3
2
ppm
0.92
1.00
0.89
0.99
2.15
2.13
1.96
2.29

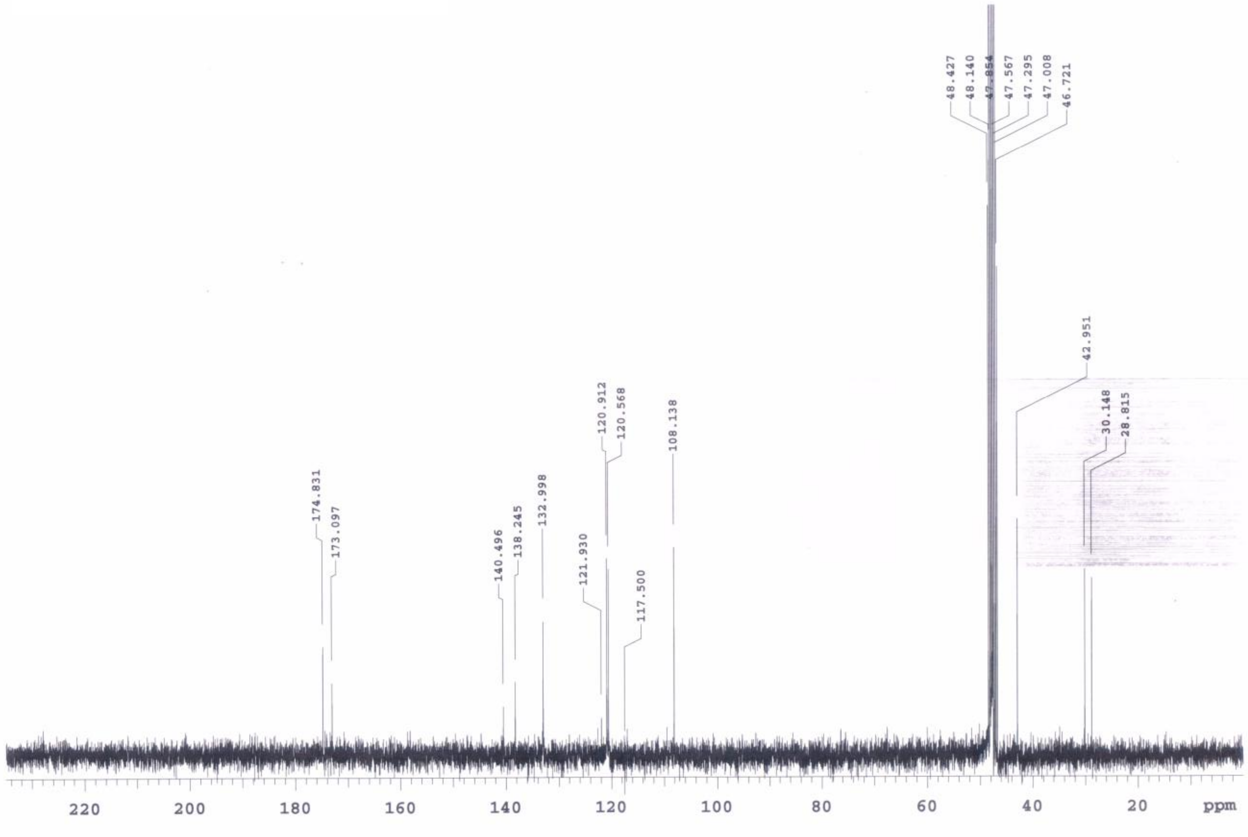
48.427
48.140
47.854
47.567
47.295
47.008
46.721
42.951
30.148
28.815
120.912
120.568
108.138
174.831
173.097
140.496
138.245
132.998
121.930
117.500
220
200
180
160
140
120
100
80
60
40
20
ppm

Compound **16**

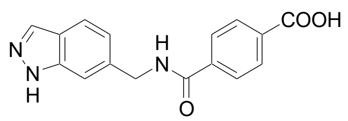

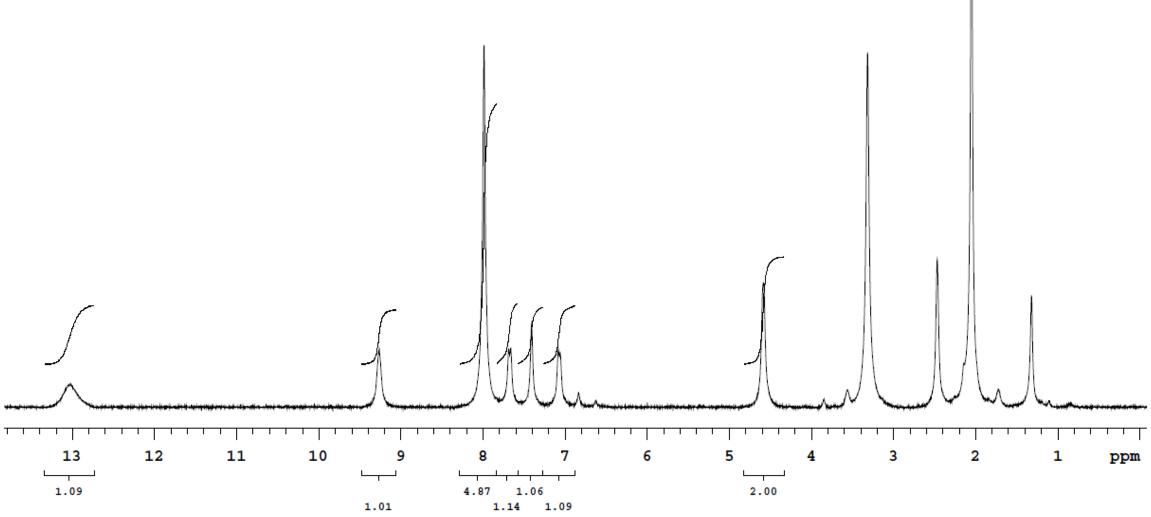

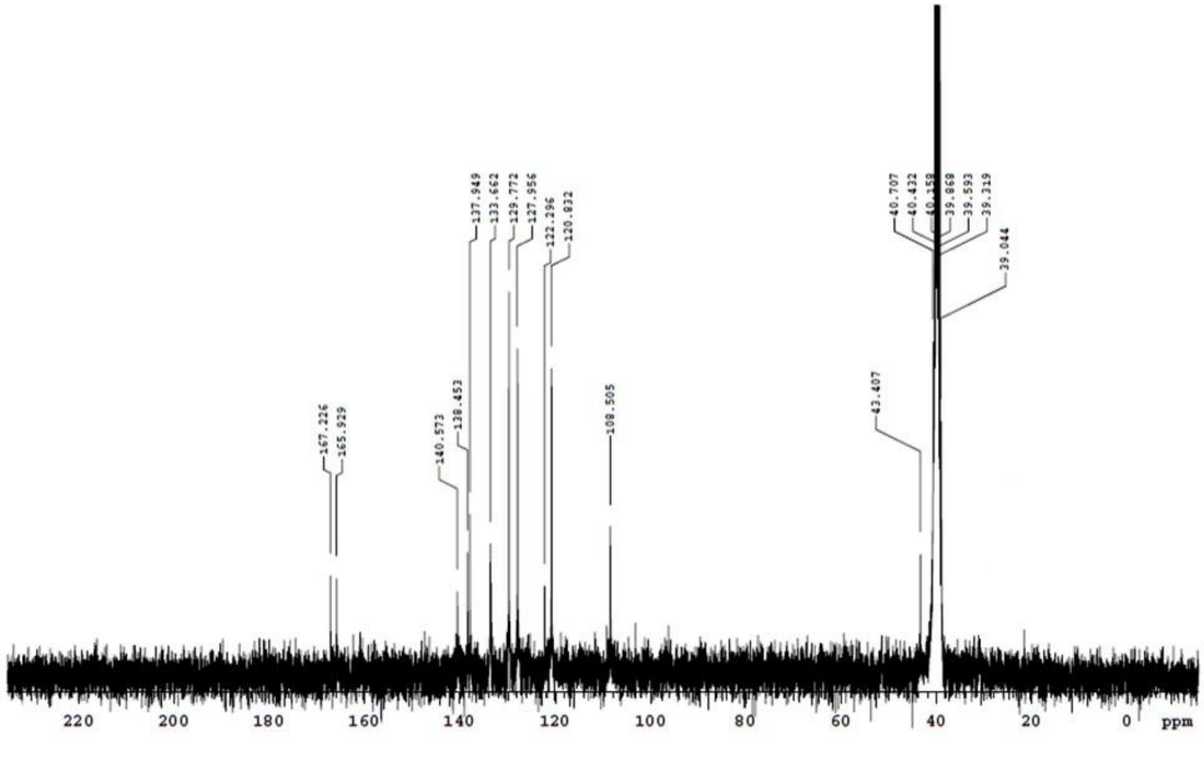

Compound **16**

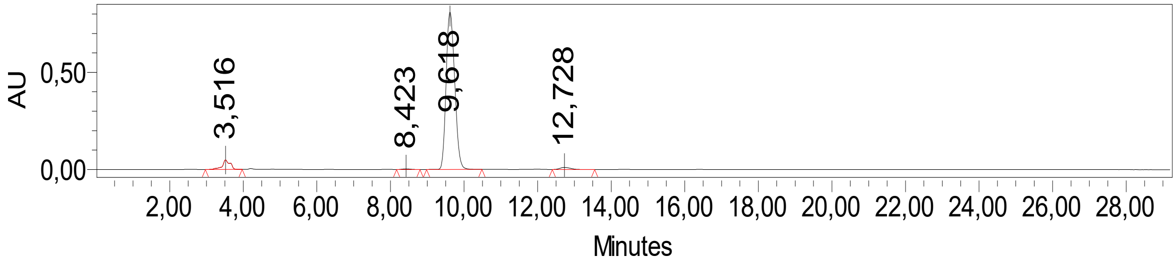

	Name	Retention Time	Area	% Area	Height
1		3,516	458646	3,15	48978
2		8,423	16583	0,11	3164
3		9,618	13922084	95,54	808967
4		12,728	174418	1,20	10093

Compound **6**

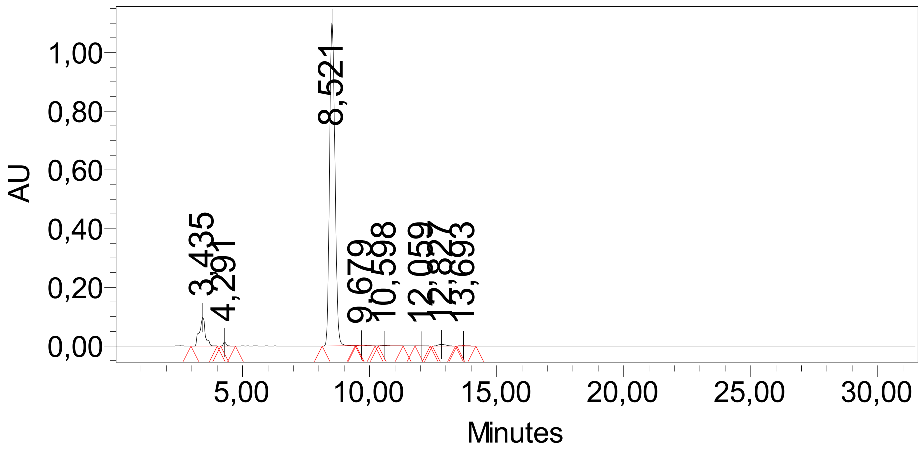

	Retention Time	Area	% Area	Height
1	3,435	512300	2,95	97185
2	4,291	81482	0,47	12902
3	8,521	16588796	95,67	1102066
4	9,679	32562	0,19	2581
5	10,598	12324	0,07	1291
6	12,059	4435	0,03	527
7	12,827	94264	0,54	5381
8	13,693	12661	0,07	904

Compound **10**

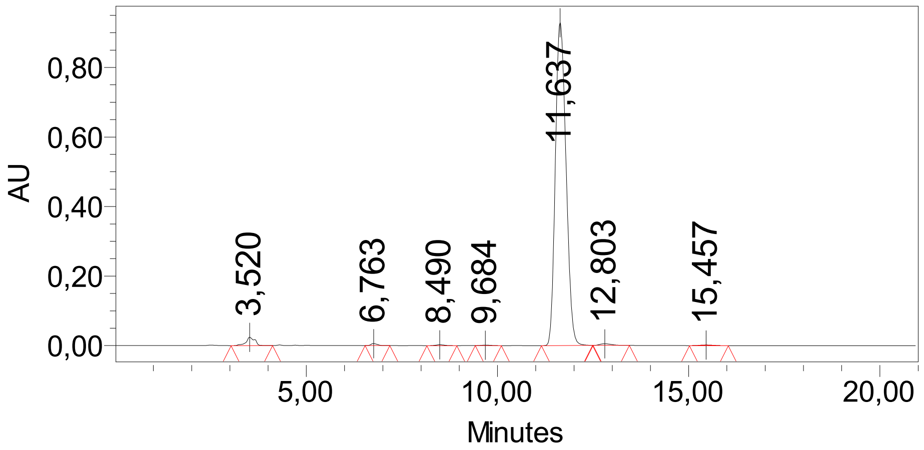

	Retention Time	Area	% Area	Height
1	3,520	386445	2,07	24105
2	6,763	68610	0,37	5995
3	8,490	39577	0,21	2475
4	9,684	25034	0,13	1550
5	11,637	18019837	96,46	929626
6	12,803	96820	0,52	4703
7	15,457	44209	0,24	1786

Compound **7**

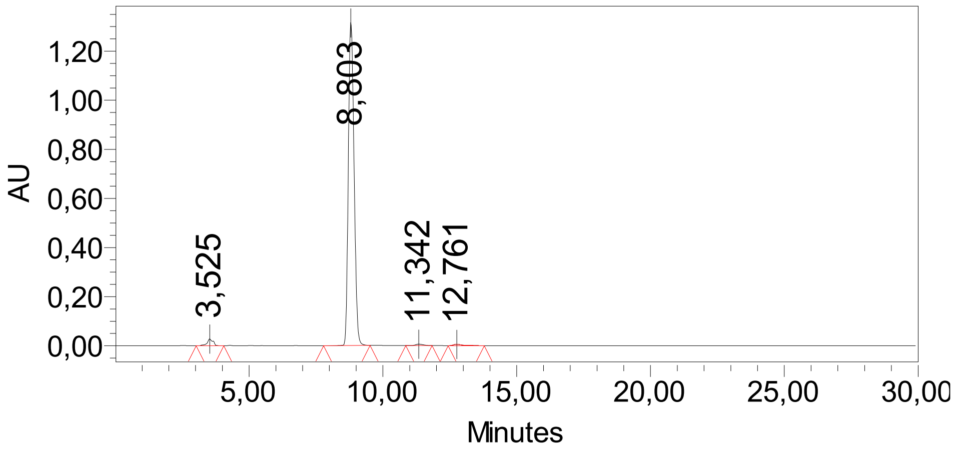

	Retention Time	Area	% Area	Height
1	3,525	434472	2,15	27830
2	8,803	19471101	96,58	1316767
3	11,342	111118	0,55	5592
4	12,761	144516	0,72	5644

Compound **12**

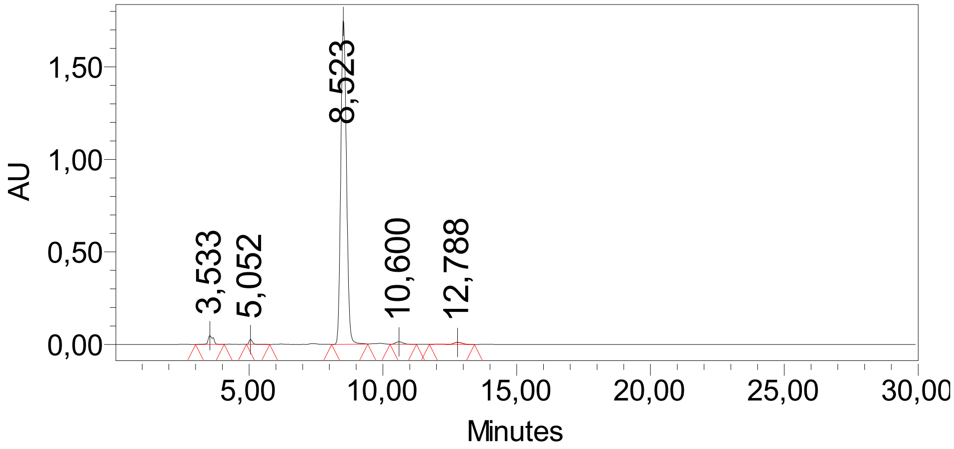

	Retention Time	Area	% Area	Height
1	3,533	635067	2,17	47603
2	5,052	232044	0,79	26376
3	8,523	27833579	95,17	1751331
4	10,600	258058	0,88	13502
5	12,788	286978	0,98	11414

Compound **14**

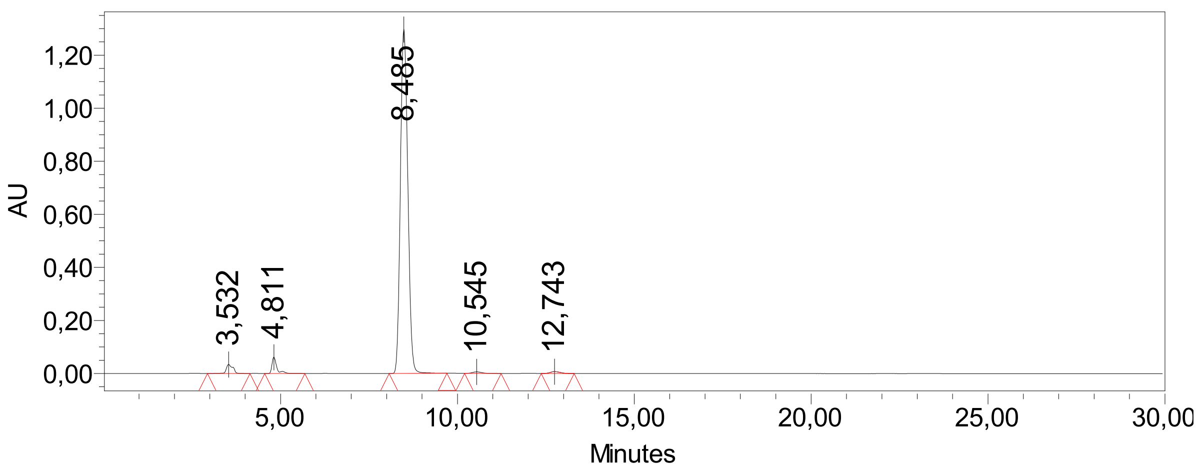

	Retention Time	Area	% Area	Height
1	3,532	314372	1,57	33869
2	4,811	436557	2,19	61499
3	8,485	18992627	95,11	1297977
4	10,545	100394	0,50	5803
5	12,743	125547	0,63	7205

Dose–response curves for iNOS inhibition by compounds 6, 7 and 10

L-citrulline formation, measured as area under the curve (AUC), is plotted as a function of the $Log_{(10)}$ of compound concentration. Data represent raw enzymatic activity. IC_{50} values were determined by nonlinear regression analysis of the activity data using GraphPad Prism.

Compound 6 IC_{50}

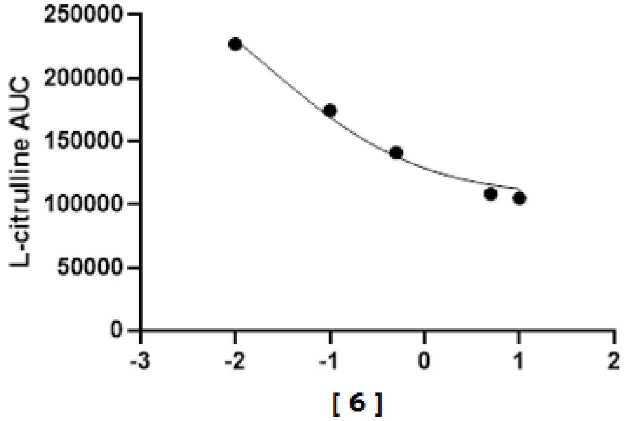

Compound 7 IC_{50}

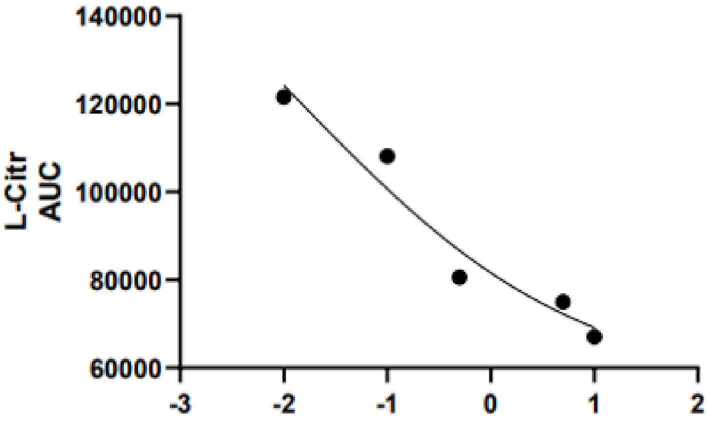

Compound 10 IC_{50}

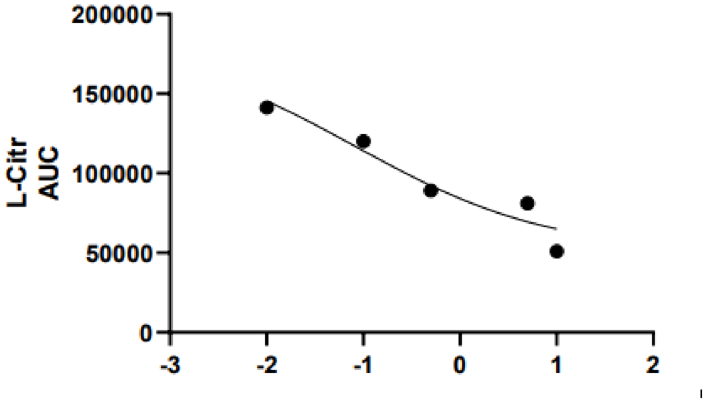

Compound 16 IC_{50}

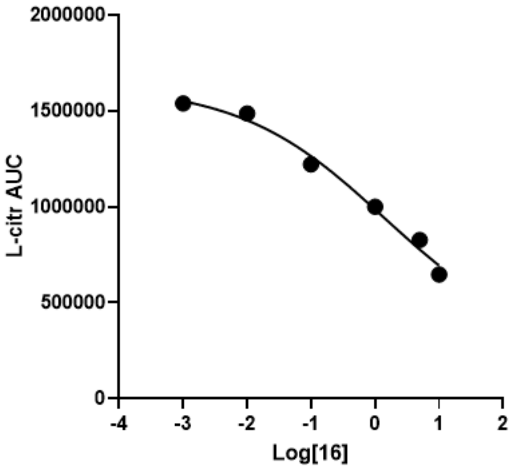

Prediction of physicochemical and pharmacokinetic properties

SwissADME online web tool developed by the molecular modeling group of Swiss Institute of Bioinformatics (SIB)[11] has been used to compute the physicochemical properties and predict the druglikeness and pharmacokinetic properties of the most potent compound **7** and **10** (Table S1). The analysis specifically targeted the neutral forms of the molecules, as passive diffusion across the lipid-rich stratum corneum is predominantly driven by uncharged species.[12,13] It is noteworthy that while psoriatic skin exhibits a perturbed pH compared to healthy skin (shifting from ~4.5 towards a more neutral pH of 6.0–7.0),[14] the favorable permeability profile of the neutral form of lead compound **10** ensures it can effectively reach the intracellular target.

Skin permeability coefficients (*log* K_p) were calculated based on the linear predictive model proposed by Potts and Guy, which mathematically correlates permeability with molecular size and lipophilicity.[15] In general, SwissADME revealed that druglikness prediction was positivefor both **7** and **10**, based on the fulfillment of Lipinski's rule and other filters (e.g. Ghose, Veber, Egan and Muegge). Pharmacokinetic properties appear adequate for the preclinical development. These molecule have a moderate water solubility, the predicted logP (oct/w) is 3.20 and the GI absorption is very high. Importantly, no prediction of BBB permeation emerged, with no presumable Central Nervous System side effects.

Table S2. Evaluation of representative pharmacokinetic parameters of **7** and **10** by means of SwissADME online.

Cpd	Lipophilicity	Water solubility	GI absorption	BBB permanent	P-gp substrate	CYP1A2	Log K_p (skin permeation)	Druglikeness
**7**	1.05	3.47×10^{-2} mg/mL	High	NO	NO	YES	-6.59 cm/s	YES
**10**	1.53	5.87×10^{-3} mg/mL	High	NO	NO	YES	-6.34 cm/s	YES
**BYK191023**	1.90	2.79×10^{-3} mg/mL	High	YES	YES	YES	-6.47 cm/s	YES

REFERENCES

[1] Maccallini, C.; Di Matteo, M.; Ammazzalorso, A.; D'Angelo, A.; De Filippis, B.; Di Silvestre, S.; Fantacuzzi, M.; Giampietro, L.; Pandolfi, A.; Amoroso, R. Reversed-phase high-performance liquid chromatography method with fluorescence detection to screen nitric oxide synthases inhibitors. *J Sep Sci*. 2014, *37*, 1380-1385. doi: 10.1002/jssc.201400059.

[2] Gallorini, M.; Maccallini, C.; Ammazzalorso, A.; Amoia, P.; De Filippis, B.; Fantacuzzi, M.; Giampietro, L.; Cataldi, A.; Amoroso, R. The Selective Acetamidine-Based iNOS Inhibitor CM544 Reduces Glioma Cell Proliferation by Enhancing PARP-1 Cleavage In Vitro. *Int J Mol Sci.* 2019, *20*, 495. doi: 10.3390/ijms20030495.

3] Li, H.; Jamal, J.; Delker, S. L.; Plaza, C.; Ji, H.; Jing, Q.; Huang, H.; Kang, S.; Silverman, R. B.; Poulos, T. L. The Mobility of a Conserved Tyrosine Residue Controls Isoform-Dependent Enzyme–Inhibitor Interactions in Nitric Oxide Synthases. *Biochemistry* **2014**, 53 (32), 5272–5279. https://doi.org/10.1021/bi500561h.

[4] Garcin, E. D.; Arvai, A. S.; Rosenfeld, R. J.; Kroeger, M. D.; Crane, B. R.; Andersson, G.; Andrews, G.; Hamley, P. J.; Mallinder, P. R.; Nicholls, D. J.; St-Gallay, S. A.; Tinker, A. C.; Gensmantel, N. P.; Mete, A.; Cheshire, D. R.; Connolly, S.; Stuehr, D. J.; Aberg, A.; Wallace, A. V.; Tainer, J. A.; Getzoff, E. D. Anchored Plasticity Opens Doors for Selective Inhibitor Design in Nitric Oxide Synthase. *Nat. Chem. Biol*. **2008**, 4 (11), 700–707. https://doi.org/10.1038/nchembio.115.

[5] Roe, D. R.; Cheatham, T. E., III. PTRAJ and CPPTRAJ: Software for Processing and Analysis of Molecular Dynamics Trajectory Data. *J. Chem. Theory Comput*. **2013**, 9 (7), 3084–3095. https://doi.org/10.1021/ct400341p.

[6] Rousseeuw, P. J. Silhouettes: A Graphical Aid to the Interpretation and Validation of Cluster Analysis. *J. Comput. Appl. Math*. **1987**, 20, 53–65. https://doi.org/10.1016/0377-0427(87)90125-7.

[7] Davies, D. L.; Bouldin, D. W. A Cluster Separation Measure. *IEEE Trans. Pattern Anal. Mach. Intell.* **1979**, 1 (2), 224–227. https://doi.org/10.1109/TPAMI.1979.4766909.

S6. Caliński, T.; Harabasz, J. A Dendrite Method for Cluster Analysis. *Commun. Stat. Theory Methods* **1974**, 3 (1), 1–27. https://doi.org/10.1080/03610927408827101.

[8] Miller, B. R., III; McGee, T. D.; Swails, J. M.; Homeyer, N.; Gohlke, H.; Roitberg, A. E. MMPBSA.py: An Efficient Program for End-State Free Energy Calculations. *J. Chem. Theory Comput.* **2012**, 8 (9), 3314–3321. https://doi.org/10.1021/ct300418h.

[9] Genheden, S.; Ryde, U. The MM/PBSA and MM/GBSA Methods to Estimate Ligand-Binding Affinities. *Expert Opin. Drug Discov.* **2015**, 10 (5), 449–461. https://doi.org/10.1517/17460441.2015.1032936.

[10] Onufriev, A.; Bashford, D.; Case, D. A. Modification of the Generalized Born Model Suitable for Macromolecules. *J. Phys. Chem. B* **2000**, 104 (15), 3712–3720. https://doi.org/10.1021/jp994072s.

[11] Daina, A.; Michielin, O.; Zoete, V. SwissADME: a free web tool to evaluate pharmacokinetics, drug-likeness and medicinal chemistry friendliness of small molecules. *Sci. Rep.* **2017**, *7*, 42717. https://doi.org/10.1038/srep42717

[12] Avdeef, A. *Absorption and Drug Development: Solubility, Permeability, and Charge State*, 2nd ed.; John Wiley & Sons: Hoboken, NJ, **2012**. https://doi.org/10.1002/9781118286067

[13] Roberts, M. S.; Cheruvu, H. S.; Mangion, S. E.; et al. Topical drug delivery: History, percutaneous absorption, and product development. *Adv. Drug Deliv. Rev.* **2021**, *177*, 113929. https://doi.org/10.1016/j.addr.2021.113929

[14] Rippke, F.; Schreiner, V.; Schwanitz, H. J. Stratum Corneum pH in Health and Disease: The Acidic Milieu of the Horny Layer: New Findings on the Physiology and Pathophysiology of Skin pH. *Am. J. Clin. Dermatol.* **2002**, *3* (4), 261–272. https://doi.org/10.2165/00128071-200203040-00004

[15] Potts, R. O.; Guy, R. H. Predicting Skin Permeability. *Pharm. Res.* **1992**, *9* (5), 663–669. https://doi.org/10.1023/A:1015810312465
